# Supplementary material for: Diversity in HIV epidemic transitions in India: An application of HIV epidemiological metrices and benchmarks
Source: PLoS One. 2022 Jul 18;17(7):e0270886. doi: 10.1371/journal.pone.0270886 (PMC9292090; doi:10.1371/journal.pone.0270886)
Supplement: S1 Table — (PDF) [file pone.0270886.s001.pdf]

S1 Table. Annual new HIV infections, annual all-cause mortality among PLHIV and total number of PLHIV (in 100,000) by States/UTs in India, 1990-2019

Table a. Annual new HIV infections (in 100,000) by States/UTs in India, 1990-2019

State/UT	Configuration	1990	1991	1992	1993	1994	1995	1996	1997	1998	1999	2000	2001	2002	2003	2004	2005	2006	2007	2008	2009	2010	2011	2012	2013	2014	2015	2016	2017	2018	2019
Andhra Pradesh	Lower bound	0.00046	0.00046	0.00041	0.00035	0.00566	0.03217	0.06785	0.09996	0.11295	0.08129	0.05711	0.03769	0.02940	0.02129	0.01708	0.01395	0.01126	0.00890	0.00700	0.00564	0.00470	0.00381	0.00315	0.00260	0.00211	0.00183	0.00159	0.00136	0.00119	0.00106
Andhra Pradesh	Point	0.00642	0.01315	0.02462	0.03692	0.05199	0.08159	0.11843	0.14478	0.15658	0.12592	0.09433	0.06080	0.04361	0.03078	0.02402	0.01935	0.01614	0.01318	0.01090	0.00929	0.00804	0.00697	0.00605	0.00518	0.00455	0.00415	0.00377	0.00342	0.00307	0.00282
Andhra Pradesh	Upper bound	0.03851	0.05202	0.06879	0.09535	0.12273	0.17325	0.20095	0.21348	0.22439	0.18432	0.13512	0.08660	0.06265	0.04363	0.03322	0.02623	0.02141	0.01765	0.01478	0.01308	0.01210	0.01108	0.01013	0.00907	0.00843	0.00800	0.00768	0.00738	0.00701	0.00669
Arunachal Pradesh	Lower bound	0.00000	0.00000	0.00000	0.00001	0.00001	0.00001	0.00001	0.00001	0.00001	0.00001	0.00001	0.00001	0.00001	0.00002	0.00002	0.00002	0.00002	0.00002	0.00002	0.00003	0.00003	0.00003	0.00003	0.00003	0.00003	0.00003	0.00003	0.00003	0.00003	0.00003
Arunachal Pradesh	Point	0.00001	0.00001	0.00001	0.00001	0.00001	0.00001	0.00001	0.00001	0.00001	0.00002	0.00002	0.00002	0.00002	0.00003	0.00003	0.00003	0.00003	0.00004	0.00004	0.00004	0.00005	0.00005	0.00005	0.00006	0.00006	0.00006	0.00007	0.00007	0.00007	0.00007
Arunachal Pradesh	Upper bound	0.00003	0.00001	0.00001	0.00001	0.00001	0.00002	0.00002	0.00002	0.00002	0.00003	0.00003	0.00003	0.00004	0.00004	0.00005	0.00006	0.00006	0.00006	0.00006	0.00007	0.00007	0.00008	0.00008	0.00009	0.00010	0.00010	0.00011	0.00012	0.00013	0.00014
Assam	Lower bound	0.00023	0.00027	0.00032	0.00035	0.00035	0.00040	0.00047	0.00053	0.00059	0.00068	0.00076	0.00083	0.00091	0.00100	0.00111	0.00118	0.00123	0.00133	0.00138	0.00143	0.00145	0.00148	0.00147	0.00145	0.00139	0.00132	0.00125	0.00119	0.00112	0.00101
Assam	Point	0.00035	0.00039	0.00045	0.00049	0.00047	0.00053	0.00062	0.00071	0.00080	0.00092	0.00103	0.00112	0.00122	0.00130	0.00141	0.00146	0.00152	0.00160	0.00165	0.00169	0.00172	0.00174	0.00176	0.00174	0.00169	0.00164	0.00157	0.00152	0.00144	0.00133
Assam	Upper bound	0.00118	0.00058	0.00068	0.00071	0.00069	0.00078	0.00090	0.00102	0.00112	0.00126	0.00137	0.00145	0.00154	0.00163	0.00174	0.00178	0.00182	0.00190	0.00193	0.00198	0.00201	0.00204	0.00207	0.00206	0.00203	0.00201	0.00199	0.00196	0.00190	0.00178
Bihar	Lower bound	0.00059	0.00069	0.00088	0.00109	0.00133	0.00130	0.00152	0.00185	0.00219	0.00256	0.00298	0.00342	0.00381	0.00416	0.00468	0.00508	0.00553	0.00597	0.00626	0.00660	0.00666	0.00679	0.00673	0.00650	0.00610	0.00560	0.00511	0.00458	0.00391	0.00323
Bihar	Point	0.00078	0.00095	0.00119	0.00149	0.00182	0.00180	0.00212	0.00259	0.00312	0.00374	0.00443	0.00512	0.00581	0.00646	0.00725	0.00794	0.00869	0.00942	0.00999	0.01062	0.01101	0.01144	0.01161	0.01145	0.01101	0.01065	0.01018	0.00958	0.00892	0.00804
Bihar	Upper bound	0.00335	0.00117	0.00149	0.00187	0.00229	0.00229	0.00276	0.00343	0.00423	0.00509	0.00601	0.00686	0.00782	0.00872	0.00983	0.01085	0.01200	0.01320	0.01399	0.01487	0.01569	0.01648	0.01670	0.01674	0.01652	0.01622	0.01577	0.01512	0.01446	0.01347

Chhattishgarh	Lower bound	0.0030	0.0057	0.0121	0.0248	0.0441	0.0687	0.0870	0.0885	0.0708	0.0484	0.0349	0.0262	0.0220	0.0213	0.0215	0.0214	0.0209	0.0203	0.0199	0.0204	0.0196	0.0192	0.0190	0.0185	0.0182	0.0174	0.0177	0.0168	0.0169	0.0170
Chhattishgarh	Point	0.0050	0.0084	0.0161	0.0305	0.0547	0.0854	0.1064	0.1068	0.0909	0.0656	0.0460	0.0342	0.0285	0.0269	0.0260	0.0255	0.0253	0.0252	0.0255	0.0270	0.0266	0.0271	0.0273	0.0274	0.0275	0.0270	0.0278	0.0273	0.0278	0.0283
Chhattishgarh	Upper bound	0.0083	0.0130	0.0275	0.0612	0.1225	0.1720	0.1667	0.1439	0.1239	0.0907	0.0626	0.0450	0.0364	0.0336	0.0329	0.0319	0.0323	0.0329	0.0339	0.0365	0.0365	0.0378	0.0384	0.0386	0.0391	0.0388	0.0402	0.0400	0.0406	0.0411
Delhi	Lower bound	0.0036	0.0045	0.0055	0.0068	0.0081	0.0099	0.0116	0.0136	0.0159	0.0181	0.0207	0.0228	0.0251	0.0268	0.0289	0.0314	0.0331	0.0350	0.0356	0.0364	0.0362	0.0364	0.0360	0.0353	0.0337	0.0321	0.0280	0.0281	0.0238	0.0195
Delhi	Point	0.0061	0.0073	0.0089	0.0105	0.0122	0.0142	0.0161	0.0186	0.0213	0.0238	0.0269	0.0295	0.0324	0.0346	0.0367	0.0395	0.0416	0.0436	0.0448	0.0462	0.0467	0.0471	0.0471	0.0465	0.0449	0.0432	0.0389	0.0393	0.0348	0.0299
Delhi	Upper bound	0.0090	0.0106	0.0124	0.0146	0.0166	0.0192	0.0219	0.0251	0.0282	0.0312	0.0349	0.0383	0.0415	0.0436	0.0460	0.0492	0.0518	0.0543	0.0558	0.0578	0.0590	0.0606	0.0608	0.0615	0.0604	0.0588	0.0542	0.0552	0.0508	0.0455
Goa	Lower bound	0.0002	0.0006	0.0017	0.0053	0.0128	0.0116	0.0080	0.0061	0.0052	0.0045	0.0035	0.0026	0.0015	0.0011	0.0008	0.0007	0.0005	0.0005	0.0004	0.0004	0.0003	0.0003	0.0002	0.0002	0.0002	0.0002	0.0002	0.0002	0.0001	0.0001
Goa	Point	0.0015	0.0051	0.0150	0.0369	0.0476	0.0320	0.0175	0.0101	0.0078	0.0064	0.0054	0.0045	0.0028	0.0023	0.0019	0.0016	0.0013	0.0014	0.0013	0.0011	0.0010	0.0009	0.0008	0.0008	0.0007	0.0007	0.0006	0.0006	0.0006	0.0006
Goa	Upper bound	0.0039	0.0219	0.0937	0.1030	0.0990	0.0685	0.0466	0.0295	0.0189	0.0133	0.0098	0.0076	0.0045	0.0039	0.0034	0.0031	0.0028	0.0033	0.0033	0.0030	0.0029	0.0028	0.0027	0.0026	0.0025	0.0025	0.0025	0.0025	0.0024	0.0024
Gujarat	Lower bound	0.0021	0.0033	0.0052	0.0081	0.0134	0.0204	0.0303	0.0430	0.0550	0.0681	0.0823	0.0953	0.1018	0.1063	0.0911	0.0786	0.0655	0.0587	0.0529	0.0480	0.0443	0.0413	0.0387	0.0368	0.0332	0.0328	0.0312	0.0297	0.0280	0.0262
Gujarat	Point	0.0034	0.0054	0.0086	0.0139	0.0227	0.0346	0.0506	0.0706	0.0874	0.1063	0.1198	0.1292	0.1292	0.1260	0.1108	0.0986	0.0814	0.0721	0.0629	0.0561	0.0520	0.0489	0.0462	0.0440	0.0406	0.0400	0.0387	0.0373	0.0356	0.0337
Gujarat	Upper bound	0.0064	0.0104	0.0162	0.0257	0.0420	0.0654	0.0948	0.1234	0.1449	0.1604	0.1656	0.1642	0.1575	0.1518	0.1329	0.1195	0.1011	0.0893	0.0768	0.0677	0.0617	0.0576	0.0548	0.0526	0.0496	0.0498	0.0488	0.0482	0.0467	0.0452
Himachal Pradesh	Lower bound	0.0018	0.0021	0.0023	0.0026	0.0030	0.0032	0.0034	0.0037	0.0039	0.0041	0.0043	0.0043	0.0043	0.0042	0.0043	0.0043	0.0044	0.0046	0.0047	0.0048	0.0045	0.0043	0.0019	0.0019	0.0018	0.0016	0.0014	0.0014	0.0012	0.0011
Himachal Pradesh	Point	0.0028	0.0032	0.0035	0.0038	0.0042	0.0045	0.0047	0.0050	0.0052	0.0055	0.0057	0.0057	0.0058	0.0056	0.0057	0.0057	0.0058	0.0062	0.0063	0.0064	0.0062	0.0058	0.0025	0.0025	0.0025	0.0023	0.0020	0.0019	0.0018	0.0016
Himachal Pradesh	Upper bound	0.0039	0.0045	0.0048	0.0051	0.0056	0.0059	0.0061	0.0064	0.0066	0.0070	0.0072	0.0071	0.0073	0.0070	0.0072	0.0071	0.0072	0.0077	0.0080	0.0083	0.0082	0.0077	0.0048	0.0049	0.0048	0.0041	0.0027	0.0026	0.0025	0.0024
Haryana	Lower bound	0.0006	0.0010	0.0014	0.0023	0.0035	0.0053	0.0077	0.0110	0.0157	0.0213	0.0286	0.0362	0.0442	0.0476	0.0430	0.0360	0.0315	0.0266	0.0262	0.0258	0.0243	0.0233	0.0220	0.0208	0.0195	0.0188	0.0183	0.0182	0.0181	0.0172

Haryana	Point	0.00015	0.00024	0.00037	0.00059	0.00089	0.00136	0.00203	0.00287	0.00397	0.00497	0.00598	0.00644	0.00648	0.00598	0.00542	0.00460	0.00403	0.00333	0.00320	0.00313	0.00299	0.00288	0.00278	0.00269	0.00260	0.00258	0.00255	0.00260	0.00267	0.00262
Haryana	Upper bound	0.00028	0.00045	0.00072	0.00118	0.00184	0.00291	0.00437	0.00608	0.00755	0.00842	0.00889	0.00877	0.00851	0.00792	0.00719	0.00599	0.00525	0.00435	0.00406	0.00393	0.00373	0.00354	0.00342	0.00338	0.00335	0.00340	0.00343	0.00356	0.00368	0.00366
Jharkhand	Lower bound	0.00014	0.00016	0.00020	0.00024	0.00029	0.00027	0.00032	0.00038	0.00046	0.00052	0.00059	0.00066	0.00075	0.00082	0.00086	0.00094	0.00098	0.00103	0.00103	0.00104	0.00102	0.00102	0.00099	0.00095	0.00091	0.00082	0.00078	0.00073	0.00066	0.00060
Jharkhand	Point	0.00020	0.00023	0.00028	0.00035	0.00042	0.00039	0.00048	0.00056	0.00068	0.00081	0.00093	0.00104	0.00118	0.00129	0.00134	0.00146	0.00153	0.00163	0.00166	0.00172	0.00173	0.00174	0.00174	0.00172	0.00168	0.00158	0.00152	0.00146	0.00139	0.00130
Jharkhand	Upper bound	0.00097	0.00029	0.00036	0.00045	0.00056	0.00054	0.00067	0.00079	0.00097	0.00115	0.00131	0.00146	0.00167	0.00182	0.00193	0.00212	0.00221	0.00242	0.00252	0.00265	0.00271	0.00277	0.00280	0.00278	0.00277	0.00269	0.00266	0.00262	0.00254	0.00241
Jammu & Kashmir	Lower bound	0.00007	0.00007	0.00008	0.00009	0.00010	0.00011	0.00009	0.00010	0.00011	0.00013	0.00014	0.00015	0.00017	0.00020	0.00020	0.00022	0.00023	0.00024	0.00025	0.00026	0.00028	0.00029	0.00014	0.00014	0.00015	0.00015	0.00013	0.00012	0.00011	0.00010
Jammu & Kashmir	Point	0.00012	0.00013	0.00015	0.00017	0.00019	0.00021	0.00017	0.00019	0.00020	0.00023	0.00026	0.00027	0.00031	0.00035	0.00036	0.00038	0.00040	0.00041	0.00043	0.00046	0.00049	0.00051	0.00047	0.00046	0.00042	0.00041	0.00039	0.00037	0.00035	0.00032
Jammu & Kashmir	Upper bound	0.00065	0.00019	0.00022	0.00026	0.00030	0.00035	0.00029	0.00033	0.00036	0.00041	0.00047	0.00049	0.00055	0.00062	0.00063	0.00065	0.00068	0.00068	0.00073	0.00075	0.00082	0.00084	0.00084	0.00083	0.00083	0.00089	0.00090	0.00092	0.00092	0.00092
Karnataka	Lower bound	0.00389	0.01030	0.01500	0.02323	0.02898	0.04441	0.06411	0.08267	0.08844	0.06179	0.04615	0.03479	0.02669	0.01992	0.01615	0.01289	0.00989	0.00757	0.00578	0.00450	0.00352	0.00278	0.00221	0.00181	0.00151	0.00124	0.00105	0.00091	0.00081	0.00070
Karnataka	Point	0.01055	0.02343	0.02614	0.04071	0.05140	0.07633	0.10144	0.11816	0.12020	0.10293	0.08077	0.05817	0.04161	0.02951	0.02287	0.01815	0.01450	0.01158	0.00934	0.00760	0.00632	0.00527	0.00434	0.00365	0.00317	0.00273	0.00240	0.00208	0.00185	0.00157
Karnataka	Upper bound	0.05405	0.07374	0.06930	0.10602	0.12532	0.15287	0.15873	0.15811	0.15460	0.13532	0.11475	0.08823	0.06462	0.04596	0.03443	0.02607	0.02027	0.01628	0.01348	0.01135	0.00977	0.00856	0.00732	0.00640	0.00582	0.00528	0.00478	0.00436	0.00403	0.00353
Kerala	Lower bound	0.00006	0.00010	0.00016	0.00027	0.00044	0.00071	0.00107	0.00161	0.00219	0.00277	0.00312	0.00303	0.00224	0.00180	0.00149	0.00127	0.00110	0.00095	0.00084	0.00078	0.00076	0.00071	0.00066	0.00063	0.00060	0.00057	0.00055	0.00053	0.00051	0.00050
Kerala	Point	0.00013	0.00022	0.00037	0.00060	0.00099	0.00155	0.00229	0.00335	0.00436	0.00481	0.00484	0.00447	0.00342	0.00268	0.00213	0.00173	0.00150	0.00134	0.00122	0.00113	0.00106	0.00100	0.00096	0.00092	0.00089	0.00087	0.00085	0.00084	0.00082	0.00080
Kerala	Upper bound	0.00046	0.00084	0.00147	0.00243	0.00379	0.00564	0.00646	0.00693	0.00746	0.00791	0.00773	0.00687	0.00508	0.00401	0.00318	0.00250	0.00211	0.00190	0.00175	0.00167	0.00170	0.00164	0.00164	0.00165	0.00163	0.00161	0.00159	0.00160	0.00160	0.00159
Meghalaya	Lower bound	0.00000	0.00001	0.00002	0.00002	0.00002	0.00003	0.00004	0.00006	0.00006	0.00007	0.00008	0.00009	0.00011	0.00016	0.00023	0.00031	0.00042	0.00056	0.00073	0.00092	0.00111	0.00126	0.00125	0.00112	0.00097	0.00086	0.00075	0.00069	0.00063	0.00057
Meghalaya	Point	0.00007	0.00007	0.00008	0.00008	0.00006	0.00006	0.00007	0.00007	0.00007	0.00008	0.00009	0.00011	0.00013	0.00019	0.00028	0.00039	0.00056	0.00076	0.00100	0.00124	0.00143	0.00153	0.00152	0.00143	0.00128	0.00114	0.00099	0.00090	0.00081	0.00073

Meghalaya	Upper bound	0.00026	0.00025	0.00024	0.00023	0.00012	0.00011	0.00010	0.00010	0.00009	0.00010	0.00011	0.00013	0.00015	0.00022	0.00035	0.00050	0.00075	0.00101	0.00130	0.00157	0.00172	0.00177	0.00173	0.00167	0.00157	0.00147	0.00133	0.00122	0.00111	0.00098
Maharashtra	Lower bound	0.07430	0.08437	0.07910	0.07408	0.06699	0.06044	0.05329	0.04584	0.03921	0.03341	0.02829	0.02401	0.02032	0.01753	0.01520	0.01327	0.01162	0.01030	0.00931	0.00852	0.00803	0.00735	0.00707	0.00661	0.00564	0.00550	0.00525	0.00494	0.00459	0.00377
Maharashtra	Point	0.17185	0.14413	0.12644	0.11184	0.09632	0.08393	0.07340	0.06385	0.05530	0.04778	0.04121	0.03574	0.03097	0.02694	0.02371	0.02104	0.01876	0.01700	0.01563	0.01454	0.01386	0.01307	0.01258	0.01190	0.01071	0.01073	0.01054	0.01021	0.00982	0.00854
Maharashtra	Upper bound	0.84739	0.57104	0.41486	0.31715	0.18584	0.11811	0.09810	0.08586	0.07570	0.06665	0.05852	0.05186	0.04535	0.04071	0.03724	0.03416	0.03083	0.02891	0.02743	0.02602	0.02537	0.02431	0.02398	0.02320	0.02167	0.02181	0.02170	0.02160	0.02149	0.02010
Manipur	Lower bound	0.00002	0.00014	0.00066	0.00150	0.00290	0.00413	0.00518	0.00455	0.00396	0.00361	0.00283	0.00223	0.00205	0.00182	0.00164	0.00144	0.00130	0.00118	0.00113	0.00108	0.00096	0.00090	0.00083	0.00079	0.00074	0.00068	0.00063	0.00060	0.00054	0.00048
Manipur	Point	0.00061	0.00182	0.00410	0.00631	0.00833	0.00970	0.01006	0.00901	0.00702	0.00547	0.00391	0.00302	0.00274	0.00246	0.00219	0.00194	0.00178	0.00163	0.00159	0.00150	0.00135	0.00130	0.00125	0.00121	0.00117	0.00113	0.00109	0.00108	0.00103	0.00096
Manipur	Upper bound	0.00975	0.01600	0.02084	0.02210	0.02253	0.01839	0.01665	0.01459	0.01179	0.00885	0.00564	0.00405	0.00355	0.00313	0.00276	0.00245	0.00225	0.00215	0.00222	0.00221	0.00204	0.00200	0.00195	0.00189	0.00184	0.00183	0.00181	0.00181	0.00180	0.00169
Madhya Pradesh	Lower bound	0.00008	0.00020	0.00029	0.00046	0.00082	0.00141	0.00224	0.00320	0.00444	0.00613	0.00674	0.00743	0.00705	0.00639	0.00551	0.00453	0.00374	0.00319	0.00294	0.00275	0.00255	0.00236	0.00222	0.00212	0.00196	0.00185	0.00177	0.00168	0.00163	0.00160
Madhya Pradesh	Point	0.00023	0.00045	0.00064	0.00091	0.00146	0.00231	0.00358	0.00512	0.00710	0.00961	0.01028	0.01050	0.00928	0.00807	0.00707	0.00582	0.00480	0.00409	0.00381	0.00361	0.00346	0.00330	0.00320	0.00312	0.00300	0.00295	0.00291	0.00287	0.00287	0.00289
Madhya Pradesh	Upper bound	0.00073	0.00105	0.00113	0.00136	0.00220	0.00365	0.00619	0.00924	0.01267	0.01650	0.01622	0.01597	0.01345	0.01101	0.00927	0.00739	0.00601	0.00516	0.00491	0.00473	0.00470	0.00461	0.00461	0.00459	0.00453	0.00457	0.00461	0.00465	0.00474	0.00477
Mizoram	Lower bound	0.00000	0.00001	0.00002	0.00004	0.00008	0.00017	0.00036	0.00064	0.00085	0.00087	0.00086	0.00089	0.00093	0.00096	0.00099	0.00103	0.00106	0.00102	0.00093	0.00099	0.00108	0.00113	0.00114	0.00115	0.00112	0.00110	0.00109	0.00106	0.00095	0.00085
Mizoram	Point	0.00008	0.00015	0.00033	0.00067	0.00075	0.00086	0.00099	0.00121	0.00142	0.00141	0.00130	0.00131	0.00131	0.00132	0.00133	0.00136	0.00140	0.00132	0.00118	0.00126	0.00138	0.00145	0.00149	0.00152	0.00152	0.00152	0.00155	0.00155	0.00145	0.00137
Mizoram	Upper bound	0.00432	0.00395	0.00394	0.00413	0.00399	0.00368	0.00359	0.00351	0.00382	0.00366	0.00329	0.00293	0.00258	0.00225	0.00193	0.00180	0.00183	0.00175	0.00156	0.00164	0.00176	0.00184	0.00191	0.00195	0.00195	0.00199	0.00202	0.00206	0.00200	0.00196
Nagaland	Lower bound	0.00001	0.00001	0.00005	0.00012	0.00036	0.00084	0.00141	0.00157	0.00145	0.00143	0.00144	0.00153	0.00162	0.00151	0.00133	0.00111	0.00113	0.00117	0.00113	0.00114	0.00118	0.00116	0.00115	0.00114	0.00111	0.00114	0.00111	0.00112	0.00112	0.00104
Nagaland	Point	0.00015	0.00041	0.00102	0.00153	0.00214	0.00265	0.00284	0.00265	0.00216	0.00209	0.00208	0.00215	0.00214	0.00210	0.00183	0.00150	0.00152	0.00156	0.00154	0.00157	0.00165	0.00164	0.00162	0.00162	0.00161	0.00163	0.00160	0.00163	0.00163	0.00155
Nagaland	Upper bound	0.00609	0.00698	0.00700	0.00707	0.00737	0.00742	0.00707	0.00685	0.00548	0.00450	0.00391	0.00347	0.00309	0.00285	0.00241	0.00197	0.00199	0.00203	0.00198	0.00200	0.00213	0.00212	0.00211	0.00211	0.00211	0.00213	0.00210	0.00213	0.00215	0.00205

Odisha	Lower bound	0.0006	0.0011	0.0019	0.0024	0.0036	0.0056	0.0081	0.0116	0.0159	0.0217	0.0290	0.0373	0.0452	0.0523	0.0572	0.0560	0.0498	0.0415	0.0366	0.0327	0.0279	0.0255	0.0234	0.0217	0.0199	0.0188	0.0180	0.0171	0.0166	0.0162
Odisha	Point	0.0013	0.0023	0.0036	0.0043	0.0064	0.0096	0.0137	0.0197	0.0269	0.0368	0.0478	0.0588	0.0674	0.0726	0.0730	0.0692	0.0628	0.0530	0.0469	0.0413	0.0352	0.0322	0.0297	0.0276	0.0254	0.0242	0.0233	0.0227	0.0222	0.0217
Odisha	Upper bound	0.0032	0.0050	0.0069	0.0080	0.0122	0.0188	0.0271	0.0386	0.0509	0.0673	0.0845	0.0960	0.1021	0.1013	0.0982	0.0918	0.0807	0.0681	0.0602	0.0530	0.0455	0.0409	0.0370	0.0345	0.0320	0.0309	0.0304	0.0304	0.0302	0.0297
Punjab	Lower bound	0.0018	0.0027	0.0043	0.0069	0.0105	0.0161	0.0238	0.0349	0.0485	0.0648	0.0812	0.0827	0.0715	0.0618	0.0538	0.0448	0.0398	0.0370	0.0333	0.0311	0.0291	0.0276	0.0261	0.0244	0.0227	0.0209	0.0203	0.0187	0.0171	0.0140
Punjab	Point	0.0039	0.0063	0.0103	0.0164	0.0255	0.0388	0.0561	0.0780	0.0981	0.1144	0.1219	0.1188	0.1057	0.0888	0.0750	0.0606	0.0508	0.0469	0.0420	0.0395	0.0373	0.0359	0.0345	0.0330	0.0316	0.0304	0.0298	0.0286	0.0271	0.0237
Punjab	Upper bound	0.0171	0.0281	0.0485	0.0763	0.1164	0.1513	0.1662	0.1728	0.1694	0.1635	0.1623	0.1620	0.1405	0.1183	0.1016	0.0824	0.0687	0.0619	0.0551	0.0523	0.0489	0.0483	0.0474	0.0467	0.0459	0.0451	0.0458	0.0450	0.0438	0.0410
Rajasthan	Lower bound	0.0049	0.0059	0.0073	0.0089	0.0093	0.0101	0.0122	0.0142	0.0168	0.0190	0.0212	0.0234	0.0256	0.0269	0.0284	0.0308	0.0314	0.0328	0.0336	0.0343	0.0341	0.0336	0.0322	0.0306	0.0286	0.0256	0.0231	0.0209	0.0186	0.0158
Rajasthan	Point	0.0064	0.0077	0.0095	0.0117	0.0124	0.0135	0.0163	0.0191	0.0223	0.0249	0.0277	0.0305	0.0333	0.0354	0.0376	0.0406	0.0420	0.0442	0.0459	0.0473	0.0474	0.0472	0.0462	0.0446	0.0425	0.0393	0.0364	0.0338	0.0314	0.0277
Rajasthan	Upper bound	0.0292	0.0096	0.0121	0.0147	0.0155	0.0169	0.0203	0.0236	0.0274	0.0304	0.0337	0.0374	0.0406	0.0432	0.0464	0.0504	0.0522	0.0552	0.0576	0.0596	0.0603	0.0606	0.0597	0.0584	0.0562	0.0528	0.0497	0.0468	0.0441	0.0398
Sikkim	Lower bound	0.0000	0.0000	0.0000	0.0000	0.0000	0.0001	0.0000	0.0001	0.0001	0.0001	0.0001	0.0001	0.0001	0.0001	0.0001	0.0001	0.0001	0.0002	0.0002	0.0002	0.0002	0.0002	0.0001	0.0001	0.0001	0.0001	0.0001	0.0001	0.0001	0.0001
Sikkim	Point	0.0001	0.0001	0.0001	0.0001	0.0001	0.0001	0.0001	0.0001	0.0001	0.0001	0.0001	0.0001	0.0002	0.0002	0.0002	0.0002	0.0002	0.0003	0.0003	0.0003	0.0003	0.0003	0.0002	0.0002	0.0002	0.0002	0.0002	0.0003	0.0002	0.0002
Sikkim	Upper bound	0.0002	0.0002	0.0002	0.0002	0.0002	0.0002	0.0002	0.0002	0.0002	0.0003	0.0003	0.0003	0.0004	0.0004	0.0004	0.0004	0.0004	0.0004	0.0005	0.0005	0.0005	0.0005	0.0005	0.0006	0.0006	0.0006	0.0007	0.0007	0.0008	0.0008
Tamil Nadu	Lower bound	0.0112	0.0196	0.0310	0.0644	0.1326	0.2573	0.2359	0.1903	0.1683	0.1451	0.1288	0.1139	0.0791	0.0655	0.0574	0.0489	0.0435	0.0376	0.0336	0.0305	0.0280	0.0249	0.0227	0.0204	0.0178	0.0165	0.0149	0.0134	0.0120	0.0108
Tamil Nadu	Point	0.0820	0.1562	0.3050	0.5018	0.6592	0.7038	0.6400	0.4333	0.2885	0.2173	0.1821	0.1587	0.1092	0.0914	0.0815	0.0720	0.0652	0.0587	0.0537	0.0502	0.0472	0.0435	0.0411	0.0382	0.0354	0.0334	0.0317	0.0299	0.0282	0.0262
Tamil Nadu	Upper bound	0.15591	0.16336	0.14385	0.17317	0.16226	0.14096	0.12067	0.10691	0.08809	0.05818	0.03904	0.02665	0.01563	0.01274	0.01097	0.00968	0.00886	0.00822	0.00765	0.00733	0.00697	0.00665	0.00638	0.00607	0.00581	0.00562	0.00545	0.00527	0.00510	0.00490
Tripura	Lower bound	0.0000	0.0000	0.0000	0.0000	0.0000	0.0001	0.0001	0.0001	0.0001	0.0001	0.0002	0.0002	0.0002	0.0003	0.0003	0.0004	0.0005	0.0006	0.0007	0.0008	0.0009	0.0011	0.0013	0.0015	0.0016	0.0018	0.0022	0.0023	0.0026	0.0029
Tripura	Point	0.0000	0.0001	0.0001	0.0001	0.0001	0.0001	0.0001	0.0001	0.0001	0.0002	0.0002	0.0003	0.0003	0.0004	0.0004	0.0005	0.0006	0.0007	0.0009	0.0010	0.0012	0.0015	0.0017	0.0020	0.0023	0.0026	0.0031	0.0035	0.0040	0.0045

Tripura	Upper bound	0.00002	0.00002	0.00002	0.00002	0.00002	0.00002	0.00002	0.00002	0.00002	0.00003	0.00003	0.00003	0.00004	0.00004	0.00005	0.00006	0.00008	0.00009	0.00011	0.00013	0.00016	0.00019	0.00023	0.00027	0.00031	0.00036	0.00042	0.00049	0.00057	0.00065
Uttarakhand	Lower bound	0.00012	0.00014	0.00017	0.00019	0.00023	0.00026	0.00030	0.00035	0.00039	0.00044	0.00047	0.00054	0.00056	0.00061	0.00064	0.00065	0.00068	0.00071	0.00069	0.00069	0.00067	0.00064	0.00062	0.00058	0.00053	0.00049	0.00045	0.00041	0.00036	0.00031
Uttarakhand	Point	0.00026	0.00030	0.00036	0.00040	0.00048	0.00052	0.00058	0.00064	0.00069	0.00074	0.00077	0.00083	0.00082	0.00087	0.00088	0.00089	0.00091	0.00094	0.00092	0.00092	0.00091	0.00088	0.00086	0.00081	0.00075	0.00071	0.00066	0.00061	0.00055	0.00049
Uttarakhand	Upper bound	0.00044	0.00050	0.00058	0.00064	0.00074	0.00078	0.00083	0.00091	0.00095	0.00102	0.00105	0.00114	0.00111	0.00116	0.00117	0.00118	0.00120	0.00124	0.00122	0.00123	0.00122	0.00120	0.00117	0.00113	0.00107	0.00102	0.00097	0.00091	0.00084	0.00077
Uttar Pradesh	Lower bound	0.00296	0.00363	0.00427	0.00498	0.00554	0.00634	0.00699	0.00761	0.00809	0.00866	0.00886	0.00902	0.00956	0.00946	0.00913	0.00945	0.00955	0.00929	0.00910	0.00904	0.00866	0.00832	0.00788	0.00731	0.00670	0.00618	0.00560	0.00504	0.00449	0.00393
Uttar Pradesh	Point	0.00463	0.00554	0.00644	0.00745	0.00829	0.00931	0.01005	0.01094	0.01145	0.01225	0.01260	0.01284	0.01360	0.01347	0.01296	0.01340	0.01354	0.01334	0.01319	0.01304	0.01254	0.01215	0.01158	0.01087	0.01017	0.00946	0.00870	0.00801	0.00742	0.00672
Uttar Pradesh	Upper bound	0.00671	0.00792	0.00913	0.01046	0.01144	0.01271	0.01386	0.01506	0.01582	0.01681	0.01712	0.01730	0.01847	0.01837	0.01777	0.01863	0.01900	0.01891	0.01891	0.01882	0.01803	0.01770	0.01698	0.01595	0.01509	0.01427	0.01328	0.01228	0.01153	0.01060
West Bengal	Lower bound	0.00032	0.00134	0.00545	0.01564	0.02834	0.02318	0.00981	0.00595	0.00425	0.00371	0.00342	0.00338	0.00357	0.00373	0.00372	0.00360	0.00322	0.00307	0.00271	0.00252	0.00234	0.00225	0.00222	0.00215	0.00202	0.00192	0.00190	0.00193	0.00191	0.00194
West Bengal	Point	0.00059	0.00206	0.00675	0.01839	0.03189	0.02763	0.01448	0.00799	0.00581	0.00509	0.00465	0.00446	0.00448	0.00461	0.00469	0.00467	0.00430	0.00425	0.00396	0.00394	0.00395	0.00388	0.00389	0.00387	0.00378	0.00379	0.00383	0.00389	0.00392	0.00397
West Bengal	Upper bound	0.00110	0.00543	0.01290	0.03981	0.05095	0.03760	0.02038	0.01066	0.00740	0.00653	0.00597	0.00572	0.00583	0.00600	0.00624	0.00635	0.00598	0.00608	0.00582	0.00602	0.00638	0.00644	0.00656	0.00654	0.00650	0.00654	0.00672	0.00680	0.00693	0.00698
Andaman & Nicobar	Lower bound	0.00001	0.00001	0.00001	0.00001	0.00001	0.00001	0.00001	0.00001	0.00001	0.00001	0.00001	0.00001	0.00001	0.00001	0.00001	0.00001	0.00002	0.00002	0.00002	0.00002	0.00002	0.00002	0.00002	0.00002	0.00002	0.00002	0.00001	0.00001	0.00001	0.00001
Andaman & Nicobar	Point	0.00001	0.00002	0.00002	0.00002	0.00002	0.00002	0.00002	0.00002	0.00002	0.00002	0.00003	0.00003	0.00003	0.00003	0.00003	0.00003	0.00004	0.00004	0.00004	0.00004	0.00004	0.00004	0.00005	0.00005	0.00005	0.00005	0.00005	0.00002	0.00002	0.00002
Andaman & Nicobar	Upper bound	0.00003	0.00003	0.00003	0.00004	0.00004	0.00004	0.00004	0.00004	0.00005	0.00005	0.00006	0.00006	0.00006	0.00007	0.00007	0.00007	0.00008	0.00008	0.00009	0.00009	0.00009	0.00010	0.00011	0.00012	0.00012	0.00014	0.00014	0.00011	0.00012	0.00013
Chandigarh	Lower bound	0.00001	0.00001	0.00002	0.00003	0.00005	0.00007	0.00011	0.00017	0.00023	0.00024	0.00018	0.00017	0.00015	0.00013	0.00012	0.00011	0.00010	0.00008	0.00008	0.00007	0.00006	0.00006	0.00005	0.00005	0.00004	0.00004	0.00004	0.00004	0.00004	0.00004
Chandigarh	Point	0.00002	0.00003	0.00006	0.00011	0.00017	0.00026	0.00035	0.00039	0.00040	0.00035	0.00026	0.00023	0.00019	0.00017	0.00016	0.00015	0.00014	0.00013	0.00012	0.00011	0.00011	0.00011	0.00010	0.00011	0.00010	0.00011	0.00011	0.00011	0.00011	0.00012
Chandigarh	Upper bound	0.00006	0.00019	0.00061	0.00128	0.00132	0.00105	0.00089	0.00070	0.00062	0.00051	0.00038	0.00034	0.00028	0.00024	0.00022	0.00020	0.00019	0.00017	0.00016	0.00016	0.00016	0.00015	0.00015	0.00016	0.00016	0.00017	0.00017	0.00018	0.00019	0.00020

Dadra & Nagar	Lower bound	0.00000	0.00000	0.00000	0.00000	0.00000	0.00000	0.00000	0.00000	0.00001	0.00001	0.00001	0.00001	0.00001	0.00001	0.00001	0.00001	0.00002	0.00002	0.00002	0.00002	0.00003	0.00003	0.00003	0.00004	0.00003	0.00003	0.00004	0.00003	0.00003	0.00003
Dadra & Nagar	Point	0.00000	0.00000	0.00000	0.00000	0.00000	0.00001	0.00001	0.00001	0.00001	0.00001	0.00001	0.00001	0.00001	0.00002	0.00002	0.00002	0.00003	0.00003	0.00003	0.00004	0.00004	0.00005	0.00005	0.00006	0.00006	0.00007	0.00007	0.00007	0.00007	0.00007
Dadra & Nagar	Upper bound	0.00001	0.00001	0.00001	0.00001	0.00001	0.00001	0.00002	0.00002	0.00002	0.00002	0.00002	0.00003	0.00003	0.00003	0.00004	0.00004	0.00005	0.00005	0.00006	0.00006	0.00007	0.00008	0.00008	0.00009	0.00010	0.00011	0.00013	0.00012	0.00013	0.00014
Daman & Diu	Lower bound	0.00000	0.00000	0.00000	0.00000	0.00000	0.00000	0.00001	0.00001	0.00002	0.00002	0.00003	0.00002	0.00002	0.00002	0.00002	0.00002	0.00002	0.00002	0.00002	0.00001	0.00002	0.00002	0.00001	0.00001	0.00001	0.00001	0.00001	0.00001	0.00001	0.00001
Daman & Diu	Point	0.00000	0.00000	0.00000	0.00001	0.00001	0.00002	0.00003	0.00004	0.00005	0.00005	0.00005	0.00004	0.00003	0.00002	0.00003	0.00002	0.00002	0.00002	0.00002	0.00002	0.00002	0.00002	0.00002	0.00002	0.00002	0.00002	0.00002	0.00002	0.00002	0.00002
Daman & Diu	Upper bound	0.00002	0.00004	0.00006	0.00008	0.00008	0.00009	0.00009	0.00009	0.00009	0.00009	0.00008	0.00007	0.00005	0.00005	0.00005	0.00004	0.00003	0.00003	0.00003	0.00003	0.00003	0.00004	0.00004	0.00004	0.00004	0.00004	0.00004	0.00004	0.00004	0.00004
Pondicherry	Lower bound	0.00003	0.00003	0.00004	0.00007	0.00008	0.00009	0.00008	0.00007	0.00008	0.00008	0.00009	0.00009	0.00011	0.00012	0.00013	0.00015	0.00016	0.00016	0.00017	0.00018	0.00019	0.00020	0.00009	0.00009	0.00009	0.00009	0.00009	0.00009	0.00010	0.00010
Pondicherry	Point	0.00009	0.00010	0.00011	0.00012	0.00012	0.00013	0.00012	0.00011	0.00012	0.00014	0.00015	0.00017	0.00019	0.00020	0.00023	0.00027	0.00030	0.00032	0.00035	0.00038	0.00043	0.00047	0.00020	0.00021	0.00022	0.00022	0.00024	0.00024	0.00025	0.00026
Pondicherry	Upper bound	0.00085	0.00153	0.00043	0.00030	0.00023	0.00021	0.00018	0.00016	0.00018	0.00020	0.00022	0.00025	0.00028	0.00030	0.00035	0.00042	0.00048	0.00052	0.00059	0.00067	0.00078	0.00089	0.00040	0.00040	0.00041	0.00043	0.00046	0.00048	0.00053	0.00061
Telangana	Lower bound	0.00770	0.00848	0.01174	0.01842	0.02802	0.03639	0.03572	0.02779	0.02288	0.01919	0.01581	0.01225	0.01087	0.00888	0.00762	0.00651	0.00563	0.00488	0.00417	0.00367	0.00331	0.00296	0.00259	0.00225	0.00197	0.00182	0.00161	0.00152	0.00134	0.00106
Telangana	Point	0.01294	0.01253	0.01700	0.02675	0.03950	0.05021	0.05501	0.05361	0.04453	0.03503	0.02617	0.01848	0.01553	0.01229	0.01069	0.00943	0.00847	0.00757	0.00683	0.00628	0.00587	0.00548	0.00506	0.00462	0.00427	0.00405	0.00375	0.00359	0.00333	0.00282
Telangana	Upper bound	0.06604	0.06364	0.07548	0.07877	0.08342	0.08222	0.07641	0.06837	0.05944	0.05034	0.03971	0.02882	0.02341	0.01811	0.01500	0.01303	0.01168	0.01062	0.00992	0.00939	0.00896	0.00860	0.00822	0.00778	0.00752	0.00737	0.00704	0.00690	0.00663	0.00595

Table b. Annual all-cause mortality among PLHIV (in 100,000) by States/UTs in India, 1990-2019

State/UT	Configuration	1990	1991	1992	1993	1994	1995	1996	1997	1998	1999	2000	2001	2002	2003	2004	2005	2006	2007	2008	2009	2010	2011	2012	2013	2014	2015	2016	2017	2018	2019
Andhra Pradesh	Lower bound	0.00041	0.00088	0.00197	0.00414	0.00850	0.01789	0.03565	0.06314	0.09780	0.14255	0.18765	0.23767	0.28965	0.34220	0.39645	0.43901	0.46135	0.45861	0.40595	0.36102	0.32187	0.28795	0.25009	0.21498	0.17090	0.14918	0.13301	0.11447	0.09711	0.08591
Andhra Pradesh	Point	0.00230	0.00500	0.01022	0.01890	0.03221	0.05319	0.08536	0.12884	0.18291	0.24239	0.30390	0.36506	0.42847	0.48859	0.53892	0.57278	0.58803	0.56933	0.50772	0.45973	0.41768	0.37960	0.34002	0.30250	0.25373	0.23060	0.21261	0.18730	0.16050	0.14200
Andhra Pradesh	Upper bound	0.03201	0.03905	0.04476	0.05251	0.07434	0.12268	0.18654	0.26207	0.34217	0.42890	0.49809	0.55677	0.61324	0.66001	0.70535	0.73114	0.73643	0.69642	0.62600	0.57342	0.52811	0.48634	0.44231	0.39920	0.34476	0.31862	0.29943	0.26929	0.23492	0.21599
Arunachal Pradesh	Lower bound	0.00000	0.00000	0.00000	0.00001	0.00001	0.00001	0.00002	0.00002	0.00002	0.00003	0.00003	0.00004	0.00004	0.00005	0.00006	0.00006	0.00006	0.00006	0.00005	0.00005	0.00006	0.00007	0.00009	0.00009	0.00008	0.00010	0.00010	0.00010	0.00011	0.00009
Arunachal Pradesh	Point	0.00000	0.00001	0.00001	0.00001	0.00002	0.00002	0.00003	0.00004	0.00004	0.00005	0.00006	0.00006	0.00007	0.00008	0.00009	0.00010	0.00010	0.00010	0.00009	0.00010	0.00011	0.00013	0.00016	0.00017	0.00017	0.00019	0.00020	0.00021	0.00023	0.00021
Arunachal Pradesh	Upper bound	0.00001	0.00001	0.00002	0.00002	0.00003	0.00003	0.00004	0.00005	0.00006	0.00007	0.00008	0.00010	0.00011	0.00013	0.00015	0.00017	0.00017	0.00018	0.00017	0.00019	0.00021	0.00025	0.00030	0.00030	0.00031	0.00034	0.00034	0.00037	0.00041	0.00038
Assam	Lower bound	0.00009	0.00018	0.00028	0.00046	0.00067	0.00090	0.00117	0.00143	0.00170	0.00201	0.00234	0.00272	0.00310	0.00347	0.00388	0.00430	0.00454	0.00458	0.00469	0.00471	0.00480	0.00486	0.00519	0.00544	0.00559	0.00585	0.00590	0.00587	0.00589	0.00555
Assam	Point	0.00036	0.00052	0.00071	0.00094	0.00119	0.00146	0.00177	0.00210	0.00247	0.00286	0.00329	0.00376	0.00426	0.00479	0.00536	0.00594	0.00635	0.00645	0.00660	0.00665	0.00679	0.00681	0.00724	0.00749	0.00761	0.00791	0.00790	0.00786	0.00783	0.00742
Assam	Upper bound	0.00074	0.00095	0.00119	0.00152	0.00187	0.00225	0.00267	0.00316	0.00366	0.00421	0.00484	0.00547	0.00612	0.00685	0.00758	0.00827	0.00878	0.00878	0.00898	0.00901	0.00911	0.00909	0.00957	0.00975	0.00983	0.01016	0.01012	0.01013	0.01010	0.00950
Bihar	Lower bound	0.00015	0.00030	0.00052	0.00087	0.00140	0.00203	0.00277	0.00365	0.00463	0.00574	0.00701	0.00848	0.01004	0.01186	0.01383	0.01597	0.01802	0.01837	0.01740	0.01637	0.01605	0.01603	0.01711	0.01717	0.01649	0.01636	0.01588	0.01558	0.01484	0.01356
Bihar	Point	0.00030	0.00056	0.00092	0.00144	0.00214	0.00297	0.00395	0.00514	0.00655	0.00818	0.01003	0.01216	0.01454	0.01730	0.02043	0.02391	0.02738	0.02886	0.02880	0.02868	0.02954	0.03054	0.03369	0.03471	0.03455	0.03530	0.03543	0.03607	0.03573	0.03411
Bihar	Upper bound	0.00073	0.00115	0.00158	0.00214	0.00295	0.00393	0.00513	0.00663	0.00853	0.01080	0.01340	0.01643	0.01991	0.02380	0.02836	0.03321	0.03858	0.04097	0.04197	0.04258	0.04471	0.04744	0.05260	0.05509	0.05625	0.05896	0.05987	0.06176	0.06191	0.06116
Chhattisgarh	Lower bound	0.00010	0.00022	0.00049	0.00105	0.00215	0.00391	0.00623	0.00915	0.01222	0.01548	0.01914	0.02292	0.02649	0.03005	0.03311	0.03522	0.03656	0.03696	0.03469	0.03286	0.03146	0.02932	0.02819	0.02479	0.02071	0.01766	0.01577	0.01388	0.01334	0.01193
Chhattisgarh	Point	0.00020	0.00042	0.00083	0.00160	0.00303	0.00540	0.00871	0.01278	0.01727	0.02195	0.02668	0.03128	0.03525	0.03888	0.04165	0.04347	0.04434	0.04405	0.04100	0.03861	0.03696	0.03472	0.03340	0.03015	0.02617	0.02319	0.02157	0.01986	0.01967	0.01833
Chhattisgarh	Upper bound	0.00059	0.00085	0.00134	0.00253	0.00534	0.00972	0.01585	0.02192	0.02868	0.03472	0.04092	0.04607	0.05095	0.05437	0.05640	0.05747	0.05758	0.05642	0.05201	0.04926	0.04782	0.04475	0.04316	0.03902	0.03481	0.03179	0.03041	0.02909	0.02912	0.02763

Delhi	Lower bound	0.00046	0.00068	0.00094	0.00123	0.00156	0.00193	0.00235	0.00285	0.00341	0.00409	0.00485	0.00578	0.00682	0.00783	0.00860	0.00754	0.00644	0.00715	0.00792	0.00861	0.00861	0.00912	0.00991	0.01023	0.01017	0.01046	0.00895	0.00814	0.00879	0.00875
Delhi	Point	0.00109	0.00141	0.00178	0.00221	0.00270	0.00327	0.00392	0.00466	0.00552	0.00649	0.00759	0.00884	0.01022	0.01158	0.01264	0.01141	0.01012	0.01111	0.01225	0.01315	0.01317	0.01391	0.01516	0.01585	0.01604	0.01623	0.01385	0.01325	0.01460	0.01376
Delhi	Upper bound	0.00170	0.00222	0.00281	0.00349	0.00426	0.00508	0.00601	0.00707	0.00818	0.00952	0.01105	0.01274	0.01462	0.01637	0.01761	0.01619	0.01455	0.01602	0.01747	0.01873	0.01866	0.01972	0.02138	0.02221	0.02267	0.02299	0.02016	0.02066	0.02292	0.02181
Goa	Lower bound	0.00000	0.00001	0.00003	0.00010	0.00025	0.00054	0.00094	0.00159	0.00235	0.00327	0.00429	0.00528	0.00615	0.00663	0.00700	0.00691	0.00593	0.00529	0.00477	0.00401	0.00300	0.00231	0.00189	0.00165	0.00143	0.00129	0.00120	0.00117	0.00111	0.00106
Goa	Point	0.00002	0.00007	0.00023	0.00069	0.00150	0.00253	0.00370	0.00507	0.00662	0.00817	0.00953	0.01058	0.01127	0.01150	0.01142	0.01088	0.00929	0.00827	0.00751	0.00640	0.00522	0.00445	0.00398	0.00360	0.00308	0.00283	0.00265	0.00244	0.00217	0.00199
Goa	Upper bound	0.00006	0.00020	0.00094	0.00258	0.00463	0.00681	0.00909	0.01120	0.01292	0.01430	0.01485	0.01535	0.01538	0.01525	0.01497	0.01437	0.01245	0.01140	0.01077	0.00980	0.00862	0.00781	0.00742	0.00695	0.00637	0.00631	0.00606	0.00576	0.00535	0.00507
Gujarat	Lower bound	0.00014	0.00022	0.00035	0.00055	0.00090	0.00140	0.00218	0.00341	0.00498	0.00710	0.01002	0.01352	0.01781	0.02285	0.02798	0.03216	0.03544	0.03875	0.03536	0.03230	0.02864	0.02709	0.02540	0.02476	0.02330	0.02219	0.02160	0.02053	0.02000	0.01968
Gujarat	Point	0.00024	0.00037	0.00059	0.00094	0.00151	0.00240	0.00374	0.00571	0.00841	0.01198	0.01647	0.02192	0.02813	0.03499	0.04195	0.04733	0.05115	0.05511	0.04964	0.04472	0.03920	0.03631	0.03387	0.03226	0.02990	0.02846	0.02748	0.02611	0.02535	0.02444
Gujarat	Upper bound	0.00039	0.00065	0.00107	0.00173	0.00284	0.00447	0.00687	0.01041	0.01520	0.02127	0.02872	0.03751	0.04674	0.05602	0.06446	0.07028	0.07314	0.07554	0.06604	0.05693	0.04964	0.04609	0.04332	0.04112	0.03837	0.03627	0.03543	0.03394	0.03292	0.03134
Himachal Pradesh	Lower bound	0.00034	0.00047	0.00059	0.00073	0.00085	0.00101	0.00116	0.00133	0.00150	0.00168	0.00186	0.00207	0.00227	0.00245	0.00266	0.00282	0.00284	0.00258	0.00220	0.00207	0.00192	0.00164	0.00150	0.00150	0.00146	0.00137	0.00117	0.00102	0.00094	0.00092
Himachal Pradesh	Point	0.00067	0.00083	0.00100	0.00119	0.00138	0.00158	0.00179	0.00201	0.00224	0.00247	0.00272	0.00296	0.00320	0.00344	0.00368	0.00389	0.00393	0.00364	0.00324	0.00309	0.00287	0.00254	0.00239	0.00239	0.00234	0.00219	0.00169	0.00145	0.00135	0.00132
Himachal Pradesh	Upper bound	0.00100	0.00122	0.00148	0.00175	0.00204	0.00233	0.00261	0.00288	0.00317	0.00349	0.00378	0.00408	0.00439	0.00468	0.00495	0.00517	0.00519	0.00477	0.00427	0.00411	0.00386	0.00346	0.00334	0.00335	0.00333	0.00315	0.00239	0.00210	0.00202	0.00213
Haryana	Lower bound	0.00004	0.00007	0.00011	0.00017	0.00025	0.00039	0.00059	0.00090	0.00132	0.00191	0.00269	0.00373	0.00501	0.00660	0.00852	0.01077	0.01233	0.01388	0.01572	0.01845	0.02010	0.02210	0.02327	0.02288	0.02052	0.01945	0.02058	0.01768	0.01687	0.01758
Haryana	Point	0.00010	0.00016	0.00026	0.00041	0.00065	0.00101	0.00155	0.00235	0.00349	0.00503	0.00700	0.00944	0.01226	0.01540	0.01875	0.02202	0.02398	0.02543	0.02682	0.02881	0.02951	0.03009	0.03010	0.02836	0.02551	0.02418	0.02532	0.02208	0.02152	0.02258
Haryana	Upper bound	0.00018	0.00029	0.00049	0.00080	0.00130	0.00206	0.00327	0.00504	0.00745	0.01053	0.01419	0.01846	0.02297	0.02765	0.03183	0.03523	0.03674	0.03713	0.03757	0.03904	0.03881	0.03912	0.03848	0.03637	0.03287	0.03128	0.03249	0.02831	0.02807	0.02927
Jharkhand	Lower bound	0.00004	0.00009	0.00014	0.00023	0.00037	0.00053	0.00070	0.00089	0.00111	0.00137	0.00164	0.00195	0.00227	0.00262	0.00297	0.00332	0.00338	0.00345	0.00302	0.00278	0.00268	0.00239	0.00236	0.00235	0.00240	0.00236	0.00231	0.00223	0.00226	0.00225
Jharkhand	Point	0.00011	0.00017	0.00026	0.00040	0.00057	0.00077	0.00101	0.00129	0.00162	0.00200	0.00242	0.00289	0.00341	0.00395	0.00453	0.00510	0.00538	0.00564	0.00526	0.00511	0.00513	0.00488	0.00506	0.00525	0.00557	0.00549	0.00552	0.00569	0.00597	0.00593

Jharkhand	Upper bound	0.00026	0.00034	0.00044	0.00059	0.00079	0.00106	0.00138	0.00176	0.00221	0.00277	0.00340	0.00412	0.00487	0.00572	0.00659	0.00748	0.00791	0.00832	0.00798	0.00807	0.00835	0.00832	0.00887	0.00939	0.01015	0.01027	0.01076	0.01127	0.01192	0.01208
Jammu & Kashmir	Lower bound	0.00002	0.00004	0.00006	0.00011	0.00017	0.00023	0.00029	0.00035	0.00041	0.00047	0.00055	0.00062	0.00069	0.00077	0.00084	0.00089	0.00077	0.00064	0.00056	0.00050	0.00056	0.00057	0.00056	0.00059	0.00054	0.00052	0.00052	0.00050	0.00050	0.00050
Jammu & Kashmir	Point	0.00007	0.00012	0.00018	0.00025	0.00034	0.00044	0.00054	0.00066	0.00077	0.00090	0.00102	0.00114	0.00127	0.00140	0.00153	0.00162	0.00149	0.00136	0.00128	0.00120	0.00134	0.00141	0.00145	0.00152	0.00149	0.00150	0.00155	0.00162	0.00170	0.00172
Jammu & Kashmir	Upper bound	0.00018	0.00022	0.00029	0.00037	0.00047	0.00061	0.00077	0.00094	0.00114	0.00136	0.00161	0.00185	0.00212	0.00238	0.00265	0.00285	0.00273	0.00262	0.00255	0.00247	0.00277	0.00287	0.00294	0.00308	0.00309	0.00317	0.00331	0.00344	0.00375	0.00386
Karnataka	Lower bound	0.00113	0.00246	0.00491	0.00954	0.01722	0.03055	0.05102	0.07837	0.11151	0.14758	0.18703	0.23199	0.27622	0.32077	0.36321	0.39385	0.41189	0.40132	0.37627	0.32590	0.27234	0.22285	0.18186	0.15079	0.13132	0.11188	0.09597	0.08399	0.07289	0.05888
Karnataka	Point	0.00347	0.00809	0.01487	0.02516	0.04019	0.06259	0.09409	0.13517	0.18431	0.23883	0.29620	0.35423	0.40810	0.45753	0.49694	0.52064	0.52930	0.50846	0.47543	0.41388	0.35883	0.30525	0.26451	0.22763	0.20307	0.18075	0.15934	0.13850	0.11875	0.09072
Karnataka	Upper bound	0.02148	0.03110	0.04065	0.05720	0.08149	0.12847	0.18999	0.26481	0.34354	0.42241	0.48935	0.55393	0.58726	0.62641	0.64918	0.66729	0.66446	0.63899	0.60335	0.53259	0.47173	0.41066	0.36420	0.32487	0.29833	0.26787	0.24467	0.21585	0.18578	0.14209
Kerala	Lower bound	0.00002	0.00004	0.00008	0.00013	0.00023	0.00038	0.00061	0.00099	0.00154	0.00229	0.00322	0.00452	0.00600	0.00761	0.00942	0.01076	0.00996	0.00931	0.00977	0.00958	0.00905	0.00847	0.00805	0.00732	0.00688	0.00647	0.00605	0.00565	0.00527	0.00483
Kerala	Point	0.00007	0.00011	0.00019	0.00032	0.00053	0.00087	0.00139	0.00219	0.00334	0.00485	0.00670	0.00891	0.01136	0.01379	0.01616	0.01775	0.01610	0.01485	0.01461	0.01400	0.01292	0.01207	0.01156	0.01056	0.01006	0.00950	0.00898	0.00843	0.00785	0.00688
Kerala	Upper bound	0.00021	0.00036	0.00066	0.00119	0.00197	0.00320	0.00495	0.00731	0.01035	0.01381	0.01742	0.02059	0.02319	0.02517	0.02732	0.02716	0.02456	0.02273	0.02226	0.02110	0.01996	0.01911	0.01843	0.01726	0.01646	0.01587	0.01522	0.01469	0.01382	0.01224
Meghalaya	Lower bound	0.00000	0.00000	0.00000	0.00001	0.00002	0.00003	0.00004	0.00007	0.00009	0.00013	0.00017	0.00022	0.00029	0.00036	0.00045	0.00054	0.00065	0.00077	0.00092	0.00108	0.00126	0.00151	0.00184	0.00224	0.00249	0.00279	0.00305	0.00323	0.00322	0.00300
Meghalaya	Point	0.00002	0.00003	0.00006	0.00008	0.00012	0.00016	0.00020	0.00024	0.00028	0.00032	0.00036	0.00041	0.00045	0.00050	0.00057	0.00066	0.00079	0.00095	0.00118	0.00142	0.00174	0.00214	0.00265	0.00322	0.00353	0.00389	0.00414	0.00433	0.00431	0.00403
Meghalaya	Upper bound	0.00009	0.00015	0.00018	0.00020	0.00023	0.00025	0.00029	0.00032	0.00037	0.00041	0.00045	0.00049	0.00053	0.00059	0.00066	0.00078	0.00095	0.00119	0.00153	0.00190	0.00239	0.00299	0.00369	0.00437	0.00473	0.00509	0.00530	0.00544	0.00536	0.00501
Maharashtra	Lower bound	0.01339	0.03061	0.05778	0.09564	0.15719	0.24071	0.34288	0.45598	0.56884	0.65288	0.70938	0.73058	0.70538	0.68017	0.63398	0.58188	0.51600	0.44340	0.35375	0.27211	0.21613	0.17334	0.15036	0.12456	0.10058	0.09331	0.08937	0.08456	0.07858	0.07129
Maharashtra	Point	0.24651	0.33865	0.44389	0.55310	0.65962	0.75541	0.83608	0.89859	0.94161	0.96564	0.97244	0.96493	0.94514	0.91286	0.87118	0.81267	0.74060	0.65341	0.55023	0.45168	0.37993	0.32177	0.28843	0.24137	0.19211	0.18648	0.17827	0.16403	0.15446	0.12868
Maharashtra	Upper bound	0.80708	0.88045	0.92616	0.96729	1.03508	1.09809	1.14626	1.18530	1.22025	1.23606	1.25842	1.24675	1.22550	1.19911	1.14713	1.08762	1.01041	0.91425	0.80461	0.69094	0.59887	0.52755	0.49812	0.43474	0.36727	0.38429	0.37265	0.34935	0.33386	0.26508
Manipur	Lower bound	0.00000	0.00002	0.00006	0.00017	0.00043	0.00106	0.00234	0.00455	0.00747	0.01107	0.01527	0.01946	0.02374	0.02718	0.02973	0.02915	0.02621	0.02300	0.02082	0.01912	0.01840	0.01710	0.01664	0.01543	0.01385	0.01400	0.01270	0.01212	0.01051	0.00910

Manipur	Point	0.00016	0.00048	0.00123	0.00254	0.00451	0.00722	0.01066	0.01467	0.01902	0.02355	0.02801	0.03210	0.03559	0.03837	0.03986	0.03831	0.03443	0.03080	0.02829	0.02627	0.02526	0.02340	0.02266	0.02109	0.01919	0.01940	0.01753	0.01688	0.01459	0.01307
Manipur	Upper bound	0.00724	0.00760	0.01061	0.01335	0.01687	0.02036	0.02529	0.03191	0.03701	0.04123	0.04427	0.04695	0.04877	0.05017	0.05053	0.04793	0.04295	0.03855	0.03596	0.03362	0.03233	0.03001	0.02895	0.02729	0.02531	0.02590	0.02385	0.02368	0.02095	0.01950
Madhya Pradesh	Lower bound	0.00004	0.00008	0.00016	0.00031	0.00057	0.00097	0.00163	0.00268	0.00407	0.00606	0.00845	0.01143	0.01445	0.01777	0.02150	0.02538	0.02778	0.02976	0.03057	0.03165	0.03167	0.03026	0.02871	0.02725	0.02465	0.02287	0.02070	0.01862	0.01755	0.01545
Madhya Pradesh	Point	0.00012	0.00024	0.00042	0.00068	0.00111	0.00179	0.00285	0.00441	0.00665	0.00979	0.01364	0.01817	0.02295	0.02807	0.03335	0.03826	0.04118	0.04332	0.04398	0.04476	0.04414	0.04180	0.03961	0.03762	0.03447	0.03232	0.02961	0.02734	0.02619	0.02363
Madhya Pradesh	Upper bound	0.00069	0.00109	0.00149	0.00201	0.00261	0.00348	0.00474	0.00706	0.01083	0.01619	0.02298	0.03102	0.03872	0.04631	0.05382	0.05959	0.06242	0.06427	0.06327	0.06403	0.06201	0.05872	0.05582	0.05275	0.04879	0.04681	0.04375	0.04124	0.04013	0.03685
Mizoram	Lower bound	0.00000	0.00000	0.00001	0.00001	0.00002	0.00004	0.00007	0.00013	0.00023	0.00040	0.00068	0.00111	0.00183	0.00270	0.00368	0.00483	0.00574	0.00615	0.00620	0.00591	0.00550	0.00531	0.00523	0.00520	0.00453	0.00413	0.00400	0.00383	0.00335	0.00302
Mizoram	Point	0.00003	0.00005	0.00012	0.00024	0.00043	0.00067	0.00100	0.00144	0.00198	0.00263	0.00333	0.00409	0.00490	0.00570	0.00648	0.00723	0.00788	0.00824	0.00834	0.00802	0.00754	0.00735	0.00728	0.00720	0.00644	0.00596	0.00587	0.00568	0.00502	0.00462
Mizoram	Upper bound	0.00337	0.00473	0.00601	0.00729	0.00743	0.00674	0.00584	0.00547	0.00573	0.00572	0.00596	0.00661	0.00751	0.00848	0.00919	0.00990	0.01034	0.01078	0.01092	0.01056	0.01008	0.00976	0.00965	0.00957	0.00869	0.00826	0.00825	0.00813	0.00728	0.00691
Nagaland	Lower bound	0.00000	0.00000	0.00001	0.00002	0.00006	0.00015	0.00034	0.00072	0.00134	0.00202	0.00315	0.00463	0.00663	0.00839	0.00991	0.01063	0.01095	0.01035	0.00934	0.00856	0.00708	0.00602	0.00547	0.00512	0.00471	0.00491	0.00539	0.00503	0.00480	0.00426
Nagaland	Point	0.00004	0.00011	0.00028	0.00061	0.00112	0.00187	0.00286	0.00406	0.00543	0.00697	0.00862	0.01033	0.01205	0.01311	0.01384	0.01401	0.01402	0.01329	0.01227	0.01136	0.00969	0.00858	0.00805	0.00776	0.00729	0.00777	0.00844	0.00798	0.00775	0.00698
Nagaland	Upper bound	0.01053	0.01206	0.01404	0.01390	0.01333	0.01209	0.01193	0.01218	0.01231	0.01276	0.01390	0.01493	0.01619	0.01697	0.01742	0.01763	0.01743	0.01662	0.01544	0.01440	0.01266	0.01140	0.01095	0.01063	0.01015	0.01094	0.01191	0.01134	0.01107	0.01014
Odisha	Lower bound	0.00004	0.00007	0.00011	0.00019	0.00030	0.00049	0.00075	0.00114	0.00166	0.00235	0.00325	0.00445	0.00618	0.00798	0.01010	0.01258	0.01530	0.01792	0.01898	0.02045	0.02086	0.02248	0.02315	0.02239	0.02094	0.02014	0.01923	0.01818	0.01752	0.01647
Odisha	Point	0.00010	0.00017	0.00027	0.00040	0.00060	0.00090	0.00134	0.00196	0.00283	0.00404	0.00566	0.00777	0.01032	0.01333	0.01673	0.02043	0.02424	0.02737	0.02869	0.03051	0.03039	0.03160	0.03160	0.02991	0.02785	0.02643	0.02518	0.02389	0.02305	0.02179
Odisha	Upper bound	0.00034	0.00044	0.00062	0.00087	0.00119	0.00170	0.00255	0.00378	0.00551	0.00780	0.01072	0.01463	0.01889	0.02387	0.02930	0.03431	0.03910	0.04289	0.04247	0.04402	0.04284	0.04401	0.04315	0.04021	0.03707	0.03505	0.03387	0.03221	0.03127	0.02973
Punjab	Lower bound	0.00010	0.00016	0.00025	0.00041	0.00066	0.00104	0.00160	0.00244	0.00363	0.00529	0.00758	0.01055	0.01405	0.01810	0.02251	0.02752	0.03258	0.03656	0.03736	0.03752	0.03756	0.03719	0.03781	0.03385	0.02984	0.02596	0.02384	0.02007	0.01633	0.01351
Punjab	Point	0.00022	0.00036	0.00059	0.00096	0.00155	0.00246	0.00384	0.00586	0.00867	0.01236	0.01693	0.02235	0.02842	0.03484	0.04124	0.04693	0.05190	0.05479	0.05317	0.05081	0.04877	0.04702	0.04696	0.04249	0.03818	0.03415	0.03213	0.02788	0.02360	0.01917
Punjab	Upper bound	0.00077	0.00136	0.00244	0.00419	0.00690	0.01172	0.01856	0.02576	0.03399	0.04292	0.05122	0.05849	0.06431	0.06963	0.07303	0.07557	0.07677	0.07629	0.07044	0.06754	0.06454	0.06240	0.06198	0.05657	0.05231	0.04787	0.04499	0.04034	0.03555	0.03018

Rajasthan	Lower bound	0.00014	0.00029	0.00047	0.00077	0.00117	0.00166	0.00220	0.00283	0.00358	0.00438	0.00523	0.00619	0.00726	0.00846	0.00965	0.01055	0.00952	0.00926	0.00846	0.00786	0.00747	0.00722	0.00715	0.00719	0.00722	0.00715	0.00683	0.00686	0.00672	0.00687
Rajasthan	Point	0.00032	0.00053	0.00084	0.00126	0.00178	0.00240	0.00313	0.00400	0.00498	0.00609	0.00730	0.00865	0.01012	0.01171	0.01339	0.01462	0.01353	0.01337	0.01279	0.01240	0.01214	0.01212	0.01222	0.01239	0.01249	0.01243	0.01220	0.01223	0.01239	0.01227
Rajasthan	Upper bound	0.00074	0.00105	0.00142	0.00193	0.00252	0.00328	0.00417	0.00533	0.00665	0.00811	0.00965	0.01142	0.01335	0.01542	0.01755	0.01920	0.01787	0.01786	0.01742	0.01734	0.01729	0.01754	0.01793	0.01830	0.01844	0.01867	0.01846	0.01872	0.01922	0.01912
Sikkim	Lower bound	0.00000	0.00000	0.00000	0.00001	0.00001	0.00001	0.00001	0.00001	0.00002	0.00002	0.00002	0.00003	0.00003	0.00004	0.00004	0.00005	0.00005	0.00004	0.00003	0.00002	0.00002	0.00002	0.00002	0.00002	0.00002	0.00002	0.00002	0.00002	0.00002	0.00002
Sikkim	Point	0.00001	0.00001	0.00001	0.00001	0.00002	0.00002	0.00003	0.00003	0.00004	0.00004	0.00005	0.00005	0.00006	0.00007	0.00007	0.00008	0.00008	0.00008	0.00006	0.00005	0.00006	0.00006	0.00006	0.00006	0.00006	0.00006	0.00006	0.00006	0.00006	0.00006
Sikkim	Upper bound	0.00001	0.00002	0.00003	0.00003	0.00004	0.00005	0.00006	0.00008	0.00009	0.00010	0.00012	0.00013	0.00015	0.00016	0.00018	0.00019	0.00020	0.00019	0.00015	0.00015	0.00015	0.00015	0.00015	0.00015	0.00015	0.00016	0.00017	0.00018	0.00019	0.00021
Tamil Nadu	Lower bound	0.00019	0.00062	0.00141	0.00296	0.00597	0.01021	0.01846	0.03350	0.05089	0.07312	0.09927	0.13064	0.16473	0.19425	0.20897	0.19720	0.15592	0.13344	0.10575	0.08597	0.07206	0.06051	0.05294	0.04721	0.04205	0.03813	0.03462	0.03203	0.02954	0.02715
Tamil Nadu	Point	0.00309	0.00634	0.01275	0.02388	0.04078	0.06310	0.08994	0.11927	0.15006	0.18115	0.21039	0.23516	0.25321	0.26516	0.26926	0.25577	0.21656	0.19781	0.16850	0.14545	0.12618	0.10824	0.09613	0.08630	0.07434	0.06627	0.05986	0.05368	0.04840	0.04411
Tamil Nadu	Upper bound	0.07931	0.13071	0.19928	0.27467	0.33083	0.34767	0.36219	0.36584	0.35532	0.34357	0.33676	0.34091	0.34777	0.35585	0.35793	0.33922	0.29227	0.27425	0.23946	0.21516	0.19493	0.17413	0.15907	0.14449	0.12525	0.11171	0.10079	0.08869	0.07893	0.07027
Tripura	Lower bound	0.00000	0.00000	0.00000	0.00000	0.00001	0.00001	0.00001	0.00002	0.00002	0.00002	0.00003	0.00004	0.00004	0.00005	0.00006	0.00008	0.00009	0.00009	0.00008	0.00008	0.00009	0.00010	0.00011	0.00011	0.00012	0.00013	0.00015	0.00017	0.00017	0.00019
Tripura	Point	0.00000	0.00000	0.00001	0.00001	0.00001	0.00002	0.00002	0.00003	0.00003	0.00004	0.00004	0.00005	0.00006	0.00008	0.00009	0.00011	0.00013	0.00013	0.00012	0.00012	0.00013	0.00016	0.00016	0.00018	0.00020	0.00022	0.00025	0.00029	0.00031	0.00035
Tripura	Upper bound	0.00001	0.00001	0.00002	0.00003	0.00005	0.00006	0.00007	0.00008	0.00009	0.00010	0.00011	0.00012	0.00013	0.00014	0.00016	0.00018	0.00019	0.00020	0.00017	0.00018	0.00019	0.00022	0.00024	0.00027	0.00030	0.00034	0.00041	0.00048	0.00053	0.00062
Uttarakhand	Lower bound	0.00011	0.00019	0.00030	0.00042	0.00053	0.00064	0.00078	0.00093	0.00106	0.00122	0.00141	0.00160	0.00181	0.00207	0.00237	0.00268	0.00297	0.00303	0.00290	0.00291	0.00285	0.00275	0.00284	0.00292	0.00285	0.00294	0.00294	0.00290	0.00287	0.00260
Uttarakhand	Point	0.00051	0.00066	0.00082	0.00100	0.00120	0.00141	0.00164	0.00189	0.00216	0.00245	0.00276	0.00309	0.00343	0.00381	0.00420	0.00459	0.00494	0.00496	0.00480	0.00471	0.00458	0.00443	0.00456	0.00459	0.00443	0.00455	0.00459	0.00458	0.00448	0.00419
Uttarakhand	Upper bound	0.00087	0.00112	0.00142	0.00174	0.00210	0.00245	0.00280	0.00317	0.00359	0.00397	0.00441	0.00481	0.00519	0.00571	0.00620	0.00667	0.00711	0.00710	0.00689	0.00679	0.00662	0.00646	0.00665	0.00670	0.00647	0.00669	0.00679	0.00682	0.00671	0.00642
Uttar Pradesh	Lower bound	0.00564	0.00779	0.01008	0.01247	0.01501	0.01767	0.02080	0.02393	0.02736	0.03133	0.03552	0.03953	0.04389	0.04778	0.05198	0.05560	0.05580	0.05596	0.05367	0.04957	0.04684	0.04484	0.04344	0.04249	0.04075	0.03838	0.03665	0.03419	0.03206	0.02936
Uttar Pradesh	Point	0.01055	0.01334	0.01655	0.02011	0.02398	0.02820	0.03267	0.03754	0.04273	0.04825	0.05395	0.05982	0.06559	0.07144	0.07713	0.08190	0.08255	0.08300	0.08190	0.07786	0.07483	0.07341	0.07235	0.07079	0.06866	0.06647	0.06344	0.06016	0.05823	0.05430

Uttar Pradesh	Upper bound	0.01535	0.01925	0.02420	0.02954	0.03537	0.04164	0.04794	0.05486	0.06185	0.07002	0.07791	0.08492	0.09272	0.10069	0.10817	0.11582	0.11751	0.11785	0.11729	0.11254	0.11098	0.11083	0.10972	0.10984	0.10797	0.10592	0.10429	0.10062	0.09741	0.09185
West Bengal	Lower bound	0.00007	0.00028	0.00116	0.00377	0.00856	0.01382	0.01920	0.02461	0.03198	0.04031	0.04940	0.05721	0.06327	0.06793	0.07041	0.07039	0.06531	0.06055	0.05467	0.04925	0.04110	0.03344	0.02893	0.02421	0.02010	0.01653	0.01384	0.01190	0.01065	0.01000
West Bengal	Point	0.00014	0.00049	0.00164	0.00495	0.01153	0.01975	0.02784	0.03617	0.04562	0.05560	0.06478	0.07232	0.07748	0.08039	0.08134	0.08003	0.07423	0.06932	0.06382	0.05885	0.05071	0.04337	0.03914	0.03493	0.03061	0.02683	0.02405	0.02151	0.02061	0.01881
West Bengal	Upper bound	0.00025	0.00104	0.00316	0.00905	0.01994	0.03218	0.04398	0.05673	0.07001	0.08187	0.09162	0.09850	0.10301	0.10500	0.10567	0.10331	0.09592	0.08892	0.08282	0.07733	0.06829	0.06006	0.05616	0.05165	0.04742	0.04435	0.04209	0.03991	0.04033	0.03786
Andaman & Nicobar	Lower bound	0.00000	0.00001	0.00001	0.00002	0.00003	0.00003	0.00004	0.00004	0.00005	0.00006	0.00006	0.00007	0.00007	0.00008	0.00008	0.00008	0.00009	0.00009	0.00009	0.00010	0.00010	0.00010	0.00011	0.00011	0.00012	0.00012	0.00010	0.00004	0.00003	0.00003
Andaman & Nicobar	Point	0.00002	0.00003	0.00003	0.00004	0.00005	0.00007	0.00008	0.00009	0.00010	0.00011	0.00012	0.00013	0.00015	0.00016	0.00016	0.00017	0.00018	0.00020	0.00021	0.00022	0.00023	0.00024	0.00026	0.00027	0.00029	0.00031	0.00029	0.00017	0.00017	0.00021
Andaman & Nicobar	Upper bound	0.00004	0.00005	0.00006	0.00008	0.00010	0.00012	0.00014	0.00016	0.00019	0.00021	0.00025	0.00028	0.00030	0.00033	0.00035	0.00037	0.00039	0.00042	0.00044	0.00047	0.00049	0.00053	0.00056	0.00059	0.00063	0.00067	0.00066	0.00047	0.00053	0.00063
Chandigarh	Lower bound	0.00000	0.00000	0.00001	0.00001	0.00002	0.00004	0.00006	0.00009	0.00014	0.00021	0.00029	0.00041	0.00055	0.00071	0.00087	0.00098	0.00089	0.00093	0.00097	0.00107	0.00117	0.00120	0.00109	0.00100	0.00093	0.00089	0.00078	0.00069	0.00056	0.00050
Chandigarh	Point	0.00001	0.00001	0.00002	0.00004	0.00008	0.00013	0.00021	0.00033	0.00047	0.00065	0.00084	0.00106	0.00127	0.00146	0.00161	0.00169	0.00147	0.00143	0.00140	0.00149	0.00153	0.00152	0.00137	0.00127	0.00123	0.00121	0.00116	0.00110	0.00099	0.00100
Chandigarh	Upper bound	0.00001	0.00003	0.00009	0.00026	0.00055	0.00095	0.00142	0.00191	0.00251	0.00296	0.00321	0.00344	0.00363	0.00356	0.00345	0.00319	0.00259	0.00234	0.00210	0.00207	0.00205	0.00194	0.00172	0.00161	0.00157	0.00155	0.00154	0.00152	0.00146	0.00152
Dadra & Nagar	Lower bound	0.00000	0.00000	0.00000	0.00000	0.00000	0.00001	0.00001	0.00001	0.00001	0.00001	0.00002	0.00002	0.00002	0.00003	0.00003	0.00004	0.00004	0.00005	0.00006	0.00006	0.00007	0.00007	0.00007	0.00007	0.00007	0.00007	0.00006	0.00006	0.00006	0.00006
Dadra & Nagar	Point	0.00000	0.00001	0.00001	0.00001	0.00001	0.00001	0.00002	0.00002	0.00002	0.00003	0.00003	0.00004	0.00004	0.00005	0.00006	0.00007	0.00008	0.00009	0.00010	0.00011	0.00012	0.00013	0.00013	0.00013	0.00014	0.00014	0.00013	0.00011	0.00011	0.00011
Dadra & Nagar	Upper bound	0.00001	0.00001	0.00002	0.00002	0.00003	0.00003	0.00004	0.00005	0.00006	0.00007	0.00008	0.00009	0.00011	0.00012	0.00014	0.00016	0.00017	0.00019	0.00022	0.00023	0.00025	0.00026	0.00025	0.00026	0.00027	0.00027	0.00024	0.00023	0.00023	0.00025
Daman & Diu	Lower bound	0.00000	0.00000	0.00000	0.00000	0.00000	0.00000	0.00000	0.00000	0.00001	0.00001	0.00002	0.00003	0.00004	0.00005	0.00007	0.00010	0.00013	0.00015	0.00018	0.00016	0.00015	0.00015	0.00012	0.00010	0.00009	0.00008	0.00007	0.00006	0.00005	0.00005
Daman & Diu	Point	0.00000	0.00000	0.00000	0.00000	0.00001	0.00001	0.00002	0.00003	0.00004	0.00006	0.00008	0.00011	0.00014	0.00017	0.00020	0.00023	0.00025	0.00027	0.00028	0.00023	0.00021	0.00021	0.00017	0.00015	0.00014	0.00014	0.00011	0.00009	0.00008	0.00008
Daman & Diu	Upper bound	0.00001	0.00001	0.00003	0.00005	0.00008	0.00012	0.00016	0.00020	0.00024	0.00027	0.00030	0.00032	0.00034	0.00034	0.00036	0.00037	0.00038	0.00039	0.00039	0.00032	0.00030	0.00030	0.00024	0.00022	0.00022	0.00022	0.00018	0.00015	0.00013	0.00013
Pondicherry	Lower bound	0.00001	0.00003	0.00005	0.00009	0.00014	0.00019	0.00024	0.00029	0.00034	0.00040	0.00046	0.00051	0.00055	0.00062	0.00068	0.00072	0.00058	0.00039	0.00034	0.00032	0.00033	0.00034	0.00035	0.00044	0.00051	0.00062	0.00081	0.00091	0.00103	0.00126

Pondicherry	Point	0.00005	0.00009	0.00013	0.00018	0.00024	0.00031	0.00039	0.00046	0.00054	0.00062	0.00070	0.00077	0.00085	0.00093	0.00102	0.00109	0.00094	0.00073	0.00071	0.00072	0.00080	0.00086	0.00101	0.00126	0.00148	0.00181	0.00222	0.00241	0.00262	0.00294
Pondicherry	Upper bound	0.00029	0.00048	0.00067	0.00090	0.00114	0.00131	0.00146	0.00156	0.00164	0.00164	0.00163	0.00158	0.00154	0.00150	0.00150	0.00156	0.00140	0.00119	0.00123	0.00134	0.00153	0.00168	0.00198	0.00249	0.00290	0.00352	0.00424	0.00457	0.00500	0.00555
Telangana	Lower bound	0.00187	0.00359	0.00624	0.01095	0.01862	0.03018	0.04442	0.06063	0.07818	0.09740	0.11537	0.13348	0.15137	0.16714	0.18344	0.19155	0.19108	0.18150	0.15634	0.13452	0.11600	0.10325	0.08942	0.08015	0.06526	0.05766	0.04999	0.04530	0.04136	0.03166
Telangana	Point	0.00488	0.00849	0.01343	0.02103	0.03256	0.04820	0.06774	0.08996	0.11376	0.13812	0.16175	0.18342	0.20436	0.22269	0.23633	0.24350	0.24404	0.23242	0.20561	0.18230	0.16384	0.15015	0.13560	0.12376	0.10657	0.09907	0.09066	0.08575	0.07839	0.05364
Telangana	Upper bound	0.02288	0.03205	0.04838	0.07279	0.10752	0.15538	0.19497	0.22448	0.25129	0.26981	0.27748	0.28516	0.29181	0.29599	0.30316	0.30741	0.30677	0.29282	0.26661	0.24332	0.22347	0.20678	0.19029	0.17795	0.15919	0.15162	0.14084	0.13451	0.12675	0.08781

Table c. PLHIV (in 100,000) by States/UTs in India, 1990-2019

State/UT	Configuration	1990	1991	1992	1993	1994	1995	1996	1997	1998	1999	2000	2001	2002	2003	2004	2005	2006	2007	2008	2009	2010	2011	2012	2013	2014	2015	2016	2017	2018	2019
Andhra Pradesh	Lower bound	0.0192	0.0413	0.0997	0.2438	0.4456	0.7989	1.5090	2.6206	3.9761	5.1856	5.9778	6.4017	6.5571	6.3661	6.1049	5.7590	5.3244	4.8674	4.4913	4.1706	3.8753	3.6183	3.3911	3.2026	3.0761	2.9269	2.7903	2.6713	2.5646	2.4782
Andhra Pradesh	Point	0.1101	0.2366	0.4726	0.8229	1.3106	2.0733	3.1722	4.4911	5.8740	6.8909	7.5303	7.7733	7.7809	7.6001	7.3014	6.9221	6.4954	6.0579	5.6592	5.2923	4.9551	4.6452	4.3656	4.1149	3.9067	3.7176	3.5427	3.3895	3.2598	3.1460
Andhra Pradesh	Upper bound	0.5416	0.6773	1.0985	1.9638	3.0578	4.5429	6.1767	7.5456	8.6122	9.1366	9.3872	9.4371	9.2766	9.0140	8.7431	8.2812	7.8615	7.3548	6.9097	6.4993	6.0848	5.7162	5.3795	5.1263	4.9187	4.6731	4.4871	4.3241	4.1844	4.0559
Arunachal Pradesh	Lower bound	0.0001	0.0001	0.0002	0.0002	0.0003	0.0003	0.0004	0.0004	0.0005	0.0006	0.0006	0.0007	0.0008	0.0010	0.0011	0.0012	0.0014	0.0015	0.0017	0.0020	0.0022	0.0024	0.0026	0.0029	0.0031	0.0034	0.0036	0.0038	0.0040	0.0042
Arunachal Pradesh	Point	0.0002	0.0002	0.0003	0.0004	0.0005	0.0006	0.0006	0.0007	0.0008	0.0009	0.0010	0.0012	0.0013	0.0015	0.0017	0.0019	0.0021	0.0024	0.0027	0.0030	0.0034	0.0037	0.0041	0.0045	0.0049	0.0053	0.0058	0.0063	0.0068	0.0073
Arunachal Pradesh	Upper bound	0.0002	0.0003	0.0004	0.0005	0.0006	0.0007	0.0009	0.0010	0.0012	0.0014	0.0016	0.0018	0.0021	0.0024	0.0028	0.0031	0.0035	0.0039	0.0044	0.0048	0.0053	0.0058	0.0063	0.0067	0.0073	0.0079	0.0087	0.0095	0.0104	0.0114
Assam	Lower bound	0.0081	0.0107	0.0135	0.0165	0.0194	0.0223	0.0260	0.0302	0.0349	0.0401	0.0455	0.0513	0.0573	0.0637	0.0706	0.0779	0.0860	0.0954	0.1054	0.1164	0.1279	0.1387	0.1499	0.1604	0.1695	0.1780	0.1848	0.1903	0.1961	0.2013
Assam	Point	0.0124	0.0158	0.0196	0.0235	0.0271	0.0309	0.0353	0.0403	0.0458	0.0522	0.0592	0.0666	0.0745	0.0827	0.0914	0.1001	0.1089	0.1185	0.1284	0.1386	0.1490	0.1596	0.1699	0.1798	0.1891	0.1975	0.2054	0.2127	0.2193	0.2252
Assam	Upper bound	0.0179	0.0226	0.0279	0.0335	0.0386	0.0437	0.0496	0.0569	0.0646	0.0730	0.0813	0.0902	0.0987	0.1073	0.1160	0.1249	0.1334	0.1426	0.1512	0.1610	0.1710	0.1815	0.1918	0.2015	0.2109	0.2199	0.2296	0.2389	0.2499	0.2591
Bihar	Lower bound	0.0099	0.0167	0.0249	0.0350	0.0474	0.0586	0.0710	0.0861	0.1038	0.1236	0.1477	0.1736	0.2016	0.2300	0.2608	0.2948	0.3316	0.3735	0.4194	0.4712	0.5243	0.5682	0.6278	0.6711	0.7158	0.7563	0.7964	0.8283	0.8532	0.8731
Bihar	Point	0.0137	0.0226	0.0336	0.0470	0.0631	0.0781	0.0953	0.1161	0.1408	0.1701	0.2044	0.2434	0.2869	0.3342	0.3863	0.4418	0.5012	0.5666	0.6376	0.7152	0.7957	0.8796	0.9620	1.0418	1.1173	1.1885	1.2549	1.3146	1.3680	1.4144
Bihar	Upper bound	0.0178	0.0283	0.0414	0.0567	0.0759	0.0944	0.1159	0.1433	0.1773	0.2187	0.2657	0.3176	0.3779	0.4418	0.5100	0.5873	0.6711	0.7612	0.8564	0.9686	1.0736	1.1934	1.3046	1.4127	1.5179	1.6182	1.7085	1.7978	1.8782	1.9520
Chhattisgarh	Lower bound	0.0044	0.0099	0.0220	0.0466	0.0916	0.1566	0.2389	0.3200	0.3850	0.4254	0.4436	0.4473	0.4427	0.4345	0.4214	0.4035	0.3831	0.3649	0.3498	0.3366	0.3250	0.3185	0.3119	0.3069	0.3046	0.3035	0.3044	0.3061	0.3078	0.3132

Chhattisgarh	Point	0.0085	0.0165	0.0317	0.0606	0.1123	0.1923	0.2900	0.3840	0.4576	0.5012	0.5206	0.5234	0.5167	0.5047	0.4891	0.4712	0.4521	0.4333	0.4178	0.4062	0.3959	0.3883	0.3822	0.3794	0.3807	0.3845	0.3907	0.3982	0.4063	0.4163
Chhattisgarh	Upper bound	0.0141	0.0236	0.0465	0.1073	0.2235	0.3899	0.5259	0.6070	0.6550	0.6782	0.6889	0.6739	0.6642	0.6425	0.6160	0.5912	0.5645	0.5385	0.5206	0.5063	0.4914	0.4854	0.4824	0.4823	0.4853	0.4956	0.5083	0.5226	0.5317	0.5429
Delhi	Lower bound	0.0173	0.0211	0.0258	0.0314	0.0380	0.0456	0.0538	0.0648	0.0777	0.0928	0.1100	0.1282	0.1471	0.1663	0.1861	0.2105	0.2388	0.2675	0.2948	0.3257	0.3576	0.3884	0.4154	0.4398	0.4628	0.4837	0.5025	0.5231	0.5377	0.5486
Delhi	Point	0.0294	0.0354	0.0424	0.0507	0.0602	0.0712	0.0834	0.0973	0.1131	0.1304	0.1497	0.1704	0.1925	0.2155	0.2395	0.2676	0.2991	0.3316	0.3642	0.3973	0.4308	0.4640	0.4960	0.5266	0.5555	0.5824	0.6075	0.6335	0.6538	0.6699
Delhi	Upper bound	0.0446	0.0531	0.0627	0.0738	0.0865	0.0998	0.1147	0.1321	0.1507	0.1708	0.1940	0.2168	0.2457	0.2723	0.2999	0.3304	0.3670	0.4021	0.4383	0.4742	0.5120	0.5497	0.5902	0.6279	0.6635	0.7021	0.7362	0.7728	0.8008	0.8232
Goa	Lower bound	0.0002	0.0009	0.0029	0.0084	0.0210	0.0387	0.0591	0.0745	0.0843	0.0883	0.0906	0.0914	0.0886	0.0843	0.0796	0.0732	0.0682	0.0619	0.0567	0.0526	0.0501	0.0482	0.0465	0.0453	0.0441	0.0427	0.0414	0.0401	0.0388	0.0377
Goa	Point	0.0020	0.0070	0.0218	0.0579	0.1040	0.1335	0.1473	0.1523	0.1535	0.1518	0.1476	0.1415	0.1330	0.1238	0.1143	0.1050	0.0969	0.0901	0.0839	0.0786	0.0744	0.0708	0.0676	0.0648	0.0624	0.0602	0.0582	0.0564	0.0548	0.0534
Goa	Upper bound	0.0054	0.0234	0.1178	0.1995	0.2143	0.2143	0.2124	0.2103	0.2053	0.1994	0.1946	0.1881	0.1759	0.1663	0.1571	0.1471	0.1376	0.1290	0.1227	0.1170	0.1103	0.1077	0.1027	0.0998	0.0968	0.0938	0.0911	0.0896	0.0897	0.0887
Gujarat	Lower bound	0.0055	0.0086	0.0134	0.0212	0.0337	0.0527	0.0817	0.1244	0.1756	0.2422	0.3163	0.4039	0.4893	0.5769	0.6545	0.7261	0.7863	0.8295	0.8651	0.8885	0.9005	0.9069	0.9150	0.9218	0.9308	0.9399	0.9490	0.9582	0.9663	0.9743
Gujarat	Point	0.0086	0.0136	0.0216	0.0346	0.0558	0.0880	0.1349	0.1998	0.2788	0.3730	0.4764	0.5836	0.6847	0.7758	0.8447	0.8960	0.9263	0.9433	0.9565	0.9679	0.9806	0.9932	1.0055	1.0172	1.0279	1.0395	1.0506	1.0618	1.0720	1.0813
Gujarat	Upper bound	0.0155	0.0257	0.0406	0.0642	0.1016	0.1605	0.2497	0.3628	0.4931	0.6250	0.7612	0.8783	0.9641	1.0320	1.0550	1.0706	1.0680	1.0811	1.0854	1.0934	1.1027	1.1177	1.1330	1.1494	1.1670	1.1857	1.2023	1.2114	1.2293	1.2482
Himachal Pradesh	Lower bound	0.0100	0.0116	0.0132	0.0151	0.0173	0.0197	0.0221	0.0249	0.0274	0.0301	0.0332	0.0355	0.0378	0.0398	0.0414	0.0427	0.0443	0.0464	0.0488	0.0515	0.0542	0.0568	0.0571	0.0575	0.0581	0.0584	0.0585	0.0588	0.0588	0.0591
Himachal Pradesh	Point	0.0159	0.0182	0.0208	0.0233	0.0262	0.0290	0.0319	0.0349	0.0378	0.0408	0.0438	0.0465	0.0491	0.0513	0.0534	0.0552	0.0570	0.0596	0.0626	0.0659	0.0692	0.0725	0.0726	0.0727	0.0728	0.0730	0.0732	0.0737	0.0741	0.0744
Himachal Pradesh	Upper bound	0.0233	0.0262	0.0292	0.0321	0.0356	0.0393	0.0427	0.0455	0.0489	0.0519	0.0548	0.0575	0.0599	0.0621	0.0638	0.0657	0.0678	0.0710	0.0745	0.0785	0.0827	0.0868	0.0882	0.0893	0.0909	0.0919	0.0921	0.0923	0.0927	0.0929
Haryana	Lower bound	0.0016	0.0026	0.0040	0.0060	0.0093	0.0148	0.0219	0.0321	0.0467	0.0666	0.0921	0.1241	0.1628	0.2041	0.2481	0.2890	0.3256	0.3621	0.3887	0.4038	0.4092	0.4042	0.3983	0.3949	0.3927	0.3907	0.3872	0.3889	0.3900	0.3896

Haryana	Point	0.0038	0.0060	0.0095	0.0149	0.0232	0.0358	0.0546	0.0810	0.1172	0.1618	0.2146	0.2696	0.3221	0.3665	0.4019	0.4259	0.4422	0.4501	0.4552	0.4577	0.4581	0.4568	0.4545	0.4531	0.4536	0.4552	0.4554	0.4593	0.4645	0.4681
Haryana	Upper bound	0.0066	0.0107	0.0177	0.0286	0.0456	0.0726	0.1129	0.1694	0.2387	0.3114	0.3761	0.4399	0.4869	0.5222	0.5385	0.5487	0.5571	0.5581	0.5619	0.5571	0.5520	0.5446	0.5402	0.5375	0.5389	0.5454	0.5427	0.5519	0.5658	0.5768
Jharkhand	Lower bound	0.0030	0.0046	0.0065	0.0088	0.0114	0.0137	0.0164	0.0194	0.0228	0.0269	0.0314	0.0363	0.0415	0.0470	0.0522	0.0584	0.0649	0.0716	0.0790	0.0877	0.0953	0.1039	0.1119	0.1211	0.1279	0.1354	0.1414	0.1472	0.1515	0.1551
Jharkhand	Point	0.0047	0.0068	0.0094	0.0125	0.0161	0.0192	0.0230	0.0273	0.0325	0.0386	0.0454	0.0529	0.0613	0.0702	0.0791	0.0886	0.0985	0.1091	0.1205	0.1325	0.1447	0.1572	0.1696	0.1815	0.1927	0.2030	0.2127	0.2216	0.2294	0.2365
Jharkhand	Upper bound	0.0055	0.0080	0.0110	0.0147	0.0195	0.0239	0.0292	0.0355	0.0431	0.0519	0.0618	0.0724	0.0840	0.0969	0.1095	0.1238	0.1387	0.1551	0.1710	0.1885	0.2065	0.2255	0.2443	0.2621	0.2786	0.2957	0.3100	0.3240	0.3384	0.3499
Jammu & Kashmir	Lower bound	0.0020	0.0026	0.0034	0.0042	0.0050	0.0059	0.0066	0.0073	0.0079	0.0087	0.0095	0.0104	0.0115	0.0128	0.0140	0.0154	0.0170	0.0191	0.0211	0.0236	0.0259	0.0285	0.0294	0.0300	0.0306	0.0313	0.0321	0.0330	0.0337	0.0341
Jammu & Kashmir	Point	0.0037	0.0049	0.0063	0.0077	0.0092	0.0109	0.0120	0.0132	0.0144	0.0158	0.0174	0.0190	0.0208	0.0229	0.0250	0.0272	0.0297	0.0324	0.0354	0.0388	0.0424	0.0460	0.0492	0.0523	0.0550	0.0576	0.0600	0.0621	0.0639	0.0653
Jammu & Kashmir	Upper bound	0.0047	0.0061	0.0079	0.0099	0.0123	0.0151	0.0175	0.0201	0.0227	0.0254	0.0283	0.0311	0.0342	0.0380	0.0413	0.0454	0.0495	0.0533	0.0571	0.0622	0.0676	0.0726	0.0765	0.0810	0.0849	0.0902	0.0939	0.0996	0.1052	0.1131
Karnataka	Lower bound	0.0525	0.1050	0.2406	0.5149	0.9144	1.3887	1.9706	2.7335	3.5894	4.3936	4.9978	5.3796	5.5140	5.4259	5.1299	4.7901	4.3968	4.0311	3.6875	3.4378	3.2126	3.0284	2.8794	2.7290	2.6112	2.5127	2.4280	2.3503	2.2845	2.2215
Karnataka	Point	0.1665	0.3927	0.6392	1.0211	1.4949	2.1956	3.1159	4.1623	5.1800	5.9704	6.4819	6.7094	6.7174	6.5550	6.2868	5.9476	5.5633	5.1706	4.7886	4.4507	4.1550	3.9024	3.6813	3.4902	3.3188	3.1653	3.0300	2.9122	2.8120	2.7370
Karnataka	Upper bound	0.5189	0.6740	1.1660	2.1437	3.3572	4.7314	5.9506	7.0085	7.5508	7.8257	8.0202	8.0786	8.0993	7.9549	7.6352	7.3314	6.8724	6.4593	6.0583	5.6569	5.2835	4.9596	4.6849	4.4151	4.1872	3.9922	3.8040	3.6568	3.5471	3.4403
Kerala	Lower bound	0.0013	0.0022	0.0038	0.0064	0.0107	0.0175	0.0282	0.0439	0.0642	0.0898	0.1178	0.1494	0.1779	0.2008	0.2203	0.2317	0.2400	0.2437	0.2434	0.2422	0.2415	0.2411	0.2404	0.2390	0.2377	0.2351	0.2341	0.2331	0.2317	0.2303
Kerala	Point	0.0031	0.0052	0.0087	0.0144	0.0237	0.0383	0.0598	0.0911	0.1314	0.1746	0.2163	0.2521	0.2750	0.2880	0.2932	0.2927	0.2916	0.2901	0.2877	0.2850	0.2827	0.2806	0.2786	0.2773	0.2762	0.2754	0.2749	0.2749	0.2752	0.2764
Kerala	Upper bound	0.0102	0.0188	0.0327	0.0554	0.0927	0.1387	0.2062	0.2708	0.3222	0.3578	0.3756	0.3850	0.3995	0.4102	0.4108	0.4080	0.4020	0.3939	0.3900	0.3807	0.3746	0.3681	0.3636	0.3609	0.3591	0.3597	0.3567	0.3588	0.3578	0.3632
Meghalaya	Lower bound	0.0000	0.0001	0.0003	0.0006	0.0009	0.0012	0.0016	0.0022	0.0029	0.0039	0.0049	0.0059	0.0069	0.0081	0.0101	0.0128	0.0165	0.0214	0.0276	0.0359	0.0456	0.0565	0.0685	0.0802	0.0903	0.0966	0.1008	0.1044	0.1071	0.1095

Meghalaya	Point	0.0011	0.0018	0.0025	0.0033	0.0037	0.0042	0.0047	0.0051	0.0055	0.0060	0.0065	0.0072	0.0081	0.0094	0.0117	0.0150	0.0198	0.0265	0.0353	0.0462	0.0588	0.0719	0.0845	0.0956	0.1048	0.1122	0.1180	0.1227	0.1265	0.1298
Meghalaya	Upper bound	0.0027	0.0029	0.0035	0.0043	0.0048	0.0053	0.0058	0.0062	0.0067	0.0071	0.0077	0.0083	0.0092	0.0107	0.0132	0.0172	0.0237	0.0328	0.0442	0.0579	0.0731	0.0866	0.0984	0.1085	0.1171	0.1249	0.1314	0.1381	0.1447	0.1505
Maharashtra	Lower bound	0.7694	1.7261	3.8397	5.9721	7.3511	8.0573	8.2787	8.0813	7.8683	7.4233	6.9188	6.4221	5.9198	5.3957	4.8782	4.4251	4.0350	3.6939	3.4663	3.2642	3.1283	3.0344	2.9503	2.8853	2.8260	2.7683	2.7195	2.6791	2.6350	2.6001
Maharashtra	Point	8.4695	9.5722	10.3927	10.9580	11.2616	11.3454	11.2434	10.9833	10.5947	10.1068	9.5464	8.9389	8.3035	7.6600	7.0259	6.4236	5.8706	5.3871	4.9932	4.6869	4.4455	4.2545	4.0919	3.9695	3.8845	3.8052	3.7324	3.6704	3.6141	3.5708
Maharashtra	Upper bound	12.0606	13.1031	13.7991	14.1419	14.5172	14.6516	14.4505	14.2397	13.9512	13.3205	12.5626	11.9831	11.3123	10.5955	9.7410	8.9879	8.2761	7.6948	7.1682	6.7598	6.3763	6.0791	5.8441	5.6566	5.5387	5.4584	5.3058	5.2633	5.2105	5.1984
Manipur	Lower bound	0.0002	0.0011	0.0038	0.0126	0.0376	0.0897	0.1718	0.2723	0.3531	0.4115	0.4416	0.4462	0.4422	0.4347	0.4241	0.4077	0.3951	0.3877	0.3792	0.3748	0.3699	0.3617	0.3536	0.3451	0.3372	0.3286	0.3181	0.3106	0.3038	0.2977
Manipur	Point	0.0091	0.0268	0.0665	0.1271	0.2059	0.2957	0.3856	0.4610	0.5121	0.5432	0.5542	0.5523	0.5441	0.5304	0.5124	0.4935	0.4768	0.4623	0.4499	0.4386	0.4269	0.4165	0.4063	0.3973	0.3897	0.3816	0.3749	0.3688	0.3645	0.3611
Manipur	Upper bound	0.1184	0.1402	0.2150	0.3576	0.5038	0.5833	0.6096	0.6430	0.6628	0.6820	0.6800	0.6631	0.6468	0.6271	0.6029	0.5799	0.5612	0.5455	0.5260	0.5098	0.4974	0.4831	0.4717	0.4641	0.4605	0.4556	0.4508	0.4464	0.4455	0.4449
Madhya Pradesh	Lower bound	0.0015	0.0036	0.0070	0.0120	0.0203	0.0352	0.0584	0.0886	0.1303	0.1868	0.2449	0.3105	0.3698	0.4181	0.4617	0.4918	0.5096	0.5226	0.5258	0.5263	0.5217	0.5152	0.5064	0.4974	0.4915	0.4850	0.4812	0.4775	0.4754	0.4712
Madhya Pradesh	Point	0.0046	0.0089	0.0149	0.0233	0.0368	0.0581	0.0910	0.1378	0.2022	0.2885	0.3776	0.4644	0.5343	0.5868	0.6241	0.6441	0.6509	0.6485	0.6426	0.6339	0.6243	0.6155	0.6079	0.6015	0.5970	0.5942	0.5937	0.5951	0.5975	0.6029
Madhya Pradesh	Upper bound	0.0207	0.0290	0.0366	0.0450	0.0596	0.0878	0.1404	0.2271	0.3426	0.4820	0.6090	0.7195	0.7865	0.8303	0.8593	0.8663	0.8559	0.8478	0.8307	0.8155	0.7944	0.7866	0.7767	0.7729	0.7739	0.7766	0.7823	0.7933	0.8012	0.8144
Mizoram	Lower bound	0.0001	0.0001	0.0003	0.0006	0.0011	0.0017	0.0032	0.0070	0.0144	0.0267	0.0427	0.0589	0.0708	0.0779	0.0835	0.0889	0.0948	0.1011	0.1051	0.1104	0.1170	0.1236	0.1312	0.1377	0.1460	0.1542	0.1610	0.1676	0.1745	0.1788
Mizoram	Point	0.0014	0.0028	0.0060	0.0124	0.0196	0.0275	0.0364	0.0470	0.0592	0.0707	0.0804	0.0895	0.0977	0.1052	0.1120	0.1184	0.1245	0.1295	0.1329	0.1375	0.1437	0.1509	0.1584	0.1664	0.1752	0.1844	0.1940	0.2038	0.2133	0.2224
Mizoram	Upper bound	0.1227	0.1213	0.1279	0.1270	0.1059	0.0783	0.0692	0.0803	0.0935	0.1041	0.1138	0.1231	0.1313	0.1399	0.1468	0.1521	0.1584	0.1632	0.1649	0.1682	0.1756	0.1831	0.1925	0.2036	0.2143	0.2251	0.2376	0.2484	0.2608	0.2731
Nagaland	Lower bound	0.0001	0.0002	0.0006	0.0015	0.0036	0.0083	0.0184	0.0413	0.0664	0.0962	0.1233	0.1395	0.1453	0.1521	0.1567	0.1562	0.1574	0.1601	0.1637	0.1677	0.1727	0.1797	0.1858	0.1931	0.2003	0.2075	0.2136	0.2205	0.2277	0.2342

Nagaland	Point	0.0025	0.0064	0.0164	0.0311	0.0514	0.0760	0.1015	0.1239	0.1401	0.1540	0.1662	0.1773	0.1867	0.1946	0.1990	0.2000	0.2012	0.2035	0.2066	0.2110	0.2178	0.2256	0.2338	0.2423	0.2511	0.2596	0.2672	0.2755	0.2841	0.2926
Nagaland	Upper bound	0.2148	0.2010	0.1937	0.1759	0.1464	0.1374	0.1526	0.1689	0.1833	0.1987	0.2122	0.2254	0.2354	0.2421	0.2481	0.2476	0.2483	0.2488	0.2529	0.2580	0.2651	0.2752	0.2847	0.2947	0.3047	0.3145	0.3239	0.3343	0.3449	0.3556
Odisha	Lower bound	0.0012	0.0022	0.0040	0.0063	0.0103	0.0157	0.0231	0.0342	0.0496	0.0694	0.0957	0.1272	0.1666	0.2133	0.2623	0.3101	0.3529	0.3868	0.4172	0.4393	0.4504	0.4564	0.4562	0.4559	0.4533	0.4509	0.4493	0.4491	0.4481	0.4478
Odisha	Point	0.0032	0.0053	0.0086	0.0125	0.0183	0.0270	0.0394	0.0571	0.0812	0.1140	0.1561	0.2071	0.2642	0.3234	0.3797	0.4285	0.4671	0.4927	0.5109	0.5218	0.5266	0.5272	0.5252	0.5229	0.5204	0.5181	0.5163	0.5150	0.5142	0.5141
Odisha	Upper bound	0.0074	0.0112	0.0162	0.0224	0.0326	0.0495	0.0740	0.1092	0.1550	0.2119	0.2772	0.3572	0.4385	0.5138	0.5709	0.6110	0.6363	0.6412	0.6439	0.6537	0.6532	0.6487	0.6446	0.6416	0.6398	0.6354	0.6291	0.6260	0.6225	0.6194
Punjab	Lower bound	0.0045	0.0071	0.0111	0.0177	0.0282	0.0434	0.0664	0.0984	0.1430	0.2021	0.2740	0.3576	0.4434	0.5260	0.5993	0.6506	0.6771	0.6840	0.6706	0.6553	0.6390	0.6250	0.6066	0.6007	0.5963	0.5916	0.5913	0.5834	0.5864	0.5898
Punjab	Point	0.0096	0.0155	0.0251	0.0406	0.0645	0.1008	0.1531	0.2252	0.3146	0.4167	0.5217	0.6181	0.6954	0.7493	0.7830	0.7967	0.7956	0.7877	0.7765	0.7652	0.7538	0.7426	0.7302	0.7207	0.7141	0.7104	0.7081	0.7088	0.7123	0.7168
Punjab	Upper bound	0.0374	0.0631	0.1043	0.1855	0.2906	0.4263	0.6072	0.7355	0.8636	0.9396	0.9743	1.0067	1.0170	1.0095	1.0090	1.0139	1.0033	0.9881	0.9582	0.9454	0.9350	0.9224	0.9080	0.8959	0.8881	0.8847	0.8953	0.9064	0.9039	0.9201
Rajasthan	Lower bound	0.0110	0.0167	0.0236	0.0319	0.0402	0.0490	0.0579	0.0685	0.0812	0.0957	0.1113	0.1291	0.1468	0.1663	0.1853	0.2060	0.2290	0.2526	0.2784	0.3051	0.3322	0.3592	0.3851	0.4091	0.4316	0.4514	0.4696	0.4839	0.4969	0.5038
Rajasthan	Point	0.0143	0.0215	0.0302	0.0406	0.0512	0.0623	0.0754	0.0906	0.1079	0.1266	0.1470	0.1689	0.1921	0.2157	0.2399	0.2658	0.2943	0.3251	0.3582	0.3931	0.4283	0.4634	0.4974	0.5296	0.5596	0.5864	0.6107	0.6323	0.6513	0.6667
Rajasthan	Upper bound	0.0175	0.0260	0.0361	0.0485	0.0618	0.0757	0.0913	0.1094	0.1297	0.1525	0.1764	0.2018	0.2290	0.2582	0.2856	0.3177	0.3519	0.3881	0.4264	0.4675	0.5085	0.5485	0.5917	0.6305	0.6662	0.6989	0.7299	0.7622	0.7894	0.8088
Sikkim	Lower bound	0.0001	0.0001	0.0001	0.0002	0.0002	0.0002	0.0003	0.0003	0.0004	0.0004	0.0005	0.0005	0.0006	0.0006	0.0007	0.0008	0.0009	0.0010	0.0011	0.0013	0.0014	0.0016	0.0016	0.0017	0.0018	0.0018	0.0019	0.0020	0.0020	0.0021
Sikkim	Point	0.0002	0.0003	0.0003	0.0004	0.0004	0.0005	0.0006	0.0006	0.0007	0.0007	0.0008	0.0009	0.0010	0.0011	0.0012	0.0013	0.0014	0.0016	0.0018	0.0021	0.0023	0.0026	0.0027	0.0028	0.0030	0.0031	0.0033	0.0035	0.0037	0.0038
Sikkim	Upper bound	0.0004	0.0006	0.0007	0.0009	0.0010	0.0012	0.0014	0.0015	0.0017	0.0019	0.0020	0.0022	0.0024	0.0025	0.0028	0.0030	0.0032	0.0035	0.0037	0.0039	0.0041	0.0043	0.0047	0.0051	0.0055	0.0059	0.0065	0.0070	0.0077	0.0084
Tamil Nadu	Lower bound	0.0169	0.0433	0.0810	0.1329	0.2309	0.4701	0.9591	1.6585	2.3022	2.6260	2.6539	2.5255	2.3168	2.1242	1.9553	1.8106	1.7253	1.6296	1.5687	1.5245	1.4743	1.4393	1.4135	1.3859	1.3509	1.3289	1.3101	1.2846	1.2644	1.2355

Tamil Nadu	Point	0.1555	0.3054	0.5977	1.0755	1.6939	2.3346	2.8847	3.1988	3.3373	3.3734	3.3451	3.2686	3.1246	2.9508	2.7631	2.5793	2.4280	2.2889	2.1741	2.0789	1.9999	1.9352	1.8801	1.8320	1.7930	1.7602	1.7321	1.7082	1.6880	1.6701
Tamil Nadu	Upper bound	3.6374	4.2605	4.9315	5.0205	4.7093	4.4983	4.4564	4.4928	4.4634	4.4177	4.3447	4.2242	4.0592	3.8456	3.6577	3.4748	3.2631	3.1226	2.9509	2.7745	2.6425	2.5257	2.4365	2.3520	2.2766	2.2122	2.1757	2.1443	2.1372	2.1371
Tripura	Lower bound	0.0001	0.0001	0.0001	0.0002	0.0002	0.0002	0.0003	0.0004	0.0005	0.0006	0.0007	0.0009	0.0010	0.0013	0.0015	0.0019	0.0022	0.0027	0.0033	0.0040	0.0049	0.0059	0.0071	0.0085	0.0100	0.0117	0.0137	0.0159	0.0184	0.0210
Tripura	Point	0.0001	0.0002	0.0002	0.0003	0.0003	0.0004	0.0005	0.0006	0.0007	0.0008	0.0010	0.0012	0.0014	0.0017	0.0020	0.0024	0.0029	0.0035	0.0042	0.0052	0.0063	0.0076	0.0092	0.0110	0.0132	0.0156	0.0185	0.0217	0.0254	0.0296
Tripura	Upper bound	0.0004	0.0007	0.0010	0.0011	0.0011	0.0012	0.0013	0.0013	0.0015	0.0016	0.0018	0.0021	0.0023	0.0026	0.0029	0.0032	0.0037	0.0044	0.0052	0.0062	0.0076	0.0092	0.0112	0.0137	0.0164	0.0197	0.0236	0.0281	0.0332	0.0389
Uttarakhand	Lower bound	0.0065	0.0077	0.0091	0.0107	0.0125	0.0145	0.0166	0.0191	0.0220	0.0253	0.0290	0.0331	0.0373	0.0415	0.0463	0.0505	0.0554	0.0604	0.0660	0.0713	0.0757	0.0796	0.0834	0.0860	0.0882	0.0904	0.0919	0.0930	0.0934	0.0935
Uttarakhand	Point	0.0135	0.0158	0.0186	0.0216	0.0252	0.0290	0.0331	0.0377	0.0424	0.0473	0.0522	0.0574	0.0622	0.0671	0.0716	0.0759	0.0800	0.0845	0.0888	0.0933	0.0978	0.1022	0.1062	0.1097	0.1128	0.1153	0.1173	0.1188	0.1198	0.1205
Uttarakhand	Upper bound	0.0227	0.0264	0.0310	0.0356	0.0406	0.0456	0.0507	0.0561	0.0620	0.0679	0.0733	0.0793	0.0846	0.0895	0.0945	0.1001	0.1048	0.1091	0.1140	0.1202	0.1244	0.1295	0.1347	0.1392	0.1436	0.1472	0.1497	0.1522	0.1556	0.1562
Uttar Pradesh	Lower bound	0.1595	0.1899	0.2214	0.2599	0.3022	0.3510	0.4003	0.4508	0.5040	0.5630	0.6146	0.6672	0.7228	0.7671	0.8071	0.8468	0.8892	0.9185	0.9607	0.9973	1.0412	1.0810	1.1171	1.1533	1.1799	1.2022	1.2259	1.2448	1.2613	1.2746
Uttar Pradesh	Point	0.2430	0.2850	0.3328	0.3872	0.4461	0.5110	0.5788	0.6506	0.7224	0.7967	0.8688	0.9373	1.0077	1.0710	1.1234	1.1756	1.2285	1.2789	1.3289	1.3814	1.4320	1.4800	1.5235	1.5614	1.5944	1.6225	1.6460	1.6660	1.6820	1.6949
Uttar Pradesh	Upper bound	0.3475	0.4099	0.4797	0.5527	0.6322	0.7198	0.7980	0.8848	0.9746	1.0765	1.1632	1.2554	1.3401	1.4180	1.4894	1.5683	1.6375	1.6887	1.7582	1.8370	1.9092	1.9769	2.0334	2.0827	2.1224	2.1561	2.1843	2.2103	2.2349	2.2545
West Bengal	Lower bound	0.0042	0.0174	0.0717	0.2307	0.5040	0.7433	0.8521	0.8924	0.9020	0.8979	0.8820	0.8528	0.8158	0.7742	0.7313	0.6911	0.6546	0.6252	0.5969	0.5749	0.5649	0.5588	0.5541	0.5495	0.5500	0.5500	0.5521	0.5578	0.5627	0.5676
West Bengal	Point	0.0081	0.0282	0.0940	0.2730	0.5804	0.8369	0.9539	0.9976	1.0100	1.0054	0.9871	0.9594	0.9267	0.8924	0.8580	0.8246	0.7934	0.7666	0.7424	0.7230	0.7118	0.7072	0.7069	0.7107	0.7179	0.7289	0.7432	0.7606	0.7791	0.8000
West Bengal	Upper bound	0.0151	0.0645	0.1744	0.5505	1.0277	1.2248	1.3003	1.3284	1.3259	1.3007	1.2698	1.2119	1.1694	1.1226	1.0783	1.0416	1.0083	0.9853	0.9682	0.9505	0.9373	0.9371	0.9437	0.9554	0.9720	1.0017	1.0227	1.0543	1.0833	1.1170
Andaman & Nicobar	Lower bound	0.0003	0.0004	0.0005	0.0006	0.0006	0.0007	0.0007	0.0008	0.0008	0.0009	0.0009	0.0010	0.0010	0.0011	0.0011	0.0012	0.0013	0.0014	0.0014	0.0014	0.0015	0.0015	0.0016	0.0016	0.0017	0.0017	0.0018	0.0018	0.0019	0.0020

Andaman & Nicobar	Point	0.0006	0.0007	0.0009	0.0010	0.0012	0.0013	0.0014	0.0015	0.0016	0.0017	0.0019	0.0020	0.0022	0.0023	0.0025	0.0027	0.0028	0.0030	0.0032	0.0034	0.0036	0.0038	0.0040	0.0042	0.0044	0.0046	0.0048	0.0049	0.0049	0.0049
Andaman & Nicobar	Upper bound	0.0010	0.0012	0.0015	0.0017	0.0021	0.0024	0.0027	0.0029	0.0033	0.0036	0.0039	0.0043	0.0046	0.0049	0.0052	0.0055	0.0060	0.0063	0.0067	0.0070	0.0073	0.0079	0.0085	0.0089	0.0095	0.0101	0.0110	0.0115	0.0122	0.0129
Chandigarh	Lower bound	0.0001	0.0002	0.0004	0.0006	0.0011	0.0018	0.0028	0.0043	0.0062	0.0086	0.0111	0.0135	0.0159	0.0175	0.0185	0.0189	0.0191	0.0192	0.0191	0.0187	0.0180	0.0173	0.0165	0.0160	0.0153	0.0149	0.0144	0.0141	0.0139	0.0136
Chandigarh	Point	0.0004	0.0007	0.0013	0.0023	0.0039	0.0064	0.0097	0.0133	0.0168	0.0197	0.0214	0.0226	0.0233	0.0235	0.0235	0.0233	0.0232	0.0231	0.0229	0.0225	0.0221	0.0217	0.0213	0.0211	0.0209	0.0208	0.0207	0.0207	0.0208	0.0210
Chandigarh	Upper bound	0.0008	0.0025	0.0086	0.0212	0.0347	0.0427	0.0477	0.0499	0.0484	0.0470	0.0443	0.0420	0.0395	0.0362	0.0340	0.0319	0.0307	0.0289	0.0279	0.0273	0.0268	0.0263	0.0259	0.0261	0.0263	0.0265	0.0268	0.0272	0.0278	0.0284
Dadra & Nagar	Lower bound	0.0000	0.0001	0.0001	0.0001	0.0001	0.0001	0.0002	0.0002	0.0002	0.0003	0.0003	0.0004	0.0004	0.0005	0.0006	0.0007	0.0008	0.0009	0.0010	0.0012	0.0014	0.0016	0.0019	0.0022	0.0025	0.0029	0.0033	0.0037	0.0040	0.0043
Dadra & Nagar	Point	0.0001	0.0002	0.0002	0.0002	0.0002	0.0003	0.0003	0.0004	0.0004	0.0005	0.0005	0.0006	0.0007	0.0008	0.0010	0.0011	0.0013	0.0015	0.0017	0.0020	0.0023	0.0027	0.0031	0.0035	0.0040	0.0045	0.0051	0.0056	0.0062	0.0069
Dadra & Nagar	Upper bound	0.0002	0.0003	0.0004	0.0005	0.0006	0.0007	0.0008	0.0009	0.0010	0.0012	0.0013	0.0015	0.0017	0.0019	0.0021	0.0024	0.0027	0.0029	0.0032	0.0036	0.0041	0.0045	0.0049	0.0055	0.0061	0.0070	0.0077	0.0087	0.0097	0.0109
Daman & Diu	Lower bound	0.0000	0.0000	0.0000	0.0000	0.0000	0.0001	0.0001	0.0002	0.0003	0.0005	0.0007	0.0010	0.0014	0.0017	0.0020	0.0022	0.0022	0.0022	0.0021	0.0021	0.0021	0.0021	0.0021	0.0022	0.0022	0.0023	0.0024	0.0025	0.0025	0.0026
Daman & Diu	Point	0.0000	0.0000	0.0001	0.0001	0.0003	0.0004	0.0007	0.0011	0.0016	0.0020	0.0024	0.0027	0.0029	0.0030	0.0030	0.0030	0.0030	0.0029	0.0029	0.0028	0.0028	0.0028	0.0029	0.0029	0.0030	0.0031	0.0032	0.0034	0.0035	0.0037
Daman & Diu	Upper bound	0.0004	0.0007	0.0013	0.0021	0.0028	0.0033	0.0038	0.0041	0.0044	0.0043	0.0044	0.0044	0.0044	0.0043	0.0042	0.0042	0.0041	0.0039	0.0038	0.0038	0.0037	0.0037	0.0038	0.0040	0.0041	0.0042	0.0044	0.0047	0.0049	0.0052
Pondicherry	Lower bound	0.0015	0.0021	0.0028	0.0035	0.0042	0.0049	0.0056	0.0061	0.0066	0.0072	0.0079	0.0085	0.0092	0.0098	0.0103	0.0109	0.0119	0.0131	0.0145	0.0160	0.0177	0.0195	0.0201	0.0207	0.0212	0.0216	0.0220	0.0222	0.0222	0.0219
Pondicherry	Point	0.0024	0.0033	0.0043	0.0053	0.0063	0.0072	0.0081	0.0087	0.0094	0.0101	0.0110	0.0119	0.0129	0.0139	0.0152	0.0168	0.0189	0.0214	0.0241	0.0272	0.0307	0.0345	0.0355	0.0363	0.0369	0.0373	0.0375	0.0375	0.0374	0.0370
Pondicherry	Upper bound	0.0139	0.0168	0.0195	0.0206	0.0217	0.0215	0.0212	0.0200	0.0187	0.0178	0.0170	0.0169	0.0177	0.0188	0.0211	0.0235	0.0274	0.0316	0.0363	0.0413	0.0473	0.0544	0.0564	0.0579	0.0587	0.0593	0.0597	0.0599	0.0606	0.0609
Telangana	Lower bound	0.0839	0.1679	0.3092	0.5215	0.7978	1.1605	1.5726	1.9873	2.3039	2.5159	2.5950	2.5820	2.5210	2.3859	2.2305	2.0870	1.9395	1.7878	1.6829	1.5704	1.5071	1.4271	1.3788	1.3124	1.2710	1.2173	1.1828	1.1457	1.1104	1.0797

Telangana	Point	0.2296	0.3463	0.5028	0.7493	1.1118	1.5656	2.0480	2.4942	2.8257	3.0379	3.1378	3.1392	3.0901	2.9903	2.8609	2.7116	2.5523	2.3955	2.2581	2.1387	2.0336	1.9382	1.8532	1.7756	1.7117	1.6532	1.6000	1.5502	1.5051	1.4796
Telangana	Upper bound	0.6474	1.2337	2.0626	2.9654	3.5393	3.8244	3.8937	3.9469	3.9805	3.9098	3.8535	3.7925	3.7462	3.6647	3.5653	3.4430	3.2749	3.0965	2.9204	2.7718	2.6533	2.5310	2.4208	2.3312	2.2360	2.1744	2.1273	2.0754	2.0196	2.0028
