# Supplementary material for: Diversity in HIV epidemic transitions in India: An application of HIV epidemiological metrices and benchmarks
Source: PLoS One. 2022 Jul 18;17(7):e0270886. doi: 10.1371/journal.pone.0270886 (PMC9292090; doi:10.1371/journal.pone.0270886)
Supplement: S2 Table — (PDF) [file pone.0270886.s002.pdf]

S2 Table. IPR and IMR by States/UT in India, 1990-2019

Table a. IPR by States/UTs in India, 1990-2019

State/UT	Configuration	1990	1991	1992	1993	1994	1995	1996	1997	1998	1999	2000	2001	2002	2003	2004	2005	2006	2007	2008	2009	2010	2011	2012	2013	2014	2015	2016	2017	2018	2019
Andhra Pradesh	Upper bound	0.850	0.755	0.661	0.593	0.543	0.552	0.529	0.478	0.408	0.283	0.186	0.117	0.084	0.058	0.044	0.035	0.030	0.027	0.025	0.023	0.023	0.022	0.021	0.020	0.019	0.018	0.018	0.018	0.017	0.017
Andhra Pradesh	Point	0.583	0.556	0.521	0.449	0.397	0.394	0.373	0.322	0.267	0.183	0.125	0.078	0.056	0.040	0.033	0.028	0.025	0.022	0.019	0.018	0.016	0.015	0.014	0.013	0.012	0.011	0.011	0.010	0.009	0.009
Andhra Pradesh	Lower bound	0.008	0.007	0.006	0.006	0.107	0.298	0.272	0.214	0.151	0.095	0.069	0.048	0.038	0.029	0.024	0.021	0.019	0.016	0.013	0.012	0.010	0.009	0.008	0.007	0.006	0.006	0.005	0.005	0.004	0.004
Arunachal Pradesh	Upper bound	1.920	0.360	0.319	0.286	0.271	0.259	0.233	0.206	0.205	0.218	0.214	0.210	0.206	0.197	0.192	0.193	0.179	0.170	0.162	0.161	0.156	0.153	0.151	0.147	0.145	0.140	0.135	0.134	0.129	0.122
Arunachal Pradesh	Point	0.351	0.299	0.263	0.234	0.216	0.199	0.173	0.156	0.164	0.177	0.176	0.172	0.174	0.167	0.166	0.168	0.159	0.150	0.141	0.139	0.134	0.132	0.130	0.125	0.122	0.118	0.112	0.111	0.106	0.100
Arunachal Pradesh	Lower bound	0.289	0.247	0.215	0.197	0.179	0.166	0.146	0.131	0.136	0.145	0.143	0.141	0.142	0.137	0.137	0.140	0.132	0.125	0.116	0.113	0.107	0.103	0.099	0.093	0.089	0.084	0.076	0.074	0.068	0.061
Assam	Upper bound	1.410	0.279	0.262	0.233	0.195	0.191	0.196	0.196	0.194	0.195	0.192	0.185	0.180	0.174	0.171	0.163	0.156	0.152	0.144	0.138	0.132	0.125	0.120	0.113	0.105	0.098	0.092	0.086	0.080	0.073
Assam	Point	0.278	0.248	0.231	0.207	0.174	0.171	0.176	0.176	0.175	0.176	0.174	0.167	0.163	0.158	0.154	0.146	0.139	0.135	0.128	0.122	0.115	0.109	0.103	0.097	0.089	0.083	0.077	0.071	0.066	0.059
Assam	Lower bound	0.244	0.219	0.204	0.182	0.154	0.150	0.154	0.156	0.155	0.157	0.156	0.149	0.145	0.139	0.135	0.128	0.122	0.118	0.112	0.106	0.100	0.094	0.089	0.083	0.077	0.070	0.065	0.060	0.055	0.049
Bihar	Upper bound	2.342	0.488	0.416	0.366	0.327	0.260	0.249	0.248	0.242	0.239	0.234	0.227	0.221	0.211	0.206	0.198	0.191	0.182	0.172	0.163	0.152	0.144	0.135	0.124	0.112	0.103	0.094	0.086	0.079	0.070
Bihar	Point	0.572	0.420	0.354	0.316	0.289	0.230	0.222	0.223	0.222	0.220	0.217	0.210	0.202	0.193	0.188	0.180	0.173	0.166	0.157	0.149	0.138	0.130	0.121	0.110	0.099	0.090	0.081	0.073	0.065	0.057
Bihar	Lower bound	0.478	0.353	0.298	0.268	0.247	0.197	0.193	0.194	0.193	0.194	0.194	0.189	0.183	0.175	0.170	0.162	0.157	0.150	0.141	0.133	0.123	0.115	0.105	0.094	0.082	0.073	0.064	0.055	0.046	0.037
Chhattishgarh	Upper bound	0.782	0.655	0.606	0.596	0.568	0.513	0.416	0.318	0.237	0.161	0.109	0.077	0.063	0.060	0.060	0.062	0.065	0.069	0.073	0.080	0.081	0.084	0.086	0.086	0.085	0.083	0.083	0.081	0.080	0.079
Chhattishgarh	Point	0.585	0.511	0.506	0.503	0.488	0.444	0.367	0.278	0.199	0.131	0.088	0.065	0.055	0.053	0.053	0.054	0.056	0.058	0.061	0.067	0.067	0.070	0.072	0.072	0.072	0.070	0.071	0.069	0.068	0.068
Chhattishgarh	Lower bound	0.332	0.390	0.435	0.457	0.455	0.420	0.307	0.178	0.110	0.075	0.058	0.048	0.043	0.043	0.045	0.046	0.047	0.048	0.049	0.053	0.052	0.054	0.055	0.056	0.056	0.054	0.055	0.052	0.052	0.052
Delhi	Upper bound	0.228	0.227	0.229	0.228	0.223	0.223	0.219	0.219	0.215	0.208	0.203	0.196	0.192	0.183	0.174	0.166	0.155	0.147	0.138	0.131	0.123	0.116	0.109	0.102	0.095	0.087	0.077	0.075	0.066	0.057
Delhi	Point	0.207	0.207	0.209	0.208	0.202	0.200	0.194	0.191	0.188	0.183	0.179	0.173	0.168	0.160	0.153	0.148	0.139	0.132	0.123	0.116	0.108	0.101	0.095	0.088	0.081	0.074	0.064	0.062	0.053	0.045
Delhi	Lower bound	0.185	0.186	0.189	0.188	0.183	0.180	0.174	0.170	0.167	0.162	0.158	0.152	0.147	0.140	0.134	0.130	0.123	0.116	0.108	0.102	0.095	0.089	0.083	0.077	0.070	0.063	0.054	0.052	0.043	0.035
Goa	Upper bound	0.952	0.895	0.828	0.857	0.716	0.548	0.433	0.283	0.187	0.111	0.072	0.053	0.035	0.032	0.030	0.029	0.028	0.035	0.035	0.033	0.032	0.030	0.029	0.029	0.027	0.028	0.028	0.028	0.028	0.028
Goa	Point	0.771	0.728	0.687	0.636	0.457	0.240	0.119	0.066	0.051	0.042	0.036	0.032	0.021	0.019	0.017	0.015	0.013	0.016	0.015	0.014	0.013	0.013	0.012	0.012	0.011	0.011	0.011	0.011	0.011	0.010
Goa	Lower bound	0.493	0.474	0.471	0.420	0.099	0.049	0.041	0.035	0.032	0.027	0.021	0.017	0.010	0.009	0.007	0.006	0.005	0.006	0.006	0.006	0.005	0.005	0.004	0.004	0.004	0.004	0.004	0.004	0.003	0.003
Gujarat	Upper bound	0.432	0.431	0.437	0.446	0.465	0.467	0.436	0.397	0.355	0.326	0.290	0.259	0.226	0.201	0.168	0.144	0.116	0.098	0.082	0.069	0.060	0.055	0.052	0.049	0.045	0.045	0.043	0.042	0.040	0.038
Gujarat	Point	0.392	0.395	0.398	0.401	0.407	0.393	0.375	0.353	0.313	0.285	0.251	0.221	0.189	0.162	0.131	0.110	0.088	0.076	0.066	0.058	0.053	0.049	0.046	0.043	0.039	0.038	0.037	0.035	0.033	0.031
Gujarat	Lower bound	0.362	0.367	0.369	0.365	0.360	0.337	0.316	0.300	0.260	0.234	0.205	0.170	0.138	0.115	0.093	0.079	0.066	0.061	0.056	0.051	0.046	0.042	0.039	0.036	0.033	0.032	0.030	0.029	0.027	0.025
Himachal Pradesh	Upper bound	0.198	0.198	0.191	0.181	0.180	0.173	0.166	0.161	0.156	0.152	0.148	0.139	0.133	0.124	0.121	0.117	0.115	0.117	0.112	0.109	0.101	0.092	0.055	0.055	0.052	0.044	0.032	0.031	0.030	0.028
Himachal Pradesh	Point	0.176	0.174	0.170	0.162	0.160	0.154	0.147	0.142	0.138	0.134	0.130	0.122	0.118	0.109	0.107	0.103	0.102	0.104	0.100	0.097	0.090	0.080	0.034	0.034	0.034	0.032	0.027	0.025	0.024	0.022
Himachal Pradesh	Lower bound	0.152	0.153	0.149	0.142	0.141	0.135	0.128	0.124	0.120	0.116	0.113	0.106	0.102	0.094	0.093	0.089	0.089	0.093	0.090	0.087	0.080	0.071	0.030	0.030	0.029	0.026	0.023	0.021	0.019	0.018
Haryana	Upper bound	0.443	0.446	0.438	0.441	0.432	0.429	0.423	0.406	0.389	0.357	0.334	0.300	0.268	0.233	0.205	0.169	0.143	0.113	0.100	0.090	0.080	0.073	0.070	0.069	0.068	0.067	0.067	0.067	0.069	0.067
Haryana	Point	0.393	0.395	0.389	0.392	0.385	0.381	0.372	0.355	0.339	0.307	0.279	0.239	0.201	0.163	0.135	0.108	0.091	0.074	0.070	0.068	0.065	0.063	0.061	0.059	0.057	0.057	0.056	0.057	0.057	0.056
Haryana	Lower bound	0.349	0.356	0.352	0.356	0.353	0.354	0.352	0.337	0.308	0.258	0.216	0.171	0.134	0.105	0.089	0.075	0.068	0.060	0.060	0.060	0.057	0.055	0.052	0.050	0.047	0.046	0.045	0.045	0.045	0.043
Jharkhand	Upper bound	1.933	0.389	0.345	0.316	0.298	0.231	0.234	0.231	0.232	0.231	0.226	0.218	0.215	0.206	0.191	0.185	0.175	0.169	0.157	0.148	0.139	0.129	0.121	0.113	0.104	0.095	0.089	0.082	0.077	0.071
Jharkhand	Point	0.429	0.342	0.301	0.278	0.260	0.204	0.208	0.206	0.209	0.210	0.204	0.196	0.192	0.183	0.170	0.164	0.155	0.149	0.138	0.129	0.120	0.111	0.103	0.094	0.087	0.078	0.072	0.066	0.060	0.055
Jharkhand	Lower bound	0.359	0.288	0.254	0.235	0.222	0.175	0.180	0.178	0.184	0.184	0.180	0.174	0.170	0.162	0.149	0.145	0.137	0.132	0.121	0.112	0.103	0.094	0.084	0.077	0.070	0.061	0.055	0.050	0.044	0.040
Jammu & Kashmir	Upper bound	1.797	0.341	0.301	0.273	0.254	0.238	0.173	0.171	0.166	0.171	0.174	0.166	0.171	0.177	0.167	0.161	0.156	0.146	0.140	0.140	0.141	0.136	0.129	0.123	0.114	0.109	0.102	0.098	0.095	0.091
Jammu & Kashmir	Point	0.329	0.272	0.240	0.219	0.203	0.192	0.141	0.141	0.139	0.145	0.149	0.143	0.148	0.154	0.145	0.139	0.135	0.126	0.121	0.117	0.116	0.110	0.095	0.087	0.077	0.072	0.065	0.060	0.054	0.048
Jammu & Kashmir	Lower bound	0.260	0.220	0.194	0.173	0.162	0.156	0.115	0.116	0.116	0.123	0.128	0.123	0.129	0.134	0.125	0.120	0.116	0.107	0.102	0.098	0.096	0.089	0.049	0.050	0.051	0.048	0.039	0.035	0.031	0.028
Karnataka	Upper bound	1.154	1.027	0.778	0.627	0.466	0.430	0.380	0.329	0.287	0.236	0.183	0.133	0.095	0.066	0.049	0.039	0.032	0.028	0.026	0.024	0.022	0.021	0.019	0.017	0.016	0.015	0.014	0.013	0.012	0.011
Karnataka	Point	0.634	0.597	0.409	0.399	0.344	0.348	0.326	0.284	0.232	0.172	0.125	0.087	0.062	0.045	0.036	0.031	0.026	0.022	0.020	0.017	0.015	0.013	0.012	0.010	0.010	0.009	0.008	0.007	0.007	0.006
Karnataka	Lower bound	0.184	0.214	0.245	0.275	0.263	0.288	0.245	0.173	0.118	0.081	0.063	0.049	0.041	0.032	0.028	0.023	0.019	0.015	0.013	0.011	0.009	0.008	0.006	0.006	0.005	0.004	0.004	0.003	0.003	0.003
Kerala	Upper bound	0.502	0.510	0.497	0.485	0.488	0.475	0.455	0.445	0.409	0.349	0.292	0.243	0.182	0.142	0.113	0.089	0.072	0.058	0.053	0.052	0.054	0.051	0.051	0.049	0.048	0.046	0.045	0.044	0.044	0.043
Kerala	Point	0.414	0.422	0.425	0.416	0.416	0.405	0.383	0.368	0.332	0.276	0.224	0.177	0.124	0.093	0.073	0.059	0.051	0.046	0.042	0.040	0.037	0.036	0.034	0.033	0.032	0.032	0.031	0.030	0.030	0.029
Kerala	Lower bound	0.355	0.366	0.374	0.365	0.357	0.327	0.300	0.254	0.192	0.138	0.104	0.083	0.065	0.055	0.050	0.045	0.040	0.035	0.032	0.030	0.030	0.028	0.027	0.025	0.024	0.023	0.023	0.022	0.021	0.020
Meghalaya	Upper bound	#DIV/0!	1.820	1.248	0.965	0.533	0.425	0.346	0.291	0.252	0.228	0.213	0.203	0.200	0.231	0.280	0.306	0.319	0.315	0.303	0.285	0.260	0.234	0.207	0.181	0.153	0.130	0.108	0.092	0.079	0.067
Meghalaya	Point	0.591	0.405	0.303	0.248	0.160	0.149	0.139	0.133	0.130	0.130	0.135	0.148	0.165	0.200	0.241	0.262	0.284	0.289	0.283	0.268	0.243	0.212	0.180	0.150	0.122	0.101	0.084	0.073	0.064	0.057
Meghalaya	Lower bound	#DIV/0!	0.265	0.219	0.205	0.139	0.132	0.124	0.119	0.117	0.117	0.121	0.133	0.149	0.179	0.214	0.230	0.242	0.251	0.260	0.253	0.222	0.184	0.149	0.119	0.097	0.082	0.070	0.062	0.056	0.049
Maharashtra	Upper bound	1.724	1.332	0.925	0.605	0.248	0.133	0.093	0.078	0.072	0.065	0.061	0.057	0.053	0.050	0.048	0.047	0.046	0.045	0.044	0.044	0.044	0.043	0.044	0.043	0.042	0.043	0.044	0.044	0.043	0.040
Maharashtra	Point	0.203	0.151	0.122	0.102	0.086	0.074	0.065	0.058	0.052	0.047	0.043	0.040	0.037	0.035	0.034	0.033	0.032	0.032	0.031	0.031	0.031	0.031	0.031	0.030	0.028	0.028	0.028	0.028	0.027	0.024
Maharashtra	Lower bound	0.105	0.096	0.084	0.074	0.063	0.056	0.049	0.045	0.040	0.036	0.033	0.030	0.027	0.025	0.024	0.023	0.022	0.022	0.022	0.021	0.021	0.021	0.021	0.020	0.018	0.018	0.018	0.017	0.016	0.014
Manipur	Upper bound	1.186	1.236	1.025	0.958	0.868	0.703	0.554	0.409	0.284	0.185	0.106	0.073	0.063	0.057	0.052	0.048	0.045	0.045	0.047	0.048	0.045	0.044	0.044	0.043	0.042	0.043	0.042	0.042	0.041	0.040
Manipur	Point	0.678	0.679	0.616	0.496	0.405	0.328	0.261	0.195	0.137	0.101	0.070	0.055	0.050	0.046	0.043	0.039	0.037	0.035	0.035	0.034	0.032	0.031	0.031	0.030	0.030	0.030	0.029	0.029	0.028	0.027
Manipur	Lower bound	0.020	0.027	0.035	0.048	0.066	0.080	0.065	0.054	0.049	0.049	0.043	0.038	0.037	0.035	0.033	0.031	0.029	0.027	0.027	0.026	0.024	0.023	0.022	0.021	0.021	0.020	0.019	0.019	0.018	0.017
Madhya Pradesh	Upper bound	0.799	0.833	0.626	0.500	0.481	0.475	0.469	0.452	0.433	0.412	0.335	0.279	0.216	0.175	0.146	0.116	0.093	0.076	0.068	0.065	0.064	0.063	0.063	0.063	0.062	0.062	0.062	0.061	0.061	0.061
Madhya Pradesh	Point	0.494	0.511	0.430	0.391	0.397	0.397	0.393	0.371	0.351	0.333	0.272	0.226	0.174	0.137	0.113	0.090	0.074	0.063	0.059	0.057	0.055	0.054	0.053	0.052	0.050	0.050	0.049	0.048	0.048	0.048
Madhya Pradesh	Lower bound	0.159	0.173	0.198	0.229	0.276	0.301	0.320	0.321	0.313	0.304	0.236	0.181	0.129	0.097	0.081	0.068	0.059	0.052	0.050	0.047	0.045	0.043	0.041	0.040	0.038	0.036	0.035	0.034	0.033	0.033
Mizoram	Upper bound	0.896	0.875	0.928	0.864	0.592	0.488	0.451	0.572	0.749	0.698	0.483	0.372	0.274	0.206	0.164	0.151	0.145	0.129	0.110	0.109	0.114	0.112	0.109	0.106	0.101	0.097	0.094	0.091	0.082	0.075
Mizoram	Point	0.550	0.527	0.556	0.539	0.386	0.312	0.272	0.257	0.240	0.200	0.162	0.147	0.134	0.126	0.119	0.115	0.112	0.102	0.089	0.091	0.096	0.096	0.094	0.091	0.086	0.083	0.080	0.076	0.068	0.062
Mizoram	Lower bound	0.086	0.074	0.076	0.076	0.059	0.052	0.050	0.067	0.097	0.109	0.096	0.094	0.089	0.087	0.082	0.080	0.080	0.075	0.068	0.071	0.077	0.079	0.078	0.077	0.072	0.069	0.066	0.062	0.054	0.047
Nagaland	Upper bound	0.884	0.888	0.901	0.756	0.700	0.669	0.787	0.865	0.599	0.405	0.294	0.208	0.165	0.146	0.119	0.094	0.093	0.093	0.089	0.087	0.088	0.084	0.079	0.076	0.073	0.071	0.068	0.067	0.065	0.060
Nagaland	Point	0.626	0.635	0.624	0.492	0.417	0.348	0.279	0.214	0.154	0.135	0.125	0.121	0.115	0.108	0.092	0.075	0.075	0.077	0.074	0.075	0.076	0.073	0.069	0.067	0.064	0.063	0.060	0.059	0.057	0.053
Nagaland	Lower bound	0.071	0.073	0.079	0.068	0.069	0.072	0.092	0.120	0.104	0.095	0.090	0.090	0.093	0.082	0.072	0.060	0.061	0.064	0.062	0.062	0.063	0.061	0.059	0.057	0.054	0.054	0.051	0.050	0.049	0.044
Odisha	Upper bound	0.607	0.637	0.572	0.446	0.417	0.414	0.398	0.391	0.375	0.366	0.346	0.323	0.293	0.262	0.229	0.198	0.170	0.137	0.118	0.101	0.083	0.073	0.065	0.059	0.055	0.054	0.053	0.052	0.052	0.051
Odisha	Point	0.403	0.428	0.416	0.346	0.348	0.354	0.348	0.345	0.332	0.323	0.306	0.284	0.255	0.224	0.192	0.162	0.134	0.108	0.092	0.079	0.067	0.061	0.056	0.053	0.049	0.047	0.045	0.044	0.043	0.042
Odisha	Lower bound	0.198	0.240	0.261	0.232	0.259	0.272	0.286	0.300	0.298	0.293	0.273	0.240	0.207	0.171	0.140	0.114	0.096	0.079	0.070	0.064	0.056	0.052	0.048	0.044	0.041	0.039	0.037	0.036	0.035	0.034
Punjab	Upper bound	0.479	0.639	0.567	0.474	0.450	0.428	0.408	0.388	0.361	0.325	0.294	0.261	0.223	0.184	0.150	0.116	0.091	0.078	0.066	0.062	0.060	0.060	0.060	0.059	0.057	0.055	0.054	0.052	0.050	0.045
Punjab	Point	0.408	0.405	0.408	0.404	0.395	0.385	0.366	0.346	0.312	0.275	0.234	0.192	0.152	0.118	0.096	0.076	0.064	0.060	0.054	0.052	0.050	0.048	0.047	0.046	0.044	0.043	0.042	0.040	0.038	0.033
Punjab	Lower bound	0.368	0.367	0.331	0.291	0.302	0.312	0.283	0.236	0.178	0.128	0.098	0.082	0.075	0.069	0.064	0.056	0.051	0.049	0.045	0.043	0.041	0.039	0.038	0.036	0.034	0.033	0.032	0.030	0.028	0.024
Rajasthan	Upper bound	1.905	0.402	0.357	0.325	0.270	0.237	0.234	0.227	0.222	0.213	0.205	0.197	0.190	0.180	0.173	0.168	0.157	0.149	0.141	0.132	0.122	0.113	0.104	0.095	0.086	0.077	0.069	0.063	0.057	0.050
Rajasthan	Point	0.444	0.358	0.316	0.288	0.243	0.216	0.216	0.211	0.206	0.196	0.188	0.181	0.173	0.164	0.157	0.153	0.143	0.136	0.128	0.120	0.111	0.102	0.093	0.084	0.076	0.067	0.060	0.053	0.048	0.042
Rajasthan	Lower bound	0.385	0.311	0.275	0.247	0.213	0.191	0.193	0.193	0.190	0.180	0.173	0.166	0.158	0.149	0.143	0.139	0.129	0.123	0.116	0.108	0.098	0.090	0.081	0.073	0.065	0.056	0.049	0.043	0.038	0.031
Sikkim	Upper bound	0.361	0.277	0.262	0.221	0.215	0.189	0.172	0.172	0.159	0.174	0.174	0.184	0.180	0.177	0.173	0.185	0.182	0.184	0.181	0.167	0.166	0.154	0.124	0.121	0.118	0.114	0.113	0.111	0.111	0.100
Sikkim	Point	0.276	0.223	0.213	0.180	0.178	0.157	0.143	0.145	0.134	0.149	0.148	0.156	0.152	0.148	0.144	0.154	0.153	0.155	0.153	0.140	0.137	0.127	0.059	0.064	0.068	0.071	0.073	0.072	0.065	0.055
Sikkim	Lower bound	0.233	0.189	0.181	0.154	0.151	0.136	0.124	0.127	0.117	0.129	0.128	0.134	0.130	0.124	0.118	0.122	0.118	0.118	0.115	0.105	0.101	0.088	0.046	0.048	0.045	0.044	0.042	0.041	0.037	0.030
Tamil Nadu	Upper bound	1.118	0.881	0.785	0.704	0.606	0.576	0.633	0.493	0.339	0.206	0.118	0.079	0.046	0.039	0.037	0.037	0.036	0.036	0.034	0.034	0.033	0.031	0.031	0.029	0.028	0.027	0.026	0.026	0.025	0.024
Tamil Nadu	Point	0.527	0.512	0.510	0.467	0.389	0.301	0.222	0.135	0.086	0.064	0.054	0.049	0.035	0.031	0.030	0.028	0.027	0.026	0.025	0.024	0.024	0.022	0.022	0.021	0.020	0.019	0.018	0.017	0.017	0.016
Tamil Nadu	Lower bound	0.155	0.093	0.071	0.059	0.052	0.050	0.055	0.051	0.048	0.046	0.042	0.039	0.028	0.025	0.024	0.021	0.020	0.018	0.017	0.016	0.015	0.014	0.013	0.012	0.012	0.011	0.010	0.010	0.009	0.008
Tripura	Upper bound	1.426	0.394	0.352	0.271	0.254	0.245	0.250	0.251	0.236	0.239	0.241	0.238	0.233	0.231	0.229	0.232	0.230	0.228	0.222	0.219	0.215	0.212	0.205	0.200	0.192	0.185	0.185	0.178	0.173	0.169
Tripura	Point	0.328	0.299	0.272	0.221	0.212	0.205	0.212	0.214	0.209	0.212	0.216	0.214	0.213	0.208	0.209	0.212	0.211	0.209	0.203	0.202	0.198	0.196	0.190	0.185	0.177	0.169	0.170	0.163	0.158	0.153
Tripura	Lower bound	0.235	0.217	0.199	0.148	0.115	0.112	0.125	0.127	0.137	0.144	0.155	0.159	0.163	0.162	0.165	0.172	0.176	0.179	0.178	0.179	0.175	0.175	0.170	0.166	0.160	0.153	0.154	0.147	0.141	0.136
Uttarakhand	Upper bound	0.211	0.210	0.213	0.208	0.212	0.201	0.196	0.194	0.185	0.182	0.171	0.169	0.154	0.153	0.146	0.140	0.135	0.133	0.123	0.118	0.111	0.103	0.097	0.089	0.081	0.075	0.069	0.064	0.058	0.052
Uttarakhand	Point	0.189	0.188	0.191	0.186	0.191	0.181	0.174	0.170	0.162	0.157	0.147	0.145	0.132	0.129	0.123	0.117	0.113	0.111	0.103	0.099	0.093	0.086	0.081	0.074	0.066	0.061	0.056	0.051	0.046	0.041

Uttarakhand	Lower bound	0.163	0.163	0.165	0.165	0.169	0.161	0.154	0.150	0.142	0.136	0.127	0.125	0.113	0.110	0.105	0.100	0.097	0.096	0.090	0.086	0.080	0.075	0.069	0.063	0.056	0.052	0.047	0.042	0.037	0.032
Uttar Pradesh	Upper bound	0.211	0.214	0.214	0.213	0.205	0.202	0.193	0.187	0.177	0.172	0.163	0.154	0.152	0.142	0.131	0.128	0.124	0.117	0.112	0.106	0.099	0.093	0.087	0.080	0.074	0.069	0.063	0.058	0.054	0.049
Uttar Pradesh	Point	0.191	0.194	0.193	0.192	0.186	0.182	0.174	0.168	0.159	0.154	0.145	0.137	0.135	0.126	0.115	0.114	0.110	0.104	0.099	0.094	0.088	0.082	0.076	0.070	0.064	0.058	0.053	0.048	0.044	0.040
Uttar Pradesh	Lower bound	0.168	0.173	0.174	0.175	0.167	0.163	0.154	0.148	0.140	0.135	0.127	0.120	0.118	0.109	0.101	0.100	0.096	0.091	0.087	0.082	0.076	0.071	0.065	0.059	0.054	0.049	0.044	0.039	0.035	0.030
West Bengal	Upper bound	0.836	0.832	0.807	0.746	0.624	0.383	0.188	0.097	0.066	0.057	0.054	0.053	0.056	0.061	0.066	0.069	0.067	0.069	0.068	0.071	0.075	0.074	0.074	0.072	0.070	0.069	0.068	0.067	0.065	0.064
West Bengal	Point	0.728	0.729	0.717	0.674	0.549	0.330	0.152	0.080	0.057	0.051	0.047	0.046	0.048	0.052	0.055	0.057	0.054	0.055	0.053	0.055	0.055	0.055	0.055	0.054	0.053	0.052	0.052	0.051	0.050	0.050
West Bengal	Lower bound	0.687	0.685	0.600	0.561	0.480	0.188	0.079	0.054	0.043	0.037	0.035	0.035	0.039	0.043	0.046	0.047	0.044	0.044	0.040	0.037	0.036	0.036	0.037	0.036	0.035	0.034	0.034	0.035	0.034	0.034
Andaman & Nicobar Islands	Upper bound	0.366	0.256	0.241	0.229	0.209	0.186	0.153	0.156	0.158	0.153	0.154	0.159	0.154	0.155	0.154	0.149	0.148	0.144	0.147	0.142	0.138	0.135	0.137	0.138	0.137	0.137	0.130	0.100	0.091	0.093
Andaman & Nicobar Islands	Point	0.232	0.207	0.197	0.187	0.172	0.155	0.129	0.133	0.136	0.132	0.133	0.138	0.134	0.134	0.133	0.128	0.127	0.123	0.125	0.121	0.118	0.114	0.116	0.117	0.116	0.117	0.103	0.049	0.040	0.041
Andaman & Nicobar Islands	Lower bound	0.185	0.167	0.160	0.154	0.142	0.128	0.108	0.111	0.115	0.111	0.112	0.117	0.113	0.113	0.111	0.106	0.102	0.098	0.098	0.093	0.089	0.084	0.084	0.082	0.080	0.078	0.068	0.029	0.025	0.024
Chandigarh	Upper bound	0.778	0.777	0.720	0.640	0.560	0.497	0.446	0.396	0.352	0.304	0.234	0.204	0.163	0.123	0.103	0.089	0.080	0.070	0.068	0.065	0.066	0.065	0.064	0.065	0.064	0.066	0.069	0.070	0.071	0.074
Chandigarh	Point	0.514	0.500	0.472	0.465	0.444	0.410	0.360	0.296	0.235	0.179	0.121	0.101	0.083	0.070	0.067	0.064	0.061	0.055	0.054	0.051	0.050	0.049	0.049	0.050	0.049	0.051	0.052	0.054	0.054	0.056
Chandigarh	Lower bound	0.428	0.421	0.403	0.403	0.374	0.188	0.084	0.062	0.056	0.053	0.046	0.048	0.046	0.041	0.040	0.039	0.037	0.032	0.032	0.030	0.030	0.029	0.029	0.029	0.028	0.028	0.028	0.028	0.028	0.029
Dadra & Nagar Haveli	Upper bound	0.498	0.311	0.254	0.171	0.198	0.209	0.218	0.193	0.218	0.215	0.207	0.215	0.217	0.216	0.223	0.224	0.222	0.223	0.226	0.218	0.214	0.212	0.199	0.188	0.176	0.170	0.166	0.143	0.133	0.127
Dadra & Nagar Haveli	Point	0.250	0.258	0.213	0.144	0.168	0.180	0.189	0.168	0.191	0.188	0.182	0.191	0.192	0.191	0.196	0.197	0.194	0.195	0.197	0.190	0.186	0.184	0.173	0.162	0.151	0.145	0.142	0.122	0.114	0.108
Dadra & Nagar Haveli	Lower bound	0.214	0.222	0.184	0.125	0.147	0.159	0.168	0.149	0.169	0.168	0.161	0.167	0.167	0.163	0.165	0.161	0.156	0.153	0.150	0.143	0.137	0.129	0.118	0.107	0.096	0.089	0.085	0.071	0.064	0.059
Daman & Diu	Upper bound	0.575	0.614	0.640	0.569	0.616	0.575	0.578	0.515	0.460	0.405	0.379	0.330	0.255	0.204	0.196	0.156	0.121	0.107	0.107	0.085	0.110	0.109	0.106	0.102	0.098	0.098	0.097	0.084	0.080	0.079
Daman & Diu	Point	0.436	0.477	0.499	0.438	0.478	0.441	0.438	0.380	0.310	0.247	0.202	0.150	0.104	0.081	0.083	0.076	0.071	0.072	0.077	0.061	0.079	0.078	0.077	0.075	0.073	0.074	0.074	0.065	0.063	0.063
Daman & Diu	Lower bound	0.351	0.387	0.412	0.355	0.286	0.186	0.129	0.088	0.072	0.068	0.067	0.063	0.056	0.054	0.059	0.056	0.053	0.055	0.059	0.046	0.058	0.058	0.055	0.053	0.049	0.048	0.048	0.043	0.041	0.041
Puducherry	Upper bound	1.950	1.247	0.566	0.370	0.289	0.251	0.199	0.165	0.163	0.165	0.171	0.167	0.169	0.169	0.175	0.185	0.185	0.174	0.171	0.169	0.169	0.167	0.071	0.071	0.073	0.076	0.081	0.084	0.091	0.103
Puducherry	Point	0.373	0.314	0.261	0.221	0.196	0.177	0.150	0.128	0.130	0.133	0.140	0.140	0.144	0.144	0.150	0.161	0.160	0.150	0.144	0.140	0.139	0.136	0.056	0.057	0.058	0.059	0.063	0.065	0.067	0.070
Puducherry	Lower bound	0.017	0.015	0.038	0.054	0.050	0.053	0.050	0.045	0.051	0.057	0.068	0.076	0.086	0.098	0.118	0.131	0.130	0.119	0.112	0.107	0.101	0.097	0.042	0.042	0.041	0.040	0.042	0.042	0.042	0.043
Telangana	Upper bound	0.974	0.620	0.495	0.459	0.429	0.365	0.305	0.263	0.207	0.160	0.119	0.082	0.066	0.051	0.045	0.041	0.040	0.040	0.039	0.040	0.040	0.039	0.039	0.037	0.037	0.037	0.036	0.036	0.035	0.031
Telangana	Point	0.564	0.362	0.338	0.357	0.355	0.321	0.269	0.215	0.158	0.115	0.083	0.059	0.050	0.041	0.037	0.035	0.033	0.032	0.030	0.029	0.029	0.028	0.027	0.026	0.025	0.025	0.023	0.023	0.022	0.019
Telangana	Lower bound	0.195	0.203	0.230	0.247	0.205	0.127	0.089	0.070	0.060	0.055	0.049	0.040	0.038	0.032	0.030	0.028	0.025	0.023	0.021	0.020	0.018	0.017	0.016	0.015	0.014	0.013	0.012	0.012	0.011	0.009
India	Upper bound	1.250	0.964	0.700	0.514	0.312	0.254	0.233	0.209	0.185	0.151	0.120	0.095	0.078	0.065	0.059	0.055	0.053	0.051	0.050	0.050	0.049	0.049	0.048	0.047	0.044	0.043	0.042	0.041	0.039	0.037
India	Point	0.232	0.198	0.190	0.200	0.202	0.200	0.190	0.172	0.151	0.122	0.098	0.078	0.065	0.056	0.051	0.048	0.045	0.044	0.042	0.042	0.041	0.040	0.039	0.038	0.036	0.035	0.034	0.033	0.031	0.029
India	Lower bound	0.134	0.134	0.136	0.152	0.159	0.159	0.152	0.135	0.112	0.091	0.076	0.062	0.054	0.047	0.043	0.041	0.039	0.038	0.036	0.035	0.034	0.033	0.032	0.030	0.029	0.028	0.027	0.026	0.024	0.022

Table b. IMR by States/UTs in India, 1990-2019

State/UT	Range	1990	1991	1992	1993	1994	1995	1996	1997	1998	1999	2000	2001	2002	2003	2004	2005	2006	2007	2008	2009	2010	2011	2012	2013	2014	2015	2016	2017	2018	2019
Andhra Pradesh	Upper	59.322	51.820	40.037	34.214	31.022	29.885	26.044	20.933	17.207	11.057	6.115	2.978	1.717	1.005	0.661	0.461	0.361	0.300	0.283	0.280	0.281	0.282	0.284	0.280	0.302	0.306	0.307	0.315	0.327	0.338
Andhra Pradesh	Point	27.913	26.300	24.092	19.533	16.141	15.340	13.874	11.237	8.561	5.195	3.104	1.666	1.018	0.630	0.446	0.338	0.274	0.232	0.215	0.202	0.192	0.184	0.178	0.171	0.179	0.180	0.177	0.182	0.192	0.199
Andhra Pradesh	Lower	0.114	0.084	0.065	0.055	1.276	6.955	8.059	5.705	3.945	2.105	1.267	0.784	0.581	0.423	0.328	0.253	0.200	0.162	0.143	0.129	0.119	0.109	0.101	0.095	0.094	0.094	0.092	0.092	0.095	0.099
Arunachal Pradesh	Upper	234.247	23.689	15.521	15.947	7.999	8.799	5.749	4.269	4.885	4.643	4.315	4.687	4.534	4.201	3.995	4.065	4.331	4.657	5.809	5.839	5.699	5.175	4.522	4.523	4.884	4.562	4.473	4.491	4.221	4.742
Arunachal Pradesh	Point	13.410	10.199	8.002	9.000	5.000	5.500	3.667	2.750	3.250	3.200	3.000	3.333	3.286	3.125	3.111	3.200	3.400	3.600	4.222	4.200	4.091	3.769	3.313	3.294	3.529	3.316	3.250	3.333	3.130	3.476
Arunachal Pradesh	Lower	7.133	6.409	5.623	6.693	3.835	4.314	2.885	2.173	2.569	2.487	2.347	2.597	2.550	2.427	2.420	2.465	2.577	2.672	2.993	2.907	2.797	2.512	2.193	2.100	2.136	1.949	1.843	1.849	1.666	1.862
Assam	Upper	152.821	17.209	12.206	8.410	5.634	4.729	4.481	4.249	3.989	3.920	3.752	3.520	3.357	3.180	3.064	2.891	2.820	2.970	3.058	3.198	3.256	3.341	3.157	3.064	2.933	2.721	2.614	2.538	2.386	2.313
Assam	Point	9.583	7.519	6.380	5.170	3.966	3.603	3.508	3.386	3.243	3.220	3.131	2.965	2.852	2.722	2.621	2.461	2.391	2.485	2.495	2.534	2.532	2.557	2.425	2.319	2.222	2.068	1.992	1.933	1.844	1.790
Assam	Lower	5.814	5.260	4.647	3.947	3.101	2.850	2.827	2.750	2.645	2.672	2.626	2.539	2.433	2.328	2.229	2.085	2.000	2.054	2.053	2.060	2.060	2.087	1.976	1.898	1.826	1.707	1.650	1.612	1.548	1.512
Bihar	Upper	182.203	26.975	20.506	15.524	11.955	7.956	6.839	6.327	5.901	5.576	5.323	5.028	4.718	4.403	4.199	3.927	3.768	3.917	4.222	4.595	4.650	4.711	4.315	4.168	4.035	3.827	3.630	3.360	3.130	2.930
Bihar	Point	26.067	16.982	12.946	10.313	8.519	6.044	5.367	5.045	4.769	4.576	4.418	4.211	3.992	3.736	3.547	3.320	3.172	3.264	3.467	3.704	3.726	3.746	3.446	3.300	3.186	3.018	2.873	2.655	2.496	2.358
Bihar	Lower	12.834	9.472	8.224	7.197	6.364	4.663	4.134	3.916	3.746	3.629	3.587	3.487	3.334	3.118	2.984	2.802	2.706	2.819	3.010	3.211	3.205	3.191	2.928	2.825	2.713	2.572	2.446	2.257	2.127	2.003
Chhattishgarh	Upper	43.176	33.026	30.063	28.332	25.889	21.717	16.339	11.039	7.177	4.140	2.314	1.371	0.963	0.797	0.713	0.680	0.675	0.680	0.747	0.835	0.861	0.931	0.970	1.065	1.215	1.329	1.453	1.540	1.562	1.712
Chhattishgarh	Point	24.800	20.071	19.349	19.031	18.063	15.813	12.218	8.354	5.263	2.987	1.725	1.092	0.809	0.692	0.625	0.587	0.571	0.573	0.622	0.700	0.719	0.781	0.818	0.908	1.049	1.163	1.290	1.375	1.414	1.545
Chhattishgarh	Lower	8.082	9.756	12.238	13.787	13.736	12.423	9.225	5.058	2.609	1.464	0.979	0.734	0.609	0.550	0.515	0.485	0.465	0.454	0.485	0.527	0.533	0.580	0.606	0.672	0.789	0.879	1.010	1.093	1.154	1.316
Delhi	Upper	8.246	7.049	6.472	6.066	5.699	5.464	5.224	5.129	4.953	4.765	4.535	4.200	3.981	3.704	3.587	4.395	5.442	5.187	4.787	4.619	4.719	4.510	4.126	3.884	3.736	3.521	3.618	3.914	3.129	2.741
Delhi	Point	5.587	5.199	4.972	4.765	4.504	4.355	4.117	3.991	3.855	3.669	3.539	3.338	3.167	2.984	2.902	3.464	4.113	3.925	3.660	3.516	3.545	3.385	3.108	2.934	2.802	2.660	2.811	2.963	2.386	2.174
Delhi	Lower	4.560	4.383	4.220	4.042	3.817	3.644	3.405	3.280	3.168	3.039	2.926	2.755	2.597	2.454	2.376	2.775	3.214	3.066	2.880	2.757	2.810	2.693	2.488	2.378	2.291	2.195	2.321	2.378	1.931	1.798
Goa	Upper	133.623	125.504	109.915	101.814	77.920	48.854	30.895	15.340	7.281	3.423	1.654	0.981	0.521	0.409	0.362	0.329	0.340	0.450	0.479	0.510	0.567	0.586	0.557	0.539	0.535	0.527	0.523	0.526	0.536	0.534
Goa	Point	76.500	73.286	65.043	53.406	31.727	12.660	4.730	1.988	1.180	0.787	0.561	0.422	0.245	0.200	0.167	0.144	0.137	0.172	0.172	0.175	0.192	0.202	0.204	0.211	0.218	0.230	0.242	0.254	0.272	0.281
Goa	Lower	28.180	28.908	28.827	25.605	5.145	1.650	1.061	0.755	0.551	0.379	0.267	0.186	0.107	0.081	0.066	0.055	0.047	0.059	0.061	0.064	0.066	0.066	0.064	0.066	0.063	0.069	0.074	0.084	0.086	0.089
Gujarat	Upper	19.023	19.688	19.410	19.734	20.340	20.642	19.553	17.343	14.294	11.998	9.934	8.012	6.267	5.073	3.781	3.032	2.361	1.932	1.856	1.776	1.769	1.702	1.648	1.613	1.607	1.677	1.702	1.745	1.717	1.663
Gujarat	Point	14.083	14.541	14.576	14.766	15.053	14.429	13.532	12.370	10.388	8.869	7.274	5.894	4.594	3.602	2.642	2.084	1.592	1.308	1.267	1.254	1.325	1.346	1.363	1.364	1.357	1.406	1.406	1.428	1.402	1.378
Gujarat	Lower	11.229	11.460	11.462	11.372	11.432	10.620	9.834	8.771	7.315	6.422	5.263	4.051	2.872	2.147	1.609	1.269	1.023	0.901	0.910	0.959	1.062	1.115	1.140	1.151	1.148	1.193	1.181	1.200	1.177	1.161
Himachal Pradesh	Upper	5.706	4.994	4.376	3.907	3.736	3.434	3.166	2.997	2.817	2.659	2.530	2.312	2.181	1.953	1.866	1.753	1.758	2.101	2.473	2.672	2.838	3.038	1.586	1.560	1.511	1.366	1.453	1.636	1.727	1.673
Himachal Pradesh	Point	4.164	3.831	3.520	3.168	3.036	2.823	2.615	2.463	2.330	2.211	2.096	1.919	1.813	1.631	1.557	1.468	1.473	1.695	1.929	2.065	2.164	2.295	1.038	1.042	1.060	1.064	1.160	1.290	1.311	1.235
Himachal Pradesh	Lower	3.464	3.288	3.034	2.689	2.547	2.368	2.196	2.075	1.961	1.854	1.761	1.617	1.528	1.378	1.319	1.253	1.240	1.391	1.586	1.711	1.808	1.899	0.830	0.840	0.862	0.882	0.953	1.025	1.021	0.929
Haryana	Upper	21.337	21.100	19.851	19.966	18.985	18.468	17.698	16.393	15.472	13.818	12.309	10.627	8.845	7.077	5.685	4.251	3.530	2.731	2.327	1.901	1.588	1.330	1.166	1.113	1.196	1.264	1.212	1.410	1.473	1.376
Haryana	Point	15.000	14.938	14.192	14.268	13.723	13.505	13.110	12.226	11.387	9.873	8.541	6.821	5.285	3.880	2.891	2.088	1.680	1.310	1.191	1.086	1.013	0.958	0.925	0.949	1.021	1.066	1.008	1.178	1.239	1.160
Haryana	Lower	11.137	11.353	10.851	10.928	10.685	10.598	10.526	10.010	9.260	7.506	5.776	4.125	3.008	2.137	1.581	1.184	1.027	0.865	0.859	0.844	0.817	0.797	0.779	0.808	0.863	0.895	0.829	0.955	0.996	0.921
Jharkhand	Upper	168.992	23.526	18.369	13.362	10.179	6.663	6.079	5.481	5.111	4.872	4.589	4.316	4.160	3.916	3.566	3.424	3.422	3.541	4.002	4.336	4.451	4.792	4.607	4.465	4.150	3.947	3.933	3.688	3.302	3.027
Jharkhand	Point	18.182	13.706	10.885	8.675	7.333	5.078	4.733	4.364	4.204	4.045	3.826	3.585	3.457	3.253	2.965	2.855	2.840	2.892	3.156	3.358	3.370	3.574	3.445	3.267	3.007	2.878	2.755	2.561	2.320	2.192
Jharkhand	Lower	8.263	7.347	6.692	5.968	5.385	3.738	3.564	3.373	3.269	3.249	3.136	2.969	2.878	2.719	2.493	2.389	2.394	2.444	2.688	2.847	2.846	2.967	2.830	2.659	2.444	2.353	2.231	2.077	1.892	1.783
Jammu & Kashmir	Upper	373.151	31.699	18.203	12.492	8.679	7.089	4.583	3.982	3.566	3.385	3.296	3.047	3.044	3.140	2.946	2.938	3.522	4.224	5.070	5.888	5.928	6.113	5.732	5.320	5.036	4.758	4.479	4.222	3.900	3.672
Jammu & Kashmir	Point	17.429	11.167	8.333	6.720	5.500	4.727	3.130	2.818	2.610	2.544	2.549	2.386	2.417	2.529	2.366	2.340	2.691	3.000	3.344	3.792	3.672	3.589	3.228	2.993	2.826	2.753	2.523	2.302	2.041	1.831
Jammu & Kashmir	Lower	8.252	6.318	5.272	4.612	3.953	3.533	2.386	2.148	2.005	1.972	2.001	1.913	1.993	2.114	1.999	1.939	2.093	2.157	2.281	2.406	2.392	2.311	2.251	2.296	2.142	1.974	1.795	1.644	1.459	1.304
Karnataka	Upper	93.974	77.461	43.811	35.483	24.969	20.897	17.165	12.974	9.618	6.776	4.675	2.910	1.784	1.089	0.719	0.517	0.390	0.307	0.267	0.257	0.264	0.277	0.283	0.291	0.292	0.293	0.295	0.293	0.298	0.322
Karnataka	Point	30.412	28.959	17.578	16.179	12.789	12.195	10.781	8.742	6.522	4.310	2.727	1.642	1.020	0.645	0.460	0.349	0.274	0.228	0.196	0.184	0.176	0.173	0.164	0.160	0.156	0.151	0.150	0.150	0.156	0.173
Karnataka	Lower	4.851	5.202	4.855	6.107	6.348	7.602	7.209	4.644	2.716	1.554	1.043	0.710	0.523	0.387	0.303	0.247	0.193	0.156	0.127	0.111	0.100	0.092	0.085	0.081	0.077	0.074	0.072	0.071	0.075	0.088
Kerala	Upper	29.647	31.654	30.275	28.356	29.025	27.573	25.600	24.446	21.063	15.961	12.068	8.777	5.538	3.714	2.537	1.817	1.671	1.488	1.198	1.060	1.109	1.166	1.207	1.286	1.303	1.311	1.318	1.334	1.353	1.485
Kerala	Point	18.429	19.909	19.474	18.656	18.585	17.805	16.460	15.301	13.051	9.922	7.222	5.020	3.011	1.942	1.321	0.975	0.929	0.902	0.836	0.808	0.819	0.828	0.830	0.874	0.888	0.914	0.950	0.993	1.046	1.166
Kerala	Lower	12.587	14.084	13.891	13.576	13.423	12.730	10.889	8.651	5.691	3.439	2.227	1.549	1.029	0.790	0.672	0.590	0.655	0.687	0.655	0.622	0.638	0.636	0.635	0.662	0.664	0.672	0.690	0.718	0.762	0.862
Meghalaya	Upper	#DIV/0!	385.458	160.365	85.389	33.411	22.795	14.805	11.636	9.153	7.626	6.364	5.615	5.178	5.666	6.464	7.576	8.869	9.970	10.373	10.666	10.098	9.088	7.683	6.229	5.428	4.575	3.736	3.158	2.695	2.450
Meghalaya	Point	33.000	24.667	12.833	10.125	5.000	3.938	3.250	2.833	2.571	2.438	2.444	2.585	2.956	3.780	4.947	5.939	7.101	8.042	8.449	8.739	8.218	7.140	5.736	4.438	3.612	2.918	2.401	2.072	1.884	1.821
Meghalaya	Lower	#DIV/0!	8.817	5.241	4.877	2.816	2.590	2.436	2.280	2.160	2.120	2.161	2.270	2.565	3.215	4.113	4.822	5.671	6.487	6.915	7.293	6.819	5.554	4.138	3.057	2.442	2.007	1.720	1.586	1.517	1.521
Maharashtra	Upper	151.495	99.684	62.714	33.166	11.775	4.318	2.211	1.457	1.061	0.834	0.681	0.569	0.506	0.457	0.418	0.399	0.390	0.397	0.437	0.486	0.539	0.599	0.633	0.704	0.781	0.810	0.826	0.845	0.851	0.894
Maharashtra	Point	6.971	4.256	2.849	2.022	1.460	1.111	0.878	0.711	0.587	0.495	0.424	0.370	0.328	0.295	0.272	0.259	0.253	0.260	0.284	0.322	0.365	0.406	0.436	0.493	0.557	0.575	0.591	0.622	0.636	0.664
Maharashtra	Lower	1.905	1.627	1.376	1.125	0.867	0.712	0.586	0.486	0.412	0.356	0.312	0.270	0.237	0.205	0.184	0.172	0.165	0.167	0.179	0.200	0.226	0.250	0.274	0.313	0.358	0.366	0.385	0.409	0.422	0.423
Manipur	Upper	116.759	120.459	91.324	86.694	76.720	60.898	45.181	27.935	13.021	6.476	3.053	1.646	1.136	0.859	0.692	0.620	0.635	0.660	0.735	0.784	0.754	0.782	0.776	0.802	0.852	0.815	0.861	0.872	0.951	0.979
Manipur	Point	38.375	37.875	33.350	24.843	18.479	13.429	9.433	6.138	3.688	2.321	1.395	0.940	0.770	0.641	0.549	0.506	0.516	0.530	0.561	0.572	0.535	0.554	0.550	0.572	0.607	0.580	0.621	0.638	0.707	0.733
Manipur	Lower	1.120	1.502	1.882	2.412	3.020	3.257	2.346	1.686	1.308	1.126	0.849	0.656	0.562	0.483	0.424	0.401	0.402	0.409	0.427	0.432	0.399	0.404	0.390	0.402	0.421	0.388	0.410	0.418	0.458	0.472
Madhya Pradesh	Upper	43.348	42.363	29.302	22.462	20.511	19.247	18.603	17.539	16.000	15.207	11.512	8.504	5.962	4.213	3.067	2.161	1.628	1.288	1.121	0.987	0.918	0.905	0.923	0.955	1.012	1.070	1.154	1.231	1.281	1.420
Madhya Pradesh	Point	18.917	18.917	15.214	13.412	13.180	12.894	12.554	11.610	10.675	9.815	7.536	5.778	4.043	2.873	2.118	1.521	1.166	0.945	0.865	0.805	0.783	0.790	0.807	0.830	0.871	0.912	0.983	1.051	1.095	1.225

Madhya Pradesh	Lower	2.723	3.796	4.000	4.881	6.109	7.070	7.777	8.064	7.863	7.618	5.925	4.270	2.721	1.894	1.370	1.018	0.818	0.706	0.672	0.636	0.625	0.620	0.626	0.630	0.658	0.679	0.721	0.772	0.810	0.920
Mizoram	Upper	67.884	84.410	81.977	79.203	43.956	33.153	29.565	33.362	47.875	50.284	39.370	23.188	13.449	7.570	4.541	3.164	2.444	2.137	1.812	1.930	2.214	2.383	2.484	2.573	2.942	3.229	3.361	3.485	3.699	3.841
Mizoram	Point	25.000	29.200	27.667	27.958	17.535	12.806	9.910	8.382	7.172	5.376	3.916	3.205	2.680	2.323	2.049	1.878	1.773	1.604	1.416	1.565	1.830	1.971	2.041	2.113	2.352	2.557	2.637	2.722	2.888	2.972
Mizoram	Lower	2.330	1.834	1.433	1.334	0.870	0.721	0.688	0.842	1.446	1.975	2.218	1.935	1.721	1.578	1.413	1.327	1.273	1.174	1.031	1.113	1.294	1.411	1.492	1.596	1.837	1.994	2.025	2.069	2.190	2.245
Nagaland	Upper	79.136	76.008	77.538	58.745	50.516	46.389	53.307	54.107	36.151	22.363	12.372	7.234	4.268	2.690	1.895	1.439	1.388	1.478	1.539	1.657	2.008	2.219	2.333	2.399	2.560	2.491	2.203	2.377	2.461	2.579
Nagaland	Point	38.500	37.182	36.571	25.082	19.125	14.144	9.916	6.532	3.985	2.994	2.407	2.080	1.778	1.601	1.322	1.071	1.081	1.175	1.253	1.385	1.707	1.909	2.017	2.089	2.204	2.100	1.897	2.041	2.107	2.216
Nagaland	Lower	1.460	1.219	1.186	0.949	0.919	0.962	1.288	1.718	1.791	1.871	1.739	1.627	1.433	1.338	1.114	0.900	0.886	0.959	1.015	1.128	1.392	1.560	1.653	1.736	1.858	1.776	1.651	1.785	1.851	1.942
Odisha	Upper	26.104	25.751	23.180	18.000	15.882	15.226	14.180	13.494	12.558	12.078	11.123	10.130	8.806	7.448	6.063	4.762	3.727	2.833	2.411	1.954	1.634	1.353	1.187	1.103	1.049	1.048	1.075	1.115	1.143	1.187
Odisha	Point	12.900	13.353	13.259	10.850	10.633	10.622	10.231	10.056	9.516	9.114	8.440	7.561	6.534	5.444	4.363	3.389	2.592	1.938	1.634	1.354	1.159	1.018	0.939	0.921	0.911	0.915	0.925	0.949	0.962	0.997
Odisha	Lower	3.888	4.762	6.165	5.831	6.553	7.039	7.083	7.480	7.360	7.227	6.627	5.758	4.632	3.535	2.657	1.973	1.513	1.146	1.024	0.906	0.837	0.792	0.781	0.795	0.798	0.803	0.812	0.819	0.830	0.851
Punjab	Upper	28.025	37.317	31.404	25.953	23.788	22.460	20.882	19.089	16.692	13.744	11.635	9.250	7.045	5.151	3.695	2.551	1.783	1.420	1.175	1.028	0.942	0.929	0.929	1.010	1.092	1.183	1.211	1.318	1.463	1.563
Punjab	Point	17.727	17.417	17.390	17.063	16.439	15.764	14.609	13.302	11.317	9.256	7.203	5.314	3.720	2.548	1.818	1.290	0.980	0.855	0.790	0.778	0.765	0.763	0.736	0.777	0.828	0.890	0.928	1.027	1.147	1.234
Punjab	Lower	13.275	12.910	12.799	12.051	11.472	10.999	9.331	6.958	4.511	2.758	1.857	1.359	1.068	0.923	0.827	0.710	0.634	0.614	0.601	0.612	0.608	0.609	0.591	0.622	0.659	0.700	0.736	0.803	0.899	0.934
Rajasthan	Upper	173.897	24.896	18.918	13.946	9.222	7.117	6.380	5.757	5.315	4.835	4.486	4.191	3.882	3.555	3.300	3.228	3.669	3.947	4.350	4.691	4.835	4.889	4.738	4.518	4.221	3.922	3.704	3.392	3.144	2.746
Rajasthan	Point	19.875	14.509	11.333	9.262	6.978	5.617	5.204	4.785	4.468	4.082	3.793	3.527	3.292	3.019	2.807	2.774	3.106	3.302	3.591	3.812	3.902	3.896	3.779	3.598	3.400	3.161	2.986	2.765	2.537	2.255
Rajasthan	Lower	9.322	8.209	7.317	6.582	5.251	4.392	4.186	3.934	3.776	3.486	3.262	3.043	2.867	2.637	2.473	2.449	2.750	2.910	3.123	3.267	3.320	3.281	3.155	2.985	2.817	2.615	2.477	2.279	2.096	1.882
Sikkim	Upper	31.706	16.979	13.055	11.224	5.868	5.681	3.704	4.142	3.033	3.691	3.230	3.776	3.365	3.104	3.327	3.437	3.803	4.391	6.863	8.758	8.104	8.062	4.886	5.248	5.397	5.284	5.511	5.638	5.619	5.144
Sikkim	Point	10.916	7.624	7.000	7.000	4.000	4.000	2.667	3.000	2.250	2.750	2.400	2.800	2.500	2.286	2.429	2.500	2.750	3.125	4.667	5.800	5.333	5.500	2.667	3.000	3.333	3.667	4.000	4.167	4.000	3.500
Sikkim	Lower	6.661	5.140	5.099	5.325	3.161	3.216	2.182	2.459	1.861	2.266	1.981	2.302	2.028	1.823	1.854	1.817	1.890	1.896	2.390	2.768	2.419	2.316	1.809	1.981	2.066	2.003	1.933	1.937	1.816	1.566
Tamil Nadu	Upper	107.418	74.805	63.040	50.168	39.868	32.194	36.129	28.280	15.444	7.558	3.508	1.778	0.834	0.537	0.415	0.365	0.401	0.422	0.490	0.562	0.636	0.688	0.724	0.734	0.764	0.793	0.820	0.838	0.865	0.866
Tamil Nadu	Point	26.537	24.640	23.925	21.011	16.165	11.153	7.116	3.633	1.923	1.200	0.865	0.675	0.431	0.345	0.303	0.281	0.301	0.297	0.319	0.345	0.374	0.402	0.427	0.443	0.476	0.504	0.530	0.556	0.582	0.595
Tamil Nadu	Lower	3.377	2.524	1.628	1.093	0.796	0.647	0.663	0.619	0.554	0.516	0.477	0.438	0.311	0.257	0.229	0.211	0.222	0.207	0.211	0.216	0.228	0.235	0.247	0.252	0.269	0.291	0.308	0.313	0.318	0.315
Tripura	Upper	188.595	25.595	17.626	10.492	10.387	5.666	6.702	5.188	6.042	5.394	6.607	6.276	6.131	5.365	5.758	5.686	5.765	6.963	9.262	11.317	12.461	12.192	13.986	14.615	14.789	15.153	16.226	15.572	16.581	16.666
Tripura	Point	13.292	10.778	8.760	6.395	7.000	4.000	5.000	4.000	4.667	4.250	5.250	5.000	5.000	4.375	4.667	4.636	4.692	5.615	7.167	8.667	9.538	9.313	10.875	11.333	11.650	12.000	12.520	12.172	12.903	12.914
Tripura	Lower	7.212	6.770	5.974	4.312	3.561	1.800	2.212	1.838	2.299	2.143	2.780	2.823	2.984	2.759	3.095	3.214	3.360	4.138	5.213	6.491	7.412	7.297	8.517	8.871	9.363	9.625	9.436	9.191	9.586	9.612
Uttarakhand	Upper	10.646	7.606	6.247	5.317	5.064	4.651	4.419	4.309	4.135	3.991	3.707	3.609	3.250	3.141	2.893	2.679	2.528	2.661	2.758	2.873	2.925	2.985	2.766	2.575	2.456	2.205	1.996	1.828	1.665	1.554
Uttarakhand	Point	5.000	4.515	4.329	4.020	4.000	3.716	3.506	3.397	3.171	3.029	2.775	2.689	2.388	2.273	2.090	1.937	1.832	1.893	1.908	1.953	1.980	1.991	1.875	1.769	1.691	1.558	1.434	1.328	1.230	1.167
Uttarakhand	Lower	3.844	3.553	3.456	3.269	3.290	3.046	2.844	2.734	2.548	2.419	2.210	2.132	1.901	1.813	1.668	1.553	1.465	1.493	1.485	1.518	1.542	1.563	1.494	1.429	1.364	1.268	1.170	1.089	1.013	0.952
Uttar Pradesh	Upper	5.695	5.198	4.763	4.491	4.156	3.982	3.687	3.484	3.235	3.050	2.813	2.563	2.474	2.242	1.977	1.924	1.918	1.878	1.897	1.987	1.992	1.979	1.924	1.846	1.779	1.719	1.662	1.626	1.559	1.503
Uttar Pradesh	Point	4.392	4.154	3.889	3.704	3.456	3.301	3.076	2.913	2.681	2.539	2.335	2.146	2.073	1.886	1.680	1.636	1.641	1.608	1.611	1.675	1.676	1.654	1.601	1.535	1.481	1.423	1.371	1.332	1.274	1.238
Uttar Pradesh	Lower	3.612	3.503	3.313	3.162	2.967	2.837	2.613	2.459	2.260	2.147	1.975	1.820	1.766	1.607	1.438	1.395	1.390	1.354	1.354	1.409	1.415	1.404	1.363	1.318	1.276	1.215	1.170	1.130	1.077	1.042
West Bengal	Upper	56.504	56.620	53.823	48.764	37.990	19.731	7.383	2.931	1.546	1.089	0.835	0.713	0.683	0.692	0.707	0.723	0.726	0.768	0.790	0.872	1.060	1.206	1.313	1.419	1.552	1.721	1.919	2.181	2.301	2.545
West Bengal	Point	42.286	41.980	41.134	37.160	27.660	13.988	5.201	2.209	1.273	0.916	0.718	0.616	0.578	0.573	0.577	0.583	0.580	0.613	0.621	0.670	0.779	0.893	0.994	1.108	1.235	1.411	1.593	1.808	1.900	2.110
West Bengal	Lower	34.781	34.770	30.108	27.068	20.715	7.392	2.295	1.357	0.952	0.730	0.587	0.507	0.476	0.477	0.476	0.472	0.461	0.470	0.471	0.481	0.515	0.589	0.670	0.761	0.867	1.013	1.199	1.423	1.553	1.738
Andaman & Nicobar Islands	Upper	34.101	13.033	11.585	8.091	5.985	4.095	3.081	2.954	2.901	2.713	2.670	2.705	2.413	2.403	2.532	2.453	2.460	2.277	2.362	2.316	2.266	2.233	2.222	2.275	2.210	2.188	2.285	2.388	2.176	1.972
Andaman & Nicobar Islands	Point	7.000	5.000	5.667	4.750	4.000	2.857	2.250	2.222	2.200	2.091	2.083	2.154	1.933	1.938	2.063	2.000	2.000	1.850	1.905	1.864	1.826	1.792	1.769	1.815	1.759	1.742	1.724	1.412	1.176	0.952
Andaman & Nicobar Islands	Lower	4.673	3.517	4.054	3.513	3.021	2.186	1.730	1.709	1.702	1.627	1.643	1.700	1.521	1.526	1.631	1.558	1.502	1.356	1.350	1.280	1.219	1.152	1.090	1.083	1.012	0.966	0.927	0.730	0.586	0.478
Chandigarh	Upper	82.089	97.386	78.225	55.979	40.271	34.943	27.641	21.824	17.689	13.245	8.638	6.604	4.610	3.103	2.192	1.730	1.733	1.394	1.274	1.044	0.998	0.971	1.049	1.155	1.161	1.180	1.239	1.344	1.504	1.546
Chandigarh	Point	29.494	34.000	29.500	26.250	21.750	20.231	16.619	11.939	8.404	5.400	3.095	2.160	1.528	1.130	0.981	0.888	0.966	0.881	0.879	0.765	0.725	0.704	0.759	0.827	0.837	0.868	0.931	1.009	1.141	1.180
Chandigarh	Lower	18.233	21.317	18.548	17.170	14.375	8.999	3.122	1.567	1.123	0.860	0.633	0.542	0.462	0.409	0.408	0.388	0.412	0.383	0.373	0.331	0.334	0.325	0.360	0.404	0.407	0.436	0.472	0.529	0.629	0.681
Dadra & Nagar Haveli	Upper	23.337	17.045	10.327	5.024	5.693	6.898	4.035	3.976	5.236	3.896	4.278	3.836	4.499	4.126	4.122	4.100	4.137	4.320	4.539	4.613	4.824	5.151	5.615	6.174	6.177	6.919	8.184	8.995	8.801	9.474
Dadra & Nagar Haveli	Point	7.833	7.465	5.547	3.192	4.000	5.000	3.000	3.000	4.000	3.000	3.333	3.000	3.500	3.200	3.167	3.143	3.125	3.222	3.400	3.455	3.583	3.769	4.077	4.385	4.286	4.643	5.538	6.273	6.455	6.727
Dadra & Nagar Haveli	Lower	5.177	5.298	4.139	2.455	3.163	3.999	2.445	2.432	3.284	2.470	2.755	2.458	2.808	2.501	2.389	2.275	2.182	2.162	2.200	2.141	2.116	2.103	2.081	2.100	1.928	2.036	2.445	2.839	3.136	3.376
Daman & Diu	Upper	35.614	36.432	40.136	32.930	37.391	33.212	29.111	27.495	25.424	20.332	17.694	13.484	9.048	6.257	5.426	3.934	2.572	1.822	1.441	1.170	1.597	1.615	2.030	2.270	2.361	2.383	3.021	3.168	3.563	3.707
Daman & Diu	Point	17.877	19.866	22.398	18.688	20.692	19.000	16.000	14.333	12.250	8.333	6.125	3.727	2.143	1.412	1.250	1.000	0.840	0.778	0.786	0.739	1.048	1.048	1.294	1.467	1.571	1.643	2.182	2.444	2.750	2.875
Daman & Diu	Lower	10.781	12.026	14.124	11.915	10.281	4.967	2.716	1.697	1.291	0.952	0.925	0.781	0.659	0.590	0.640	0.606	0.529	0.496	0.512	0.483	0.675	0.653	0.799	0.925	0.948	0.964	1.286	1.526	1.715	1.816
Puducherry	Upper	295.632	108.325	28.601	14.796	9.305	6.977	4.949	3.655	3.341	3.146	3.074	2.999	2.996	2.925	2.989	3.245	4.302	5.567	6.188	6.806	6.947	7.075	2.661	2.224	1.959	1.599	1.342	1.217	1.151	1.095
Puducherry	Point	17.600	11.556	8.615	6.500	5.125	4.129	3.103	2.435	2.259	2.177	2.186	2.156	2.176	2.161	2.235	2.477	3.213	4.384	4.887	5.292	5.313	5.442	1.970	1.643	1.453	1.221	1.059	1.008	0.962	0.881
Puducherry	Lower	1.349	0.715	0.913	1.192	1.086	0.927	0.694	0.566	0.558	0.581	0.664	0.745	0.865	1.035	1.332	1.618	2.171	3.199	3.653	4.006	3.799	3.762	1.380	1.156	1.025	0.859	0.744	0.697	0.654	0.589
Telangana	Upper	73.614	36.238	25.910	22.760	20.117	15.511	11.368	8.456	5.949	4.085	2.598	1.554	1.117	0.771	0.599	0.498	0.434	0.413	0.441	0.486	0.521	0.551	0.587	0.588	0.637	0.638	0.639	0.630	0.637	0.768
Telangana	Point	26.508	14.754	12.655	12.718	12.133	10.416	8.121	5.959	3.914	2.536	1.618	1.007	0.760	0.552	0.453	0.387	0.347	0.325	0.332	0.345	0.359	0.365	0.373	0.373	0.400	0.409	0.414	0.419	0.425	0.525
Telangana	Lower	4.363	4.334	5.105	6.351	5.700	2.971	1.678	1.189	0.868	0.719	0.591	0.469	0.423	0.356	0.316	0.285	0.252	0.223	0.221	0.221	0.217	0.211	0.206	0.200	0.209	0.213	0.213	0.226	0.230	0.254
India	Upper	103.665	68.211	46.023	28.212	14.128	8.966	6.736	5.283	4.117	3.000	2.124	1.488	1.095	0.838	0.688	0.596	0.546	0.530	0.557	0.612	0.668	0.719	0.753	0.798	0.855	0.880	0.899	0.930	0.954	1.014
India	Point	8.031	5.840	4.855	4.651	4.397	4.149	3.797	3.303	2.770	2.107	1.579	1.157	0.893	0.711	0.602	0.534	0.497	0.481	0.497	0.533	0.569	0.609	0.634	0.671	0.721	0.743	0.762	0.800	0.828	0.881
India	Lower	2.892	2.764	2.771	2.893	2.847	2.820	2.696	2.325	1.852	1.402	1.119	0.870	0.724	0.606	0.536	0.487	0.455	0.437	0.446	0.476	0.501	0.533	0.549	0.578	0.621	0.638	0.656	0.689	0.712	0.754
