# Supplementary material for: Diversity in HIV epidemic transitions in India: An application of HIV epidemiological metrices and benchmarks
Source: PLoS One. 2022 Jul 18;17(7):e0270886. doi: 10.1371/journal.pone.0270886 (PMC9292090; doi:10.1371/journal.pone.0270886)
Supplement: Attachment — Submitted filename: Authors response to reviewers comments .docx [file pone.0270886.s003.docx]

**Response to the observations of reviewers in the context of the manuscript ID “[PONE-D-21-34403] - [EMID:49e8df8c235ee894]” titled "Diversity in HIV Epidemic transitions in India: An application of HIV epidemiological metrices and benchmarks"**

***

**Response to the comments from Reviewer**

We have gone through the observations of the reviewer and editor for our manuscript ID “[PONE-D-21-34403] - [EMID:49e8df8c235ee894]” titled "Diversity in HIV Epidemic transitions in India: An application of HIV epidemiological metrices and benchmarks".

We thank the reviewers for their constructive comments and we believe that we are now able to provide more clarity to the readers.

We have provided a detailed point-by-point response to all observations/comments (reviewer’s/editor’s comments in black, our replies in blue). Line numbering refers to the revised manuscript.

**Reviewer’s Observations/Comments and Authors’ Response**

1. General Observation/Comment

I’d like to thank the authors for the opportunity to review their manuscript. This is an important topic that is well explored by the paper. I recommend it for publication with a few suggested improvements/comments.

Reply: We sincerely thank the reviewer for appreciation of our work.

2. Technical Comments

(i) When discussing disease control, the ideal metric to be monitored is Rt. IPR and IMR are important proxies that help measure the growth or reduction of disease in a population. I think the authors do a good job of explaining why we should care about these metrics and how they relate to epidemic control. However, I think it would be useful to add information about their limitations.

Reply: We thank the reviewer for a clear guidance. We have added the limitations in the revised manuscript [line number ‘244’ to ‘266’].

(ii) The relation between IPR, epidemic control and the .03 target assumes that secondary infections are equally likely to occur at any time during the (on average) 30 years of time an individual is infected. While a useful assumption, this is in general not the case. Scale up of test and treat has reduced the infectivity of diagnosed individuals so that long term infections are less likely to transmit than short term ones. The result is that epidemic control may be reached at much higher than .03 IPR.

Reply: We agree with the reviewer. We have added this context in the revised manuscript [lines 244-251].

(iii) For IMR, changing the time between infection and death can create trends that have little to do with epidemic control. I believe you see this in the India data. The scale-up of ART therapy reduces mortality, which shrinks the denominator. This can create an upward trend in IMR even if incidence is decreasing. I interpret the upward trend after 2005 as an artifact of this.

Reply: We thank the reviewer for this insight. Although we had alluded to this point in paragraph 2 of the discussion section in our original manuscript, we have now included this additional perspective in the limitations of IMR in high ART coverage settings [lines 260-266 of the revised manuscript].

3. Other minor points

(a) If you can, it would be great if the images could be as high of quality as possible. I found myself zooming in on the individual graphs and they were readable, but could get a bit pixelated.

Reply: In pursuance of the suggestion of the reviewer, we have now updated the legends of the graphs clearly stating which colour of the line graph represents which indicator for the ease of understanding of the reader. The legend has been also updated to reflect the items on the X and Y-axis.

(b) 95-103: These equations are unreadable. It took me a while to realize that they were fractions. I thought they were formatting errors.

Reply: We thank the reviewer for this valuable suggestion. We have now presented the equations in an improved format by using the insert equation function. The revised equations are on lines 138 and 140.

(c) 164: This is too speculative. It may happen and the data may support continued reduction, but we shouldn’t just expect that HIV will go away.

Reply: We fully agree with the reviewer. We have edited the statement appropriately [line 217 of the revised manuscript].

(d) 119: I don’t see supplementals. Did you mean figures?

Reply: We had added the supplement at the time of original submission, but perhaps it was not accessible. We have uploaded the supplement again.

4. Grammer

Consider doing a close read through for grammar. Here are a few things I noticed.

a. 7: remove second “on”
b. 43: doesnot —> does not
c. 43: add “in” after “factor”
d. 59: delete “will”
e. 68: delete “.”
f. 69: Method —> Methods
g. 71: states —> state

Reply: We thank the reviewer and made changes as suggested. We have carefully reviewed our revised manuscript.
