# Supplementary material for: Diversity in HIV epidemic transitions in India: An application of HIV epidemiological metrices and benchmarks
Source: PLoS One. 2022 Jul 18;17(7):e0270886. doi: 10.1371/journal.pone.0270886 (PMC9292090; doi:10.1371/journal.pone.0270886)
Supplement: Attachment — Submitted filename: Response to Reviewers.docx [file pone.0270886.s004.docx]

PONE-D-21-34403R1

Diversity in HIV Epidemic transitions in India: An application of HIV epidemiological metrices and benchmarks

PLOS ONE

***

**Response to Reviewers**

We have gone through the observations of the reviewer and editor for our manuscript ID “[PONE-D-21-34403] - [EMID:49e8df8c235ee894]” titled "Diversity in HIV Epidemic transitions in India: An application of HIV epidemiological metrices and benchmarks".

We thank the academic editor/reviewer(s) for their constructive comments. We believe the review has enhanced the rigour of the manuscript.

We have provided a detailed point-by-point response to all observations/comments (reviewer’s/editor’s comments in black, our replies in blue). Line numbering refers to the revised manuscript.

**Academic Editor Observations/Comments and Authors’ Response**

1. Comment 1:

The data source used, 'India HIV Estimates 2019' shows very large amounts of uncertainty in the estimates of numbers of PLHIV, HIV incidence, and HIV mortality. This uncertainty needs to be carried through into the estimates presented in the manuscript, as the results cannot be correctly interpreted without confidence intervals. The level of uncertainty in the results should also be reflected in the interpretation of the results and the language used throughout the manuscript. For instance, statements such as "Ten States/ UTs of Andaman ... had estimated IMR of one or less." should not be made if the confidence intervals overlap one.

Reply:

We agree that we have provided only the point estimates for IPR and IMR. We concur with the Editor observations of providing uncertaintly bounds. Adding uncertaintly bounds on IPR and IMR will not only increase the scientific rigour of the manuscript but also improve inferences as noted by the Editor.

The current manuscript is based on the outputs generated using Spectrum Model under 2019 round of HIV burden estimates in India. The Spectrum model calculates uncertainty bounds around each estimate including that on new infections and deaths among PLHIV. However, we were of the opinion that using lower bounds or upper bounds of new infections, deaths and PLHIV to create uncertainty bound around IPR and IMR may not be the right approach as these would be applied for both numerator and denominator.

We noted that UNAIDS publications have provided IPR with uncertaintly bounds though method for the same was not described. And hence we reached to Dr Keith Sabin (Strategic Information Department, UNAIDS, Geneva, Switzerland) to understand the calculation of uncertaintly bounds. Dr Sabin informed that proportional uncertainty around the new infections is used to calculate the uncertainty bound around IPR and IMR. To be consistent with globally used methodology, we applied the same for our manuscript. This method has been stated in Line Number 136-138 of the revised manuscript.

The Results section has been updated to include the uncertainty bounds around tables and figures. The table 1, beginning at Line Number 177, now present State/UT-wise IPR and IMR with uncertainty bounds. Besides all the relevant paragraphs (Line Number 159-165, Line Number 180-200) in the results sections have results with uncertainty bounds.

We have revised the inferences and language of the discussions in view of the insights provided by uncertainty bounds. The revision is reflected in Line Number 208 to 239 of the revised manuscript.

We have noted the wider uncertainty bounds as one of the limitation (Line Number 270-277).

2. Comment 2:

The new paragraph starting from line 244 is not correct. I appreciate that it was added in response to a suggestion made by a reviewer, but it is not necessary to make changes suggested by reviewers if they are not factually correct. The prevalence of HIV will grow in a population if the rate of new infections is higher than the rate at which people are removed from the pool of prevalence infections. The time of transmission relative to the duration of disease does not effect the interpretation of the IPR in such a simplistic way. I think the mistake the reviewer was making was in thinking that if people mostly only transmit in the first 5 years following infection, the the IPR at which control is achieved should be 1/5, not 1/30. What that misses is that in that case only prevalent infections in the first 5 years following infection should be included in the numerator for the IPR, and therefore the two factors will cancel out.

Reply:

We do see the point mentioned by the academic editor. In fact, we reviewed the UNAIDS report titled ‘MAKING THE END OF AIDS REAL: CONSENSUS BUILDING AROUND WHAT WE MEAN BY EPIDEMIC CONTROL’ and paper titled "Epidemiological metrics and benchmarks for a transition in the HIV epidemic’ by Ghys, Peter D., et al. We noted that both of these refers to threshold of 0.03 as more of a ‘rule of thumb’ and indicated for more discussions around the threshold. As ART uptake has significant impact on transmission of new infections, we believe that this is area that may be included as one of the points in future discussions on threshold value and behaviour of IPR.

In view of above, we have retained the reference to potential impact of ART on IPR threshold. However, we have removed the sentence ‘As a result, the epidemic control may be reached at much higher than 0.03 IPR’ to avoid any comments on the threshold level of epidemic control in our manuscript. We have added a sentence ‘Impact of ART uptake on the IPR may be one of the factors while deliberating the threshold and behaviour of IPR’ (Line Number 250-251 of the revised manuscript).
