# Supplementary material for: Diversity in HIV epidemic transitions in India: An application of HIV epidemiological metrices and benchmarks
Source: PLoS One. 2022 Jul 18;17(7):e0270886. doi: 10.1371/journal.pone.0270886 (PMC9292090; doi:10.1371/journal.pone.0270886)
Supplement: Attachment — Submitted filename: Response to Reviewers.docx [file pone.0270886.s005.docx]

PONE-D-21-34403R2

Diversity in HIV Epidemic transitions in India: An application of HIV epidemiological metrices and benchmarks

PLOS ONE

***

**Response to Reviewers/Editor**

We have gone through the observations of the reviewer and editor for our manuscript ID “PONE-D-21-34403R2” titled "Diversity in HIV Epidemic transitions in India: An application of HIV epidemiological metrices and benchmarks".

We thank the Academic Editor for very constructive comments. We believe the review has enhanced the rigour of the manuscript.

We have provided a detailed point-by-point response to all observations/comments (editor’s comments in black, our replies in blue). Line numbering refers to the revised manuscript.

**Academic Editor Observations/Comments and Authors’ Response**

1. Comment 1:

Thanks for adding in uncertainty estimates, they greatly increase the potential usefulness and applicability of the work. Unfortunately, the way they are calculated is not quite correct (based on the way it is described in the methods). It takes into account the uncertainty resulting from the uncertainty in the incidence estimates, but not the uncertainty resulting from the mortality and prevalence estimates. This could mean that the uncertainty is underestimated. Alternatively, if the incidence and mortality/prevalence estimates are correlated between model runs, it may mean that the uncertainty is overestimated.

Reply:

We agree with the comment.

2. Comment 2:

I have not used the Spectrum models myself. Is it possible to export the estimates from individual model runs, using the uncertainty analysis tool? If it is possible, the simplest way to obtain better estimates of the uncertainty would be to divide the incidence by the mortality/prevalence estimate separately for each individual model run, and then to obtain the 2.5% and 97.5% percentiles for the ratios to give 95% uncertainty intervals. Please make sure that the uncertainty intervals that this approach generates seem reasonable to you however, it is possible that the way that the Spectrum model is parameterised and calibrated would mean that this approach would generate unreasonably narrow uncertainty intervals.

Is this is not possible, then there may not be any better approach than the one used, but please discuss the limitations of the way that the uncertainty intervals are generated in the Discussion section.

Reply:

We thank the Academic Editor for an extremely useful suggestion. The ‘Uncertainty Analysis’ tool in Spectrum indeed generates plausible range by running 1000 iterations. Results of each of the iteration for key indicators may be exported. Given this provision, we revised the method for calculating the uncertainty bounds. The revision has been suitably reflected under Method [Line Number 120-124, 145-153 of revised manuscript], Results [Line Number 170-175, 194-223 of revised manuscript] and Discussions [Line Number 240, 243-245, 249-261 of revised manuscript].

3. Comment 3:

I agree entirely that the threshold of 0.03 is a rule of thumb only, and feel that a paragraph discussing this is important. What I do not understand is this sentence: “the 0.03 target assumes that secondary infections are equally likely to occur at any time during the (on average) 30 years of time an individual is infected”. As far as I can see, the references you provided in your response say nothing about the target assuming this, and I do not understand how it does. Please explain this statement and/or provide a reference for it, or remove it. It could be replaced with a brief discussion of the major assumption made in setting the threshold: that people live with HIV for a mean of 30 years.

Reply:

We have revised the paragraph suitably [Line Number 268-272 of revised manuscript].
